# Fungal Naphthalenones; Promising Metabolites for Drug Discovery: Structures, Biosynthesis, Sources, and Pharmacological Potential

**DOI:** 10.3390/toxins14020154

**Published:** 2022-02-19

**Authors:** Sabrin R. M. Ibrahim, Sana A. Fadil, Haifa A. Fadil, Bayan A. Eshmawi, Shaimaa G. A. Mohamed, Gamal A. Mohamed

**Affiliations:** 1 Department of Chemistry, Preparatory Year Program, Batterjee Medical College, Jeddah 21442, Saudi Arabia; 2 Department of Pharmacognosy, Faculty of Pharmacy, Assiut University, Assiut 71526, Egypt; 3 Department of Natural Products and Alternative Medicine, Faculty of Pharmacy, King Abdulaziz University, Jeddah 21589, Saudi Arabia; safadil@kau.edu.sa (S.A.F.); gahussein@kau.edu.sa (G.A.M.); 4 Department of Clinical and Hospital Pharmacy, Faculty of Pharmacy, Taibah University, Almadinah Almunawarah 30078, Saudi Arabia; hfadil@taibahu.edu.sa; 5 Department of Pharmaceutics, Faculty of Pharmacy, King Abdulaziz University, Jeddah 21589, Saudi Arabia; beshmawi@kau.edu.sa; 6 Faculty of Dentistry, British University, El Sherouk City, Suez Desert Road, Cairo 11837, Egypt; shaimaag1973@gmail.com

**Keywords:** naphthalenones, fungi, polyketides, biosynthesis, phytotoxic, bioactivities

## Abstract

Fungi are well-known for their abundant supply of metabolites with unrivaled structure and promising bioactivities. Naphthalenones are among these fungal metabolites, that are biosynthesized through the 1,8-dihydroxy-naphthalene polyketide pathway. They revealed a wide spectrum of bioactivities, including phytotoxic, neuro-protective, cytotoxic, antiviral, nematocidal, antimycobacterial, antimalarial, antimicrobial, and anti-inflammatory. The current review emphasizes the reported naphthalenone derivatives produced by various fungal species, including their sources, structures, biosynthesis, and bioactivities in the period from 1972 to 2021. Overall, more than 167 references with 159 metabolites are listed.

## 1. Introduction

Fungi are the second-biggest group of organisms after insects [1]. Many fungal species have a wide range of biotechnological and industrial potential [2,3,4,5,6,7]. They are acknowledged as one of the wealthiest pools of natural metabolites among living organisms due to their unique metabolic system and their capacities to synthesize diverse kinds of metabolites with quite intriguing chemical skeletons [8,9]. These metabolites possess a wide range of applications as agrochemicals, antibiotics, immune-suppressants, anti-parasitic, and anticancer agents [10,11,12,13,14,15,16,17,18,19,20]. Naphtalenones are among the naphthalene derivatives produced by fungi that are strictly related to napthoquinones and involved in the branched pathway of fungal DHN (1,8-dihydroxynaphthalene)-melanin biosynthesis [21,22]. Also, they belong to a group of renowned phytotoxins produced by various crop and forest plants pathogenic fungi [22,23,24]. Moreover, naphthalenone derivatives possess a great structural diversity not only in the planar structure but also in the absolute configuration. Many reported reviews mention naphthalenones only as fungal phytotoxins [22,23,24]. In fact, they have other interesting bioactivities in addition to their phytotoxic activity such as antiviral, antimicrobial, nematocidal, antimycobacterial, cytotoxic, antimalarial, and inhibitory enzyme activities. To our knowledge, no detailed review about fungal naphthalenone derivatives has been published. Thus, this review aims at giving an overview of reported fungal naphthalenone derivatives, their structures, sources, biosynthesis, and bioactivities in the period from 1972 until 2021 (Supplementary Material Tables S1 and S2). Their fungal source, molecular weights and formulae, hosts, and location of the reported naphthalenone derivatives have been listed in Table S1. It was observed that some errors and overlapping in the compound names have arisen, where there are multiple names for one chemical structure. Therefore, the additional names for compounds have been added in brackets (Table S1). Also, the results of the biological activities of the most active derivatives have been summarized in Table S2. Highlighting the bioactivities of these metabolites may attract attention to the synthesis of new agents by medicinal and synthetic chemists using the known naphthalenones as start materials. Literature searching for the published studies was accomplished through diversified databases: PubMed (MedLine), Web of Science, GoogleScholar, SciFinder, and Scopus, as well as through different publishers; Springer-Link, Wiley, Taylor & Francis, Bentham, and ACS (American Chemical Society) Publications utilizing keywords (naphthalenone, fungi, biosynthesis, and activities).

### Biosynthesis of Naphthalenones

**Scheme 1:**
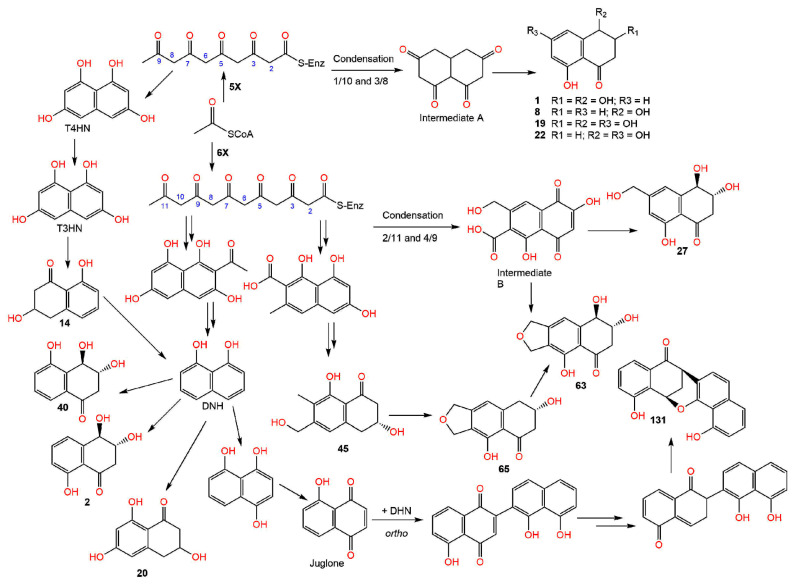
Biosynthesis of compounds **1**, **8**, **19**, **20**, **22**, **27**, **45**, **63**, **65**, and **131** [21,26,27].

Naphthalenones have been inferred to be biosynthesized in melanin-forming fungi from DHN (1,8-dihydroxynaphthalene) as precursors via acetogenic pathway (Scheme 1). Therefore, these metabolites have a role in fungal melanin biosynthesis. DHN biosynthesis begins with the pentaketide chain. Subsequently, T4HN (1,3,6,8-tetrahydroxynaphthalene) is produced, which is changed to T3HN (1,3-8-trihydroxy naphthalene) via dehydration and reduction reactions. The reduction of T3HN yields vermelone (14), which is changed to DHN by dehydration reaction [13,23,24,25,26]. Zhang et al. reported that the polyketide synthesis was started by respective condensations of five acetate units that give compounds **1**, **8**, **19**, and **22,** and of six acetate units producing compounds **27** and **63** [27] (Scheme 1). Barnes et al. reported that the regiodivergent folding of the hexaketide chain gives rise to various bicyclic cores [21] and various tailoring reactions, particularly the oxido-reductions produce **2**, **40**, **45**, **63**, and **65**, as well as juglone and DHN (Scheme 1).

**Scheme 2:**
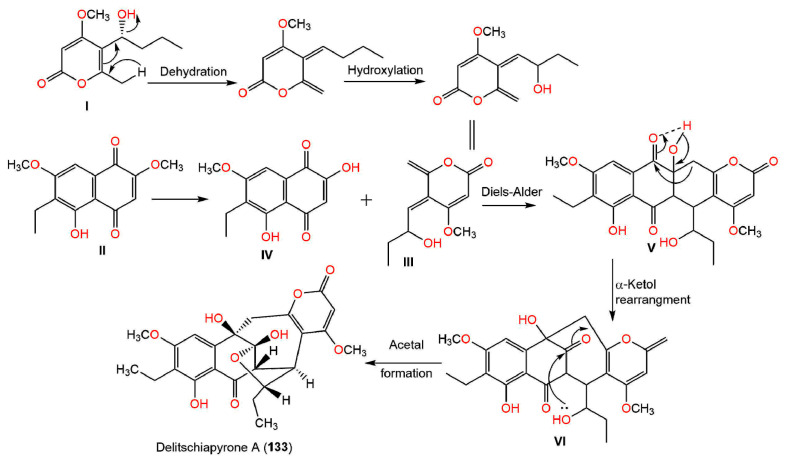
Biosynthesis of compound **133** [28].

Further, naphthalenones are also strictly related to napthoquinones, therefore, several naphthoquinones are linked to the biosynthesis of naphthalenones [23,24,26]. Moreover, it was postulated that **131** results from region-isomeric ortho-coupling of DHN and juglone. The C-O-bridged strained ring system may be produced by a reduced intermediate condensation or by an intra-molecular addition reaction, including a quinone-methide precursor [21]. Luo et al. postulated that the biosynthesis for **133** involves a Diels–Alder addition, followed by an α-ketol kind of rearrangement [28]. Accordingly, the Diels–Alder reaction of the diene **III** produced from **I** and the O-demethyl derivative of **II** (**IV**) yields the 6/6/6/6-fused tetracyclic intermediate **V**, which undergoes an α-ketol rearrangement, giving the 6/6/7/6 tetracyclic intermediate **VI**. Subsequently, **VI** undergoes a cyclization between the C-3 carbonyl and 20-OH, resulting in an acetal and forming the tetrahydrofuran ring of **133** [28] (Scheme 2).

**Scheme 3:**
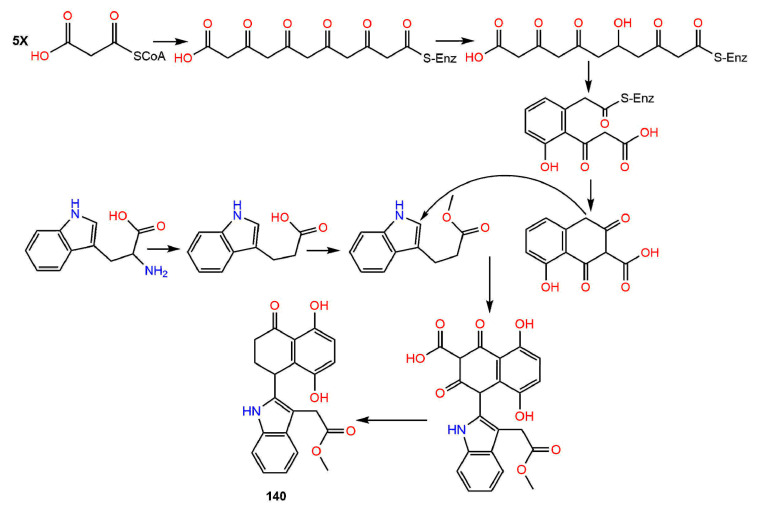
Biosynthesis of compound **140** [29].

Moreover, the possible biosynthetic pathway of **140** was proposed by Li et al., who proposed that the indole unit was derived from tryptophan, however, the naphthalenone unit was derived from propionyl-CoA. The key step in this pathway is the building up of the C-C bond between C-11 and C-4 of the two units [29] (Scheme 3).

## 2. Bioactivities **of** **Naphthalenones**

The reported naphthalenones have been investigated for various bioactivities. In this regard, these metabolites have been associated with many types of bioactivities, including phytotoxic, antiviral, antimicrobial, nematocidal, antimycobacterial, cytotoxic, antimalarial, anti-inflammatory, insecticidal, and alpha-glucosidase and IDO inhibitory activities. Herein, these activities have been discussed and results of the most active metabolites have been listed in Table S2.

### 2.1. Phytotoxic and Nematocidal Activities

**Figure 1:**
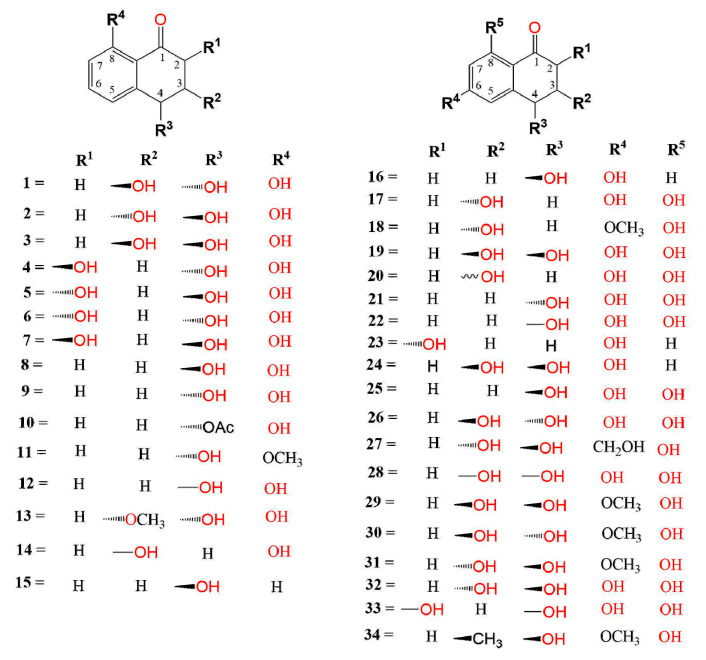
Structures of compounds **1**–**34**.

Weeds represent the most common and severe biotic factors affecting agriculture and are responsible for remarkable agricultural losses. They have negative effects on the crop plants because of the competition for water, nutrients, sunlight, and space. Additionally, they can be a reservoir for certain plant pathogenic microorganisms and/or herbivorous insects [30]. Integrated management strategies are generally applied for controlling weeds by using mechanical methods along with synthetic herbicides [22]. However, the extensive use of synthetic herbicides has toxic effects not only on the target organism but also on animals and humans, in addition to the adverse environmental impacts and promotion of the emergence of herbicide-resistant species. Therefore, research interest has been directed toward identifying new bioherbicides of natural origin [31]. Fungi are known to have the capacity to produce diverse arrays of secondary metabolites that could be beneficial as bioherbicidal agents [22]. Fungal phytotoxic metabolites have a crucial role in developing disease symptoms in host plants. Although they can cause significant damage to crops, naphthalenones can also function as starting material for the development of natural herbicides to control the growth and spread of weeds [32]. Interestingly, many of the reported naphthalenones have been found to possess phytotoxic potential. These phytotoxic properties could be utilized for developing simple, rapid, and specific tools to identify plant diseases such as a test kit (e.g., rapid test strip) that can be used directly by farmers in the field. Additionally, they can be used as lead compounds by allowing the synthesis of more phytotoxic compounds on a range of weeds based on their structures for potential application as herbicides. Compound 1 isolated from *Pyricularia Oryzae* reduced the rice seedlings’ growth at high concentrations, however, it slightly stimulated the rice seedlings’ growth at a concentration of 100 ppm in 24 h [33]. Masi et al. reported that the culture filtrate of *Pyricularia grisea* afforded **2** and **9** (Figure 1), which were assessed for their phytotoxicity toward buffelgrass (*Cenchrus ciliaris*) using radicle elongation and buffelgrass coleoptile bioassay. They significantly delayed the seed germination relative to the control, whereas **2** also apparently reduced the germination percentage [34].

Moreover, **2** inhibited *Lepidium sativum* and *Setaria italica* germinated seed growth (IC_50_ 50 and 100 µg/disc, respectively) [35]. Further, the cup fungus, *Urnula craterium* yielded **4** and **9** that had no in-vitro activity toward the aspen pathogens: *Ophiostoma piliferum*, *O. crassivaginatum*, and *Populus tremulae* (conc. up to 100 µg/mL) [36]. Also, it was found that **9** (conc.1–10 ppm) stimulated the rice seedlings’ root elongation by ≈30%, whereas **8** did not have any stimulating effect. On the other hand, both **8** and **9** prohibited (Conc. 50 ppm) the rice seedlings’ shoot and root growth with readily observable chlorosis (white spots) on leaves [33,37].

Additionally, 1, 9, and 21 were separated from the liquid culture of *Tubakia* *dryina*, the causative agent of *Quercus rubra* (red oak) leaf spot. In the detached leaf assay, these metabolites caused large lesions on the red oak leaves that developed along the veins within 24 h, very similar to those resulting from *T. dryina* infection. On the other hand, they had a moderate phytotoxic effect on white oak, prickly sida, and sorghum [38].

**Figure 2:**
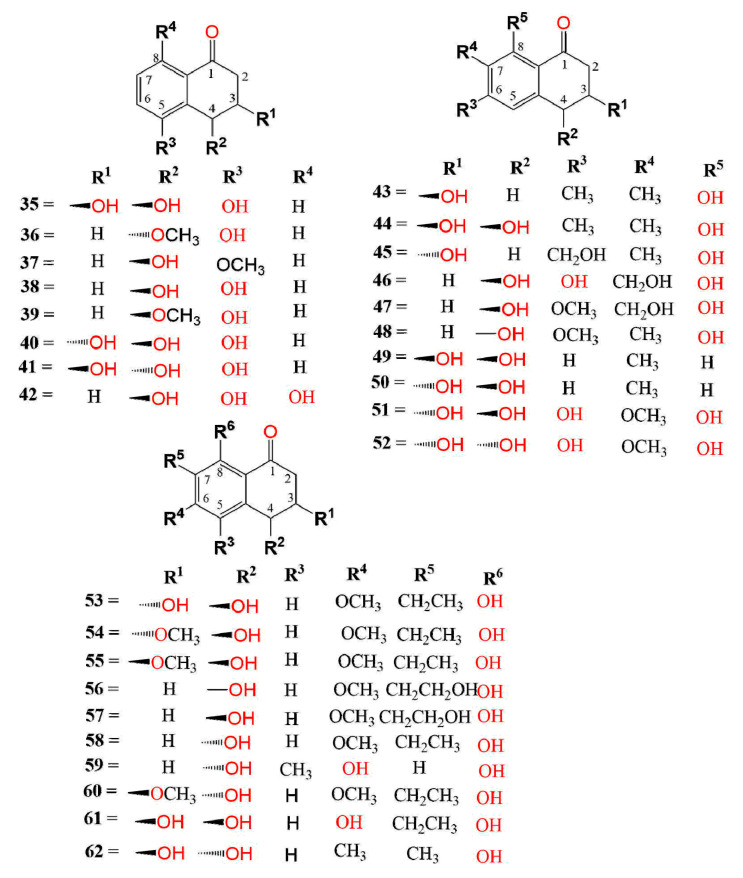
Structures of compounds **35**–**62**.

The pathogenic fungus, *Ceratocystis fimbriata* f. sp. *platani* that caused canker stain in the plane tree (*Platanus acerifolia*) produced **4** and 5, which produced large necrotic lesions in the plane tree tissues at a concentration of 1.0 mg/mL, whereas 17 possessed significant necrosis only after 7 days, whereas 19 and 28 exhibited less activity after 48 h [39]. Compound **7** was separated from *Mycosphaerella fijiensis* IMI 105378, the causative agent of Black Sigatoka disease in plantains and bananas. It induced necrotic lesions (Conc. 5 µg/5 µL) in <12 h on the sensitive cultivars of bananas in the leaf-puncture bioassay [40]. *Ceratocvstis fimbriata* that is accountable for the canker disease of the coffee tree yielded **14** and **17** that exhibited no remarkable toxic effect on coffee trees (conc. 1 × 10^−3^ M) [41]. Furthermore, **12** and **17** were separated from *Phaeoacremonium aleophilum* associated with the esca of grapevine. Compound **12** (Conc. 0.1 mg/mL) produced large, coalescent necrotic, and chlorotic spots then withering and distortion of the lamina, however, **17** (Conc. 0.05 mg/mL) produced light green to chlorotic, rounded to irregular, inter-veinal, or marginal spots on the grapevine detached leaves. Thus, they caused similar symptoms to those shown by the vines leaves with brown wood-streaking that is associated with wood infection by *P. chlamydosporum* and *P. aleophilum* [42]. Moreover, **17** separated from *Raffaelea quercivora*, which caused the Japanese oak wilt disease, inhibited (Conc. 100 µg/mL) the lettuce seedlings’ root growth to 54.8% of the negative control [43]. On the other hand, **22** slightly promoted the second leaves growth (Conc. 500 ppm) and **33** possessed no noticeable activity on the growth of rice seedlings [33,37]. Whilst **48** had a weak phytotoxic activity toward *Lepidium sativum* (IC_50_ 100 µg/disk) [44]. The new naphthalenone; botrytone (**42**), along with the formerly separated **3**, **4**, and **8** were purified from the culture filtrate of *Botrytis fabae* associated with *Vicia faba* (fava bean). Compound **8** displayed the highest phytotoxicity together with **3** and **4** on the *Vicia faba* leaves, however, **42** had moderate potential (Conc. 1 mg/mL) [44]. *Neofusicoccum australe* strain BL24 (haplotype H1) produced **31** and **53** (Figure 2).

They were assessed for phytotoxic activity on the leaves of holm oak, cork oak, and grapevine leaves utilizing leaf-puncture assay (Conc. 0.125, 0.25, 0.5, and 1 mg/mL). Compound **53** was much less toxic even at the highest concentration. It caused necrotic lesions on the leaves of cork oak, holm oak, and grapevine (area lesions 4.8, 3.3, and 11.9 mm^2^, respectively). On the other hand, **31** did not possess any phytotoxic effect [45]. *Neofusicoccum parvum* is one of the most virulent botryosphaeriaceous species that affect the grapevine. Investigation of its extract yielded **9**, **40**, **53,** and **94** that were found to be phytotoxic on grapevine leaves in the leaf puncture assay, with **53** having the greatest potential [46]. Masi et al. purified lentiquinones B (**115**) and C (**116**) from the culture filtrate of *Ascochyta lentis* separated from the diseased lentil (*Lens culinaris*). Both compounds had the same structure but differed in C-2-OH group configuration, showing α- and β-configuration, respectively. They featured three six-membered rings skeleton, involving trihydroxy-cyclohexene and hemi-quinone rings. Their absolute configuration was assigned as 2R,3S,4S,4aS,10R and 2S,3S,4S,4aS,10R, respectively using X-ray, ECD (electronic circular dichroism), and TDDFT (time-dependent DFT) calculations. They showed strong phytotoxicity toward *Lupinus albus* and *Chenopodium album* in the leaf puncture assay [47]. Compound **132** biosynthesized by *Guignardia laricina* inhibited the growth of lettuce seedling roots by 71.5, 16.2, and 7.0% at concentrations of 100, 250, and 50 ppm, respectively in the lettuce seedling bioassay [48].

Plant-parasitic nematodes are plant pathogens that can cause significant reductions in agricultural yields resulting in substantial annual economic losses to growers [49]. Chemical nematocidal agents such as organophosphorus and carbamate are used for controlling these parasitic nematodes, however, their long-term use can result in increased nematode resistance, as well as deleterious effects on human health [50]. Recently, research has emphasized the discovery of nematocidal agents from natural sources including the nematocidal potential of naphthalenone derivatives. Therefore, some studies reported the nematocidal potential of naphthalenone derivatives.

Compounds **9** and **22** isolated from an unidentified freshwater fungus YMF 1.01029 exhibited weak nematocidal potential toward the nematode *Bursaphelenchus xylophilus* [51]. The four naphthalenones; **8**, **9**, **19**, and **22** separated from the cultural extract of *Caryospora callicarpa* were assessed in-vitro for antinematodal activity toward *Bursaphelenchus xylophilus* (fungal-feeding and plant-parasitic nematode) in the nematotoxin bioassay. They showed noticeable nematocidal potential, which was significantly enhanced with the exposure times length at the same concentration (LC_50_s (lethal concentration 50) 209.7, 229.6, 220.3, and 206.1 mg/L, respectively at 36 h exposure). Their mode of action was suggested to be systemic, instead of contact poisons or antifeedants [52].

### 2.2. Antimicrobial, Antimycobacterial, and Anti-Plasmodial Activities

Infectious diseases are a worldwide health problem. Multidrug-resistant (MDR) pathogens remarkably increase morbidity and mortality rates [53]. The continuous emergence of MDR pathogens drastically reduced the efficacy of antibiotics resulting in a growing rate of therapeutic failure [54]. Accordingly, new and effective antimicrobial agents to address microbial infections are needed.

Inácio et al. purified **3** and **8** from *Cryptocarya Mandioccana* healthy leaves associated with *Colletotrichum gloeosporioides* by RP-HPLC (reversed phase-high performance liquid chromatography) and evaluated their antifungal activity by direct bioautography on TLC (thin layer chromatography) plate, which includes spraying the fungal suspensions on the developed TLC plates utilizing solvents of different polarities for detecting the antifungal potential of these compounds [55]. The effectiveness was indicated by white spots against a red-purple background on the TLC plates after spraying with tetrazolium violet [56]. It was found that the required detection limit of these compounds for inhibiting the growth of the phytopathogenic fungi: *Cladosporium sphaerospermum* and *C. cladosporioides* was 5.0 mg, compared with nystatin [55]. On the other hand, **2** and **17** obtained from *Lachnellula* sp. cultures had no antimicrobial potential (Conc. 100 µg/mL) in the serial broth dilution assay toward *A. calcoaceticus*, *M. luteus*, *M. miehei*, and *P. variotii* [35]. Findlay and Kwan stated that **9** and **17** purified from *Scytalidium* FY had significant antifungal activity [57]. On the other side, **9** had weak activity against *B. subtilis* (IC_50_ 100 µg/mL), compared with chloramphenicol (IC_50_ 3.13 µg/mL) in the colorimetric assay [58] and no activity toward *M. smegmatis*, *S. aureus*, *S. cerevisiae*, *C. neoformans*, *C. albicans*, *E. coli*, *A. niger*, and *Micrococcus luteus* [59]. Lu et al. separated **10** from *Cytospora* sp. isolated from *Ilex canariensis* that showed antibacterial activity (IZD (inhibition zone diameter) 15.0 mm) toward *Bacillus megaterium* in the agar diffusion assay, compared with penicillin (IZD 28.0 mm) [60].

Pittayakhajonwut et al. purified **9**, **13**, **14**, **22**, **28**, and **31** from *Phaeosphaeria* sp. Compounds **13**, **22**, and **31** possessed significant anti-mycobacterial activity (MICs (minimum inhibitory concentrations) 12.5, 12.5, and 25.0 μg/mL, respectively), whereas **9** and **14** had moderate effectiveness (MIC 50.0 μg/mL), compared to isoniazid and kanamycin (MICs 0.05 and 2.5 μg/mL, respectively) using the micro-plate Alamar blue assay (MABA) [61]. On the other side, **2** had no noticeable antifungal effect toward *C. albicans* (MIC > 128 µg/mL) in the XTT (cell proliferation kit II) assay [62].

The new derivative, **25**, and the formerly reported **1** and **3** were separated from the endolichenic fungus, *Xylariaceae* sp. CR1546C obtained from Costa Rican lichen *Sticta fuliginosa*. The C-4 R-configuration of **25** was deduced based on the Mosher method and the opposite optical rotation sign to that of similar structural metabolite—**21**. These metabolites exhibited weak antifungal potential toward *C. albicans* with MFCs (minimum fungicidal concentrations) between 100 and 150 µg/mL and IC_50_ from 60 to 100 µg/mL, compared to amphotericin B (IC_50_ 1.3 µg/mL) using broth-dilution technique [63].

The endophytic fungus *Daldinia eschscholtzii* associated with the mangrove plant *Bruguiera gymnorrhiza* yielded a new metabolite; (3*S*)-3,8-dihydroxy-6,7-dimethyl-a-tetralone (**43**), along with **9**. The C-3 absolute configuration of **43** was determined as S based on the CD spectrum. These compounds had weak antimicrobial potential toward *S. aureus*, *M. gypseum*, and MRSA (MICs 200 mg/mL) [64]. *Cladosporium* sp. JJM22 isolated from *Ceriops tagal* stem bark also yielded **43** that showed a broad spectrum of antibacterial potential versus *V. parahemolyticus*, *S. aureus*, *E. coli*, *V. alginolyticus*, *B. cereus*, and MRSA at a concentration of 20 μM [65].

**Figure 3:**
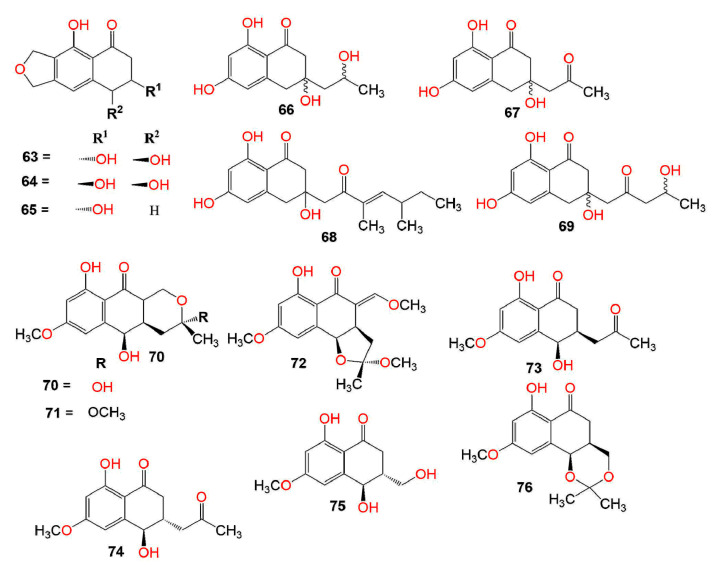
Structures of compounds **63**–**76**.

Compounds **44** and **64**, two new dihydronaphthalenones were purified from *Nodulisporium* sp. isolated from *Antidesma ghaesembilla* fresh twigs (Figure 3). Compound **64** had C6/C7 fused furan ring. The 3*R*,4*S* and 7*R*,8*S* stereo-configurations of these metabolites were deduced based on the coupling constants, NOE (nuclear overhauser effect), optical rotations, and X-ray analysis. Compound **64** exhibited anti-mycobacterial potential (IC_50_ 3.125 µg/mL) toward *M. tuberculosis* H37Ra and antimalarial effectiveness versus *P. falciparum* K1 (IC_50_ 11.3 µg/mL) using the GFPMA (green fluorescent protein micro-plate assay) and micro-culture radioisotope technique, respectively, however, **44** was inactive [66]. Moreover, **48** possessed weak antimicrobial activity (MIC 100 µg/mL) toward *M. luteus*, *E. dissolvens*, *M. miehei*, *P. variotii*, and *P. notatum* in the serial broth dilution assay [44].

The new derivative, **61**, separated from the aquatic fungus *Delitschia corticola* was reported to have moderate antifungal potential toward *Sclerotium* sp. YMF-1.01993, *Alternaria* sp. YMF-1.01991, and *Fusarium* sp. YMF-1.01996 (IZDs 8.0, 7.0, and 7.0 mm, respectively), compared to ciclopirox (IZDs 26.0, 19.0, and 20.0 mm, respectively), however, it had moderate antibacterial effectiveness toward *B. cereus* YMF-3.19 and *B. laterosporus* YMF-3.08 (IZDs 12.0 and 10.0 mm, respectively), compared to ampicillin sodium (IZDs 35.0 and 30.0, respectively) and stronger activity (IZD 20.0 mm) more than ampicillin sodium (IZD 18.0 mm) toward *S. aureus* YMF-3.17 [67].

Compounds **66**, **67**, and **69** had no anti-malarial effectiveness toward *P. falciparum* K1 [68]. Moreover, they showed no anti-mycobacterial activity versus *M. tuberculosis* H37Ra and no antifungal potential toward *Magnaporthe grisea* TH16 [68]. Liu et al. isolated **68** from the marine filamentous fungus, *Keissleriella* sp. Y4108, which possessed growth inhibitory activity toward the human fungal pathogens: *Tricophyton rubrum*, *C. albicans*, and *A*. *niger* (MICs 20, 40, and 80 µg/mL, respectively), in comparison to ketoconazole (MICs 10, 1, and 30 µg/mL, respectively) in the broth micro-dilution method [69]. Shushni et al. stated that balticols A–F (**74**, 75, **78**, and **80**–**82**) (Conc. 200 µg/disc) exhibited no remarkable antimicrobial activity toward *S. aureus*, *E*. *coli*, and *C*. *maltosa* in the agar-diffusion assay [70].

**Figure 4:**
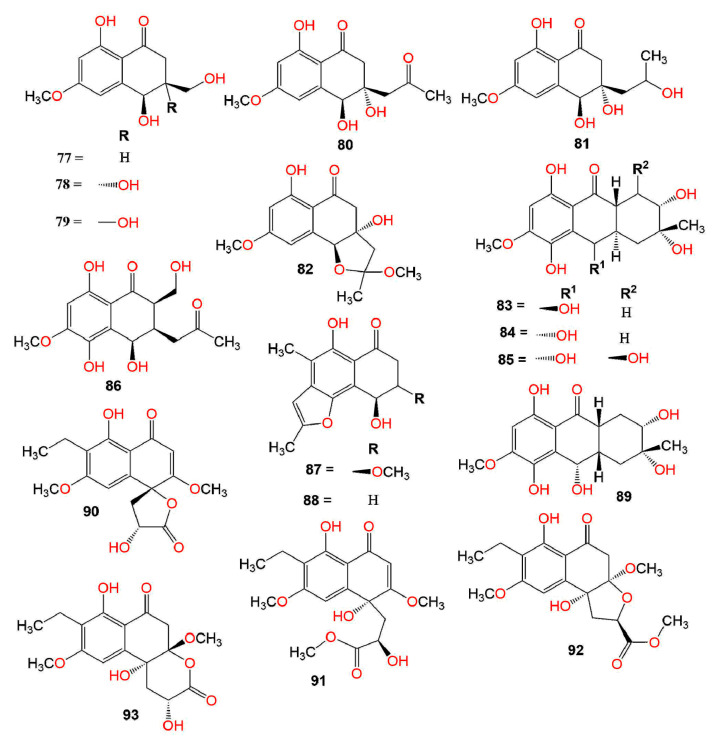
Structures of compounds **77**–**93**.

The five metabolites, **83**–**87** biosynthesized by the sea fan-derived *Fusarium* spp. PSU-F135 and PSU-F14 showed weak antibacterial potential versus *S. aureus* and MRSA in the colorimetric broth micro-dilution test [71] (Figure 4).

Furthermore, **85** exhibited antimalarial potential versus *P. falciparum* K1-MDR strain (IC_50_ 7.94 µg/mL), in comparison to dihydroartimisinin (IC_50_ 0.0012 µg/mL) in the microculture radioisotope technique and antimycobacterial capacity toward *M. tuberculosis* H37Ra (MIC 12.50 µM) in the green fluorescent protein (GFP)-based fluorescent assay, while **84** showed moderate effect (MIC 25.0 µg/mL) toward *M. tuberculosis* H37Ra, compared to kanamycin, rifampicin, and isoniazid (MICs 1.25, 0.047, and 0.25 µg/mL, respectively) [71,72].

Perenniporides A–D (**90**–**93**), new derivatives isolated from the solid culture of *Perenniporia* sp. inhabiting the larva of *Euops chinesis* were characterized by NMR, X-ray, and ECDcalculations. Their configurations were 4R,12R (for **90** and **91**), 3R,4R,12R (for **92**), and 3S,4R,12R (for **93**). Compound **90** was characterized by the presence of a γ-lactone ring that was spirally linked to C-4 of the naphthalen-1(4H)-one moiety and represented the first natural naphthalenone, having a 3′,4′-dihydro-2′H,3H-spiro[furan-2,1′-naphthalen]-5(4H)-one skeleton. However, **91** possessed a C-13 methyl ester instead of the γ-lactone ring in **90**. Both **92** and **93** had fused THF (tetrahydrofuran) ring and δ-lactone ring, respectively, at C-3/C-4 of the 3,4-dihydronaphthalen-1(2H)-one moiety. They represented the first natural metabolites with 1,2,3a,4-tetrahydronaphtho[2,1-b]furan-5(9b*H*)-one and 4a,5,6,10b-tetrahydro-1*H*-benzo[f]chromen-3(2*H*)-one moiety, respectively. Compound **90** exhibited significant antifungal activity toward a five-plant pathogen panel, including *F. moniliforme*, *V. alboatrum*, *G. zeae*, *F. oxysporum*, and *A. longipes* using microplate assay (MIC from 10 to 20 μg/mL), compared to methyl 2-benzimidazolecarbamate that had antifungal potential against all the tested fungi except for *A*. *longipes* (MICs 0.63 to 2.5 μg/mL), whereas **91**–**93** did not have any noticeable activity (Conc. 20 μg/mL) [73].

**Figure 5:**
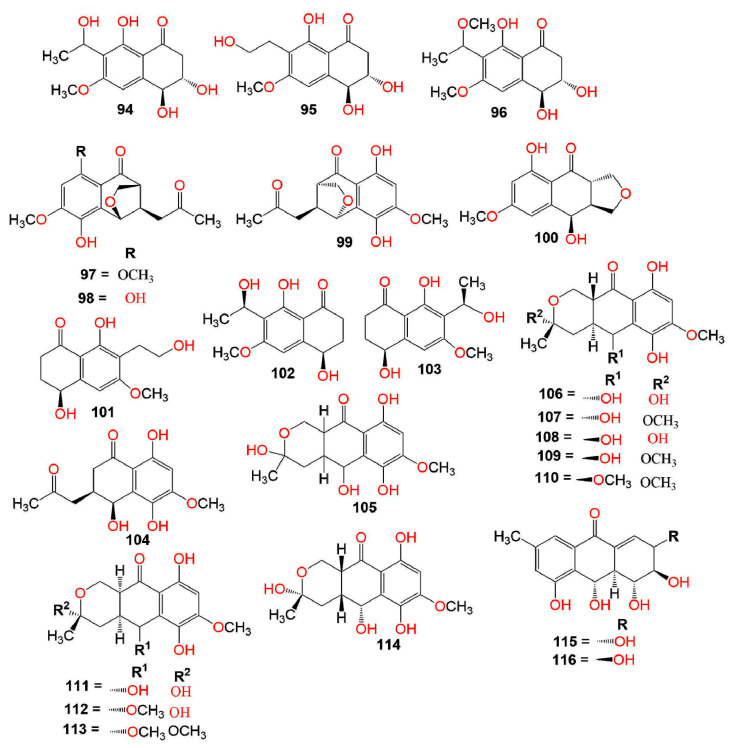
Structures of compounds **94**–**116**.

Also, **53** and **94**–**96** (Conc. 50 µg/mL) had no antimicrobial influence versus *E. coli*, *B. subtilis*, *S. aureus*, *B. pumilus*, *C. albicans*, and *A. niger* in the micro-plate assay [74] (Figure 5). Also, compounds **9**, **40**, **53,** and **94** isolated from *Neofusicoccum parvum* associated with declining grapevines did not show in-vitro antifungal potential versus the plant pathogens; *L. mediterranea*, *D. seriata*, *N. vitifusiforme*, and *P. citrophthora* [46]. Orchid-associated *Daldinia eschscholtzii* produced new derivatives: **45**, **63**, **65**, and **131**, along with the formerly reported **2** and **40**. Compound **131** featured an uncommon oxane-connected binaphthyl ring system that revealed the possible biosynthesis of diverse binaphthyls from ring rearrangements and fusions. Compounds **2**, **63**, **65**, and **131** had moderate antimicrobial potential in the agar diffusion assay toward *B. subtilis*, MRSA, VRE, and *P. notatum*, with **131** showing the broadest and highest activity [21].

**Figure 6:**
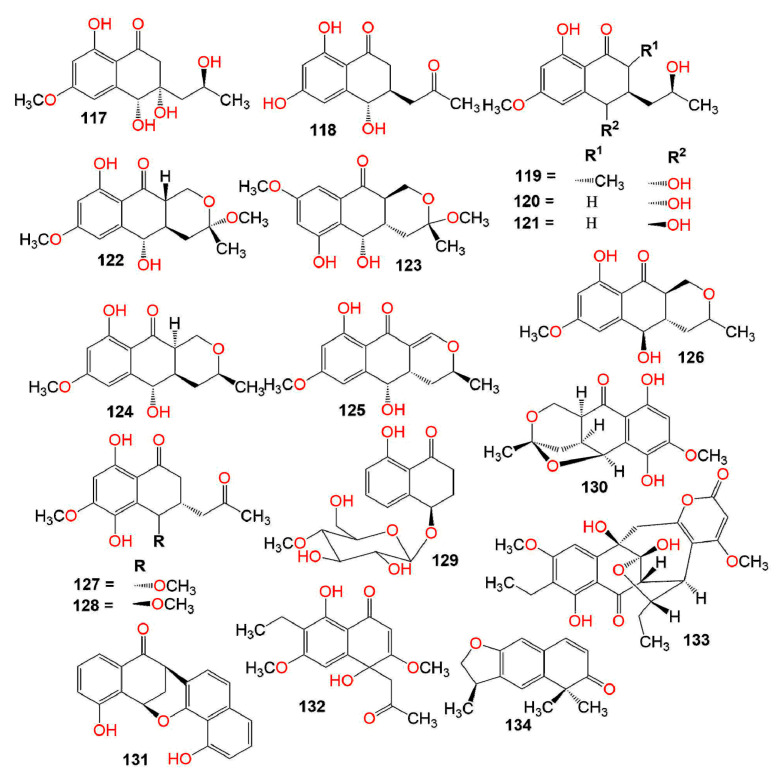
Structures of compounds **117**–**134**.

A study by Kornsakulkarn et al. reported the separation of the new dihydronaphthalenone derivatives: **98**, **99**, **104**, **106**, **107, 111**, and **112** and the known **114** from *Fusarium* sp. BCC14842. Their configuration was assigned using NOESY (nuclear overhauser effect spectroscopy), coupling constants, X-ray, CD (circular dichroism), and modified Mosher method. Only, **99** and **107** showed weak antimycobacterial potential toward *M. tuberculosis* H37Ra (MIC 25.0 and 50.0 µg/mL, respectively), compared to isoniazid (MIC 0.03 µg/mL) in the GFPMA assay. Moreover, none of them had antifungal activity toward *C*. *albicans* (IC_50_ > 50 µg/mL) [75]. In the disc diffusion assay, **115** and **116** had antibacterial effectiveness versus *B. subtilis* (IZD 12.0 and 14.0 mm, respectively) and no activity versus *E. coli* [47]. Variabilone (**134**), with a new dihydrofurano-2(1H)-naphthalenone skeleton was purified from *Paraconiothyrium variabile* obtained from *Cephalotaxus harringtonia* (yew tree) (Figure 6). Its C-11 R-configuration was determined by SRCD (synchrotron radiation circular dichroism). It is noteworthy that **134** had a significant antibacterial activity versus *B. subtilis* (IC_50_ 2.13 μg/mL), compared to kanamycin (IC_50_ 0.36 μg/mL) in the micro-dilution resazurin assay [76]. Cladosporone A (**157**), a new dimeric napthalenone linked via C-C bridge was yielded by *Cladosporium* sp. KcFL6 harboring *Kandelia candel*, together with **145**, **147**, and **148**. They had no antimicrobial effectiveness versus *A. baumannii*, *S. aureus*, *E. faecalis*, *A. hydrophila*, *E. coli*, *K. pneumonia*, *Fusarium* sp., *F*. *oxysporum* f. sp. *cucumeris*, *F.* o*xysporum* f. sp. *niveum*, *A. niger*, and *R. solani* in the disc diffusion assay [77].

Moreover, the antimicrobial potential of **147**, **150**, and **152**–**155** was assessed versus *E. coli*, *A. hydrophila*, *S. aureus*, *E. tarda*, *P. aeruginosa*, *M. luteus*, *V. alginolyticus*, *V. parahemolyticus*, *V. harveyi*, *A. brassicae*, *F. oxysporum*, *C. gloeosporioides*, *G. graminis*, and *P. piricolav* using the micro-plate assay. Compounds **147**, **150**, and **152**–**155** showed inhibitory potential toward *V. harveyi*, *M. luteus*, and *E. coli* (MICs 4–128 μg/mL) [78]. Further, 147 and 149 demonstrated antibacterial capacity versus *S. aureus* (MICs 6.25 and 1.56 μg/mL, respectively), comparing to ciprofloxacin (MIC 0.39 μg/mL) [79]. *Cladosporium* sp. KFD33 separated from blood cockle yielded cladosporol I (**154**) and altertoxin XII (**156**) that displayed quorum sensing inhibition (MICs 30 and 20 µg/well, respectively) in the well diffusion assay toward *Chromobacterium violaceum* CV026 [80].

### 2.3. Cytotoxic Activity

Cancer is a leading cause of death in the world, accounting for ≈10 million deaths in 2020 [81]. Its treatments include radiation therapy, surgical intervention, chemotherapy, or a combination of these options [82]. There are many available therapeutics for treating various types of cancer, however, none of them are totally safe and effective. Many of the reported naphthalenones have been assessed for cytotoxic effectiveness toward various cancer cell lines. Wang et al. separated **1**, **4**, **9**, and **21** from the liquid cultures of *Alternaria* sp. harbored *Morinda officinalis* that showed weak activity (IC_50_ ≥ 200 µM) toward NCI-H460 (human lung carcinoma cell line), MCF-7 (human breast cancer cell line), SF-268 (human glioblastoma cell line), and HepG-2 in the SRB (sulforhodamine B) assay compared with cisplatin [83]. Compounds **8** and **9** obtained from the culture broth of *Penicillium diversum* var. *aureum* inhibited the growth of Yoshida sarcoma cells in tissue culture at concentrations 20–25 µg/mL [84]. Also, El-Amrani et al. separated **1** and **21** from *Aureobasidium pullulans* that did not have anti-proliferative activity toward L5178Y (mouse lymphoma cell line) in the MTT (3-(4, 5-dimethylthiazolyl-2)-2, 5-diphenyltetrazolium bromide) assay [85]. Additionally, **9** was purified from a marine-derived *Aspergillus fumigatus* extract by ODS column chromatography and HPLC. It exhibited notable cytotoxicity (Conc. 60 µM) toward MCF-7 after 24 h incubation. It was found to suppress MMP (mitochondrial membrane potential)-2,-9 expressions via attenuation of the MAPK (mitogen-activated protein kinase) signaling pathway. It also significantly reduced cell mobility and prohibited JNK (c-Jun NH_2_-terminal kinase), ERK (extracellular signal-regulated protein kinase), and P38 (p38 mitogen-activated protein kinase) phosphorylation, involved in cell migration and proliferation. Moreover, it remarkably up-regulated p53 (nuclear transcription factor with a pro-apoptotic function) and down-regulated CDK (cyclin-dependent kinase)4, CDK2, and cyclins (B1 and E). Hence, **9** could be a potential therapeutic for breast cancer [58]. However, it was inactive toward SW-620 and MDA-MB-435 (human melanoma cancer) cell lines in the MTT assay [59]. Compound **17** exhibited moderate cytotoxic potential toward L1210 (mouse lymphocytic leukemia cell line) (IC_50_ 100 µg/mL) and was inactive toward RBL-1 (rat basophilic leukemia cell line), HeLa S3 (human cervix carcinoma cell line), and BHK 21 (fibroblast baby hamster kidney cell line) (IC_50_ > 100 µg/mL) in the microtiter plate assay. On the other hand, **2** was moderately active versus RBL-1, L1210, and BHK 21 (IC_50_ 25–50 µg/mL) and inactive toward the HeLa S3 cell line (IC_50_ > 100 µg/mL) [35].

The cytotoxic activities of **9**, **13**, **14**, **22**, **28**, and **31** toward BCA (human breast cancer), Vero (African green monkey kidney fibroblasts), NCI-H187 (human small cell lung cancer), and KB (human epidermoid carcinoma) were assessed by Pittayakhajonwut et al. using the MTT and SRB methods. Compounds **13** and **22** showed significant cytotoxic potential versus NCI-H187cell line (IC_50_s 2.86 and 5.55 μg/mL, respectively) in comparison to doxorubicin and ellipticine (IC_50_s 0.02 and 0.32 μg/mL, respectively). Additionally, **22** had remarkable cytotoxicity toward BCA (IC_50_ 2.96 μg/mL), compared with ellipticine and doxorubicin (IC_50_s 0.11 and 0.21 μg/mL, respectively). The other compounds were moderately active toward the tested cell lines (IC_50_ ranging from 7.24 to 41.84 μg/mL) [61].

Compounds **18** and **29** were separated from *Phomopsis* sp. sh917 harbored *Isodon eriocalyx* var. *laxiflora* stems. Their absolute configurations 3R for **18** and 3R,4S of **29** were confirmed based on CD, X-ray, or optical rotation comparison. Compound **29** had no obvious inhibitory effect on the viability of HUVECs (human umbilical vascular endothelial cells) (IC_50_ > 100 µM) in the MTT assay [86].

The new naphthalenone derivative **62**, alongside **1**, **9**, and **43** isolated from *Cladosporium* sp. JJM22 accompanied with *C. tagal* had no cytotoxic effect (IC_50_ > 10 μM) in the MTT assay versus HeLa cell line, compared to epirubicin [65]. Compound **48** exhibited weak cytotoxic potential (IC_50_ 25 µg/mL) versus L1210 cells [44]. 

Botryosphaerone E (**55**) purified from *Pyrenochaetopsis* sp. MSX63693 had 3R,4S absolute configuration as confirmed via ECD calculations, using TDDFT (time-dependent density functional theory). It showed weak activity (IC_50_ > 25 µM) versus MDA-MB-435, MDA-MB-231 (human breast cancer cell line), and OVCAR3 (human ovarian cancer cell line) in the MTT assay [87]. The purification of *Paraphoma* sp. extract yielded the new metabolite, 4,6,8-trihydroxy-5-methyl-3,4-dihydronaphthalen-1(2*H*)-one (**59**), along with the known one, **21**. The absolute configuration of **59** was determined as R at C-4 by CD spectra. Both compounds exhibited no cytotoxic influence toward HepG2 (human liver cancer cell line), MCF-7, and Hela (IC_50_ > 40 μM), compared with etoposide (IC_50_s 16.64, 16.11, and 15.00 μM, respectively) in the MTT assay [88]. Compound **63**, isolated from mantis-associated *Daldinia eschscholzii*, had powerful cytotoxic potential in the MTT assay toward mouse splenic lymphocytes (IC_50_ 21.27 µg/mL), in comparison to cyclosporine A (IC_50_ 11.2 µg/mL) [27]. 

Three new dihydronaphthalenones: **66**, **67**, and **69** were separated from *Botryosphaeria* sp. BCC 8200, where **66** and **69** were isolated as two mixtures of inseparable isomers. These metabolites had a weak cytotoxic influence toward MCF-7, NCI-H187, and KB cells and Vero cells in the resazurin and green fluorescent protein micro-plate assays, respectively, compared to ellipticine [68]. 

Xu et al. separated new metabolites, botryosphaerones A–D (**53** and **94**–**96**) from the fermentation culture of *Botryosphaeria australis* strain ZJ12-1A that exhibited no cytotoxic activities versus HepG-2, HeLa, and A549 (lung adenocarcinoma epithelial) cells (Conc. of 10 µg/mL) in the MTT assay [74]. On the other hand, **98**, **99**, **104**, **106**, **107, 111**, **112**, and **114** exhibited weak to moderate cytotoxic activity toward NCI-H187, MCF-7, Vero, and KB cell lines (IC_50_ 5.38–31.69 µg/mL) in comparison to doxorubicin and ellipticine [75]. 

Two new derivatives; aspvanicin A (**127**) and its epimer aspvanicin B (**128**) were obtained from EtOAc extract of the co-culture of the endophytic fungus *Aspergillus versicolor* KU258497 with *B. subtilis*. Their configurations (3S,4S)-**127** and (3S,4R)-**128** were assigned by NMR and ECD analysis assisted by DFT conformational analysis and TDDFT-ECD calculations. Compound **128** had moderate cytotoxic potential toward L5178Y (IC_50_ 22.8 µM), compared to kahalalide F (IC_50_ 4.3 µM) in the MTT assay [89]. 

A novel dihydronaphthalenone, phomonaphthalenone A (**130**) with unusual tetrahydropyran and tetrahydrofuran moieties at C-4a/C-10a and C-3/C-5a, respectively, was purified from *Phomopsis* sp. HCCB04730 solid cultures. Its 3R, 5S, and 10aS configuration was assigned based on the coupling constant and NOESY in combination with X-ray and CD analyses. It exhibited significant cytotoxicity versus A549, MDA-MB-231, and PANC-1 (human Pancreatic cancer cell line) (IC_50_s 92.5, 64.2, and 52.7 µg/mL, respectively), compared to 5-fluorouracil (IC_50_s 75.0, 47.0, and 65.0 µg/mL, respectively) in the MTT assay [90]. Compound **131** possessed weak anti-proliferative potential toward HUVEC (GI_50_ 98.4 µM) and K-562 (human leukemia cell line) (GI_50_ 85.5 µM) cell lines [21].

**Figure 7:**
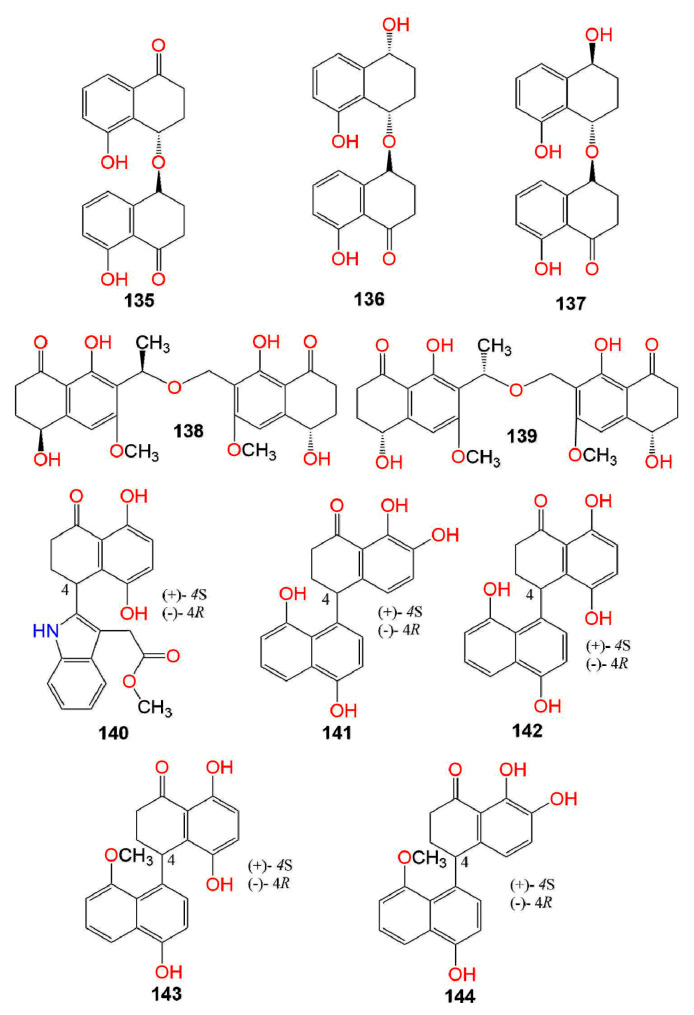
Structures of compounds **135**–**144**.

Delitschiapyrone A (**133**), an α-pyrone-naphthalenone derivative, having a new 6/6/7/5/6-fused ring system was purified from the solid culture of *Delitschia* sp. FL1581, inhabiting *Serenoa repens* leaves. It was characterized by spectroscopic data, X-ray, and ECD that proved the existence of a unique pentacyclic skeleton in which α-pyrone and naphthalenone moieties were connected by a seven-membered carbocyclic ring. This compound showed cytotoxic activity toward MCF-7, H460, HepG2, and U2OS (human osteosarcoma cell line) (IC_50_s 35.5, 12.9, 12.3, and 20.4 μM, respectively), using the Alamar Blue assay compared to cisplatin (IC_50_S 9.4, 2.2, 8.3, and 6.4 µM, respectively) [28]. Compounds **57**, **58**, **101**–**103**, **138**, and **139** displayed no cytotoxicity versus A549, HeLa, and MCF-7 in the MTT assay (conc. 50 µM) [91] (Figure 7). Compound **147** and **157** (IC_50_s ranging from 11.4 to 72.5 µM for **147** and from 10.1 to 53.7 µM for **157**) had moderate cytotoxic potential toward K562, Huh-7, MCF-7, HL-60 (human promyelocytic leukemia cell line), U937, H1975, MOLT-4, BGC823, A549, and HeLa cell lines, compared to trichostatin A [77]. 

Zurlo et al. stated that **145** had marked anti-proliferative capacity versus HT-29, SW480, and CaCo-2, particularly versus HT-29. They indicated that the HT-29 cells’ exposure to **145** led to G1/S phase cell cycle arrest, aided by a powerful p21^waf1/cip1^ expression, a remarkable down-regulation of CDK2, CDK4, and cyclins E and D1, and repression of CDK2 and CDK4 kinase activity [92]. This anti-proliferative potential toward HT-29 cells was induced through activating PPARγ, leading to p21^waf1/cip1^ expression up-regulation and inducing β-catenin degradation, as well as impairment of TCF/β-catenin pathway as evident by reduced cyclin D1 and c-Myc transcription. Finally, it induced the expression of E-cadherin, therefore, antagonizing invasion and metastasis [93]. Moreover, Koul et al. studied the cytotoxic potential of **145** separated from *C. cladosporioides* isolated from *Datura innoxia* toward MCF-7 cell lines. It was found that **145** induced microtubules depolymerization and sensitized programmed cell death through ROS-mediated autophagic flux, resulting in mitophagic cell death [94].

**Figure 8:**
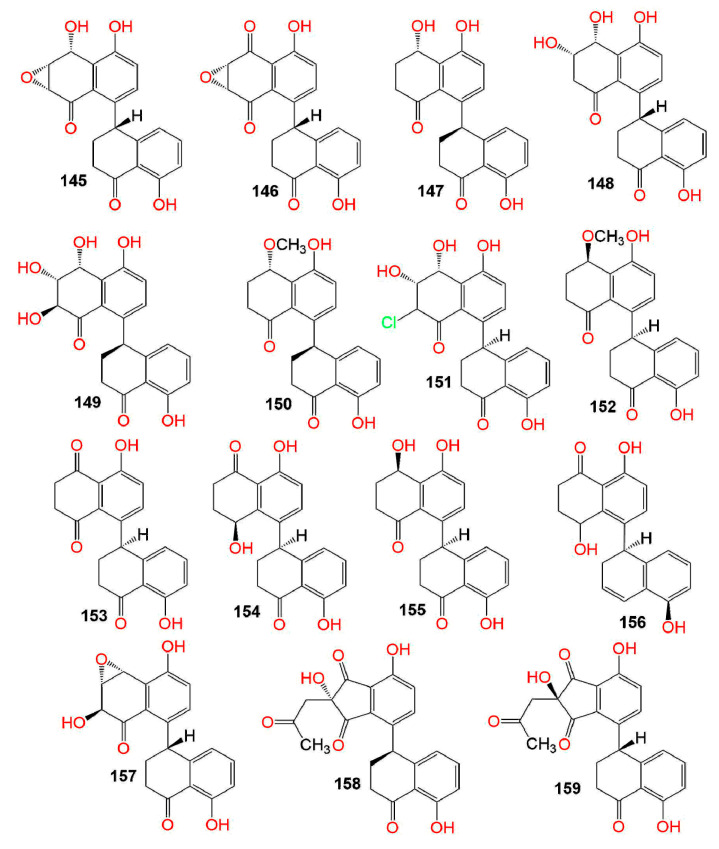
Structures of compounds **145**–**159**.

Marine sediments-derived *C*. *cladosporioides* HDN14-342 yielded **147** and **149**–**151** (Figure 8). Compounds **150** and **151** exhibited cytotoxic potential versus HCT-116, K562, and HeLa cell lines (IC_50_s 3.9–23.0 µM) in comparison to doxorubicin (IC_50_s 0.2–0.8 µM), whereas the other metabolites were inactive (IC_50_ > 50.0 µM) [95]. Li et al. purified six derivatives; **147**, **150**, and **152**–**155** from *C*. *cladosporioides* EN-399 and assessed their activities versus L02, H446, HeLa, A549, Huh7, SW1990, LM3, and MCF-7 in the MTT assay. It is noteworthy that **147**, **152**, and **153** had cytotoxic potential (IC_50_s ranging from 1.0 to 20.0 μM) toward most of the cell lines. Notably, **153** displayed cytotoxic capacity toward Huh7, A549, and LM3 cell lines (IC_50_s 1.0, 5.0, and 4.1 μM, respectively), compared to fluorouracil (IC_50_ 6.2 μM for Huh7) and cisplatin (IC_50_ 9.1 μM for LM3 and 1.3 μM for A549), whereas **147** showed marked cytotoxic effect (IC_50_ 4.0 μM) versus H446 similar to adriamycin (IC_50_ 4.0 μM). These results indicated the dihydro-1,4-naphthoquinone nucleus was substantial for the activity (**153** versus **150**, **152**, and **147**, **154**, and **155**) and C-4 methoxyl intensified the effect (**152** versus **155**) [78]. Nevertheless, **154** (Conc. 100 μM) showed no noticeable activity versus HepG-2, SF-268, MCF-7, and NCI-H460 in the SRB assay [96]. 

### 2.4. Antioxidant Activity

Compounds **9** and **11** separated from *Xylariaceous* PSU-A80 were assessed for their antioxidant activity using the DPPH (1,1-diphenyl-2-picrylhydrazyl) assay. Compound **11** trapped DPPH radical with 2.65% scavenging activity (Conc. 50 µg/mL), whereas its methylated derivative **11** exhibited better activity than **9** [97]. Whilst **49** and **50** obtained from *A. roseogriseum* associated with the brown alga, *Cladostephus spongius*, exhibited weak activity with percentage radical scavenging of 2.8 and 12.1 at concentrations 100 and 500 µg/mL, respectively, in the DPPH assay [98].

### 2.5. Serotonin Antagonistic Activity

Serotonin (5-HT) is a neurotransmitter in the central and peripheral nervous systems. It has been implicated in the etiology of various physio-pathological disorders such as anxiety, depression, schizophrenia, social phobia, IOP (intraocular pressure) modification, migraine, systemic and pulmonary hypertension, irritable bowel syndrome, vomiting, and eating disorders [99,100]. 5-HT_2C_ antagonists have been considered as a potential target for treating various health disorders [101]. Bös et al. reported the isolation of the first nitrogen-free 5-HT ligands; **46** and **47** from *Aspergillus parvulus* and assessed their binding affinity for 5-HT_2c_ and 5-HT_2A_ human receptors, using displacement of [^3^H]-DOB and [^3^H]-5-HT, respectively. It is noteworthy that they are preferably bound to the 5-HT2c receptor and displayed antagonistic capacities with p*K*i values 6.7 and 6.4, respectively. On the other hand, they could not displace [^3^H]-DOB from the 5-HT_2A_ receptor binding site at Conc. up to 10 mM. It was found that the alkyl side chain was essential for binding, however, the phenolic OH was not implicated in binding to the receptor [102].

### 2.6. Antiviral Activity

The new naphthalenone derivatives; **74**, **75**, **78**, and **80**–**82** were assessed for their antiviral activity toward Herpes simplex virus type I (HSV-1, strain KOS) and influenza virus A/WSN/33 (H1N1) at non-cytotoxic concentrations by a dye-uptake assay using neutral red. A remarkable activity for balticols D–F (**80**–**82**) was noticed toward HSV-1 (IC_50_s 0.1, 0.01, and 0.1 µg/mL, respectively), compared to aciclovir (IC50 0.1 µg/mL). The other balticols A–C had moderate activity against HSV-1 (IC_50_ 1.0 µg/mL). Whilst **75**, **78**, **80**, and **82** exhibited activities toward H1N1 (IC_50_s 10.0, 1.0, 0.1, 1.0 µg/mL, respectively), compared to amantadine sulphate (IC_50_ 15.0 µg/mL) [70]. Moreover, **130** displayed a significant HIV-1 inhibitory potential (IC_50_ 11.6 µg/mL) in the luciferase assay system using 293T cells, compared with efavirenz (IC_50_ 4.7 × 10^−4^ µg/mL) [90].

### 2.7. Melanin Synthesis Inhibitory Activity 

Melanins are high molecular weight black or dark brown pigments commonly found in microorganisms, plants, and animals that are produced by oxidative polymerization [24]. They are not required for development and growth, but they enhance the competitiveness and survival of these species under conditions of electromagnetic and UV irradiation, high temperature, and desiccation [103]. Most fungal melanins are derived from DHN (1,8-dihydroxynaphthalene) [24]. These pigments are correlated with the enhanced virulence of parasitic fungi and play a remarkable role in fungal pathogenic infections [104]. The newly isolated scytalols A–D (**70**–**73**) from the mycelial culture of *Scytalidium* sp. obtained from Basidiomycete infected body, growing on wood were assessed for their inhibitory effect on DHN (1,8-dihydroxynaphthalene) melanin biosynthesis using *Lachnellula* sp. A32-89 in the agar cultures. Only **70** and **73** selectively inhibited DHN melanin synthesis, however, **70** and **71** were inactive [105].

### 2.8. Enzymes Inhibitory Activity

The phytotoxic metabolite **3**, which is an important intermediate in fungal melanin biosynthesis, was purified from *Xylariaceae* sp. SCSGAF0086 culture broth. It was found to show enzyme inhibitory potential toward PTPlB (protein-tyrosine phosphatase 1B), SHP2 (Src-homology 2 domain-containing phosphotyrosine phosphatase), and IMPDH (inosine monophosphate dehydrogenase) (IC_50_s 13.9, 4.1, and 41.2 μM, respectively), compared with mycophenolic acid (IC_50_ 0.4 μM for IMPDH) and ursolic acid (IC_50_s 2.8 μM for PTPlB and SHP2). It is noteworthy that SHP2 is a target for anti-tumor agent screening and IMPDH and PTPlB are targets for screening immuno-suppressive and anti-diabetic agents, respectively. Therefore, **3** was a PTPlB and SHP2 inhibitor [106]. Wang et al. reported that **1** and **4** exhibited remarkable α-glucosidase inhibitory potential compared to (IC_50_s 34.88 and 102.34 µM, respectively) acarbose (IC_50_ 427.34 µM) in the colorimetric assay, which could be beneficial for developing α-glucosidase inhibitors [82]. 

IDO (indoleamine 2,3-dioxygenase) controls the rate-limiting steps in tryptophan (Trp) metabolism that is correlated with various disorders such as Parkinson’s and Alzheimer’s diseases and cataracts. Hence, it is considered a promising target for tumor immunotherapy [107]. Cui et al. separated and characterized a new metabolite; **54**, together with **47**, **53**, and **95** from the CH_2_Cl_2_ extract of *Neofusicoccum austral* SYSU-SKS024 obtained from *Kandelia candel* fresh branch. Compound **54** was like **53** with an extra methoxy group and had 3R,4R configuration as evident by the ECD spectrum. Compounds **47**, **53**, and **95** (Conc. 40 μM) showed no IDO (indoleamine 2,3-dioxygenase) inhibitory activity, whereas **54** had relatively significant inhibitory potential (IC_50_ 6.36 μM), compared with epacadostat (IC_50_ 0.5 μM) in the fluorescence-based assay [107]. Xiao et al. reported the separation of a new derivative, (2*S*,3*S*,4*S*)-8-dehydroxy-8-methoxyl-dihydronaphthalenone (**97**) and the formerly separated **88** and **98** from *Fusarium* sp. HP-2 isolated from “Qi-Nan” agarwood. Compound **97** had the same structure as **98**, which was previously reported as a new metabolite, except for the existence of an additional methoxy group at C-8 [75]. Its configuration was assigned as 2S,3S,4S based on the negative specific rotation as in **98**. Only, **97** had a weak AChE (acetylcholinesterase) inhibitory activity (inhibition ratio 11.9%, Conc. 50 μM/mL) [108].

### 2.9. Anti-Inflammatory Activity

Inflammation is a host defense mechanism, which enables the body to survive during injury or infection and maintains the homeostasis of tissues in noxious conditions [109].

Endogenous nitric oxide (NO) plays a critical role in maintaining the homeostasis of varied cellular functions. NO local concentrations are highly dynamic as their synthesis is regulated by independent enzymatic pathways. NO has been shown to have a modulatory effect on inflammation, decreasing the secretion of pro-inflammatory cytokines in human alveolar macrophages challenged with bacterial lipopolysaccharides (LPS), while not altering the basal cytokine levels. Drugs used for managing inflammatory disorders relieve these ailments, but they may have serious life-threatening consequences [110,111,112]. Therefore, there is great enthusiasm for developing novel, safe therapeutics from natural sources for the treatment of inflammation. The reported studies revealed that the anti-inflammatory potential of thiophenes could be due to the inhibition of the activation of the NF-κB (nuclear factor-κB) pathway that regulates the expression of pro-inflammatory cytokines and chemokines [111]. A new β-tetralonyl glucoside; **129** was purified from the culture of *Colletotrichum* sp. GDMU-1 associated with *Santalum album* leaves. Compound **129** had 4′-methyl *β*-glucopyranose moiety linked at C-4. Enzymatic hydrolysis followed by ECD spectrum for the hydrolysis product sclerone (**8**) revealed an R-configuration at C-4. It possessed no inhibitory capacity on NO (nitric oxide) production elicited by LPS (lipopolysaccharide) in RAW264.7 cells [112]. The new metabolites; **57**, **58**, **101**–**103**, **138**, and **139** were purified from the marine-derived fungus *Leptosphaerulina chartarum* 3608. Both **102**/**103** and **57**/**101** were enantiomers as they had opposite optical rotations and Cotton effects in their CD spectra. Their configuration was assigned based on ECD. Compounds **58** and **138** were dimeric naphthalenones, consisting of two monomeric units; leptothalenone A (**102**/**103**) and 10-norparvulenone (**58**). Unfortunately, only **139** possessed moderate inhibitory potential on the LPS-induced NO production (IC_50_ 44.5 µM) in the RAW264.7 cells using the Griess assay, compared to indomethacin (IC_50_ 37.5 µM), however, the other metabolites (IC_50_ > 100 µM) had no significant anti-inflammatory activity [91]. In the anti-COX-2 assay, **148** and **157** revealed COX-2 inhibition (IC_50_s 60.2 and 49.1 μM, respectively) upon comparison to indomethacin and NS-398 [77].

### 2.10. Neuroprotective Activity

Girich et al. studied the neuro-protective potential of **2** and **32** purified from the broth culture of *Penicillium* sp. KMM 4672 associated with *Padina* sp. (brown alga) in 6-OHDA (6-hydroxydopamine), paraquat, and rotenone-induced Parkinson’s disease in Neuro-2a cells. They have been found to significantly increase the viability of paraquat- and rotenone-treated Neuro-2a cells and decreased the elevated ROS level induced by these neurotoxins [113]. Five pairs of undescribed enantiomers, xylarinaps A–E (**140**–**144**), including a pair of indole naphthalenones (**140**) and four pairs of naphthalenone-naphthalene dimers (**141**–**144**) were separated from the EtOAc of *Xylaria nigripes*. Their neuroprotective effects toward the OGD (oxygen and glucose deprivation)-induced damage to PC12 cells in the CCK-8 (cell counting kit-8) assay were assessed. They significantly promoted cell viability, decreased MDA (malondialdehyde) levels, and increased the SOD (superoxide dismutase) and GSHPx (glutathione peroxidase) levels, as well as further notably prohibited apoptosis, which could be the mode of their neuroprotective effect [29].

### 2.11. Other Activities

Corynenones A and B (**51** and **52**); new derivatives separated from *Corynespora cassiicola* XS-20090I7 were diastereoisomers, differing in the 3,4-diol centers’ configuration; 3S,4S for **51** and 3S,4R for **52** based on the ECD spectra and spectroscopic data. They had no antifouling potential toward the barnacle larval settlement using cyprids of *Balanus amphitrite* [114]. Compound **63** possessed a weak immunosuppressive effect in the T-cell viability assay (IC_50_ > 10.0 µg/mL), in comparison to cyclosporine A (IC_50_ 0.06 µg/mL) [89]. Moreover, in 2020, He et al. reported that **147** (IC_50_ >200 µM) exhibited no anti-allergic potential toward RBL-2H3 cells, compared to loratadine (IC_50_ 35.01 µM) [115]. 

## 3. Conclusions

**Figure 9:**
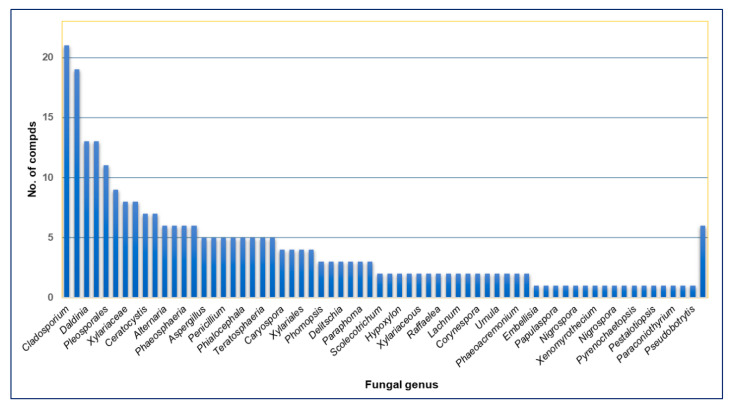
Numbers of naphthalenone derivatives reported from different fungal genera.

In recent years, more metabolites have been discovered from fungi derived from diversified sources such as plants, animals, soil, and marine. The current review describes the naphthalenone derivatives reported from fungi, focusing on their biosynthesis and bioactivities. In fact, the available literature analysis revealed that a total of **159** naphthalenones with diverse chemical structures and various bioactivities were reported from 66 identified fungal genera and three unidentified genera. The largest number of derivatives have been obtained from *Fusarium*, *Cladosporium*, *Daldinia*, *Biatriospora*, *Neofusicoccum*, *Leptosphaerulina*, and *Xylariaceae* (Figure 9). 

**Figure 10:**
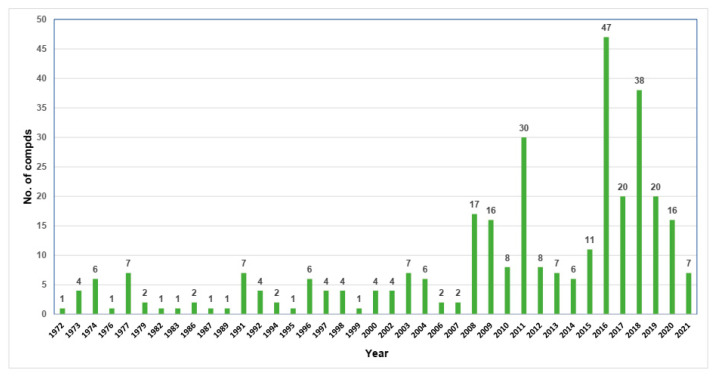
Numbers of naphthalenone derivatives reported by year.

Compounds **1**, **3**, **8**, **9**, and **17** are the most commonly reported derivatives from various fungal genera. The majority of naphthalenones have been reported in the period from 2008 to 2020 (Figure 10). 

This was due in part to limited knowledge of the fungal diversity and isolation and cultivation techniques that resulted in many fungal species remaining undiscovered [116]. The observed increase in the number of isolated derivatives can be attributed to the extensive application of new separation, screening, and characterization techniques. Also, the advances in the fungal cultivation strategies, as well as the wide array of genetic tools now available, allow for the fungal genome and metabolome to be readily exploited, leading to enhanced discovery of the value-added metabolites [117].

Many of the reported naphthalenones have remarkable phytotoxic potential. As such, they can be used as bioherbicides or as lead compounds for the synthesis of more efficacious phytotoxic compounds capable of targeting a wide range of weeds. 

**Figure 11:**
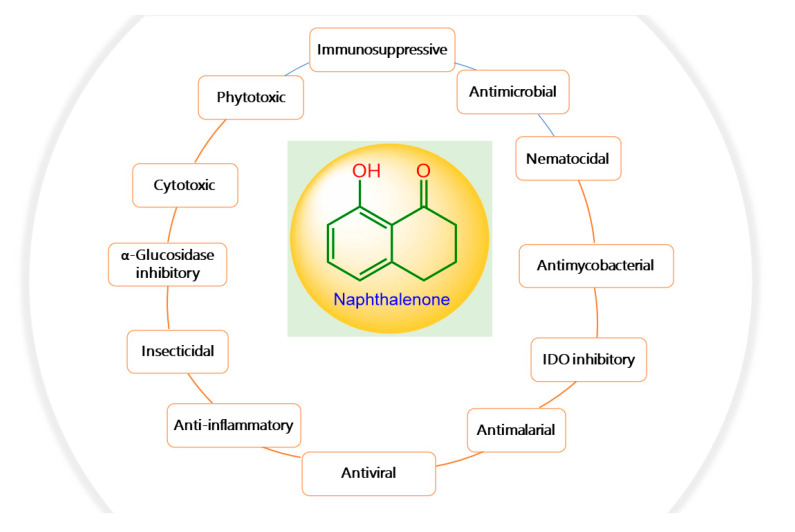
Promising biological activities of naphthalenone derivatives.

These metabolites have been assessed for antimicrobial, antimycobacterial, cytotoxic, nematocidal, antioxidant, serotonin antagonistic, antiviral, anti-inflammatory, neuroprotective, and anti-plasmodial, as well as melanin synthesis and enzyme inhibitory potential (Figure 11). 

Some metabolites have shown promising activities that could be utilized as building blocks for the synthesis of various compounds for treating diverse human disorders. However, these metabolites remain to be further in-vivo tested for their bioactivities. Reports on naphthalenones indicated that differences in structural characteristics of derivatives often correlated with different bioactivities. For example, an increasing number of hydroxyl groups attached to the naphthalenone skeleton enhanced phytotoxic activity. It is noteworthy that compound **90** with a spirally linked γ-lactone ring at C-4 of the naphthalen-1(4H)-one moiety possessed potent antifungal potential than **91**, **92,** and **93** that have C-13 methyl ester, fused THF ring, and δ-lactone ring, respectively. Also, **131** with an oxane-connected binaphthyl ring system had a more powerful antimicrobial capacity than **63** and **65**, which have C6-C7 fused furan rings. In the dimeric napthalenones, the C-4 α-configured hydroxyl group was found to be essential for antimicrobial activity as in 147 and 149, however, its replacement with carbonyl (e.g., **153** and **154**), methoxy group (**150** and **152**), or β-configured hydroxyl group reduced the activity (e.g., **155**). In the cytotoxicity results, compound **13** with the C-3 methoxy group exhibited higher activity than its non-methoxylated one (**22**). Additionally, it was revealed that the dihydro-1,4-naphthoquinone nucleus was substantial for the cytotoxic activity (e.g., **153** vs. **150**, **152**, and **147**, **154**, and **155**) and the C-4 methoxy group intensified the effect (**152** vs. **155**). 

On the other hand, there are limited or no studies that focus on the mechanism of action of these metabolites. In addition, many of the reported metabolites have not been evaluated for their bioactivities, this may be due to either the lack of assays or not enough amount of the isolated compounds to perform these assays. Many of the tested metabolites had no substantial effectiveness in some evaluated bioactivities. Finally, assessment of other potential activities and derivatization of these compounds, as well as in-vivo and mechanistic studies of the active compounds should undoubtedly be the focus of future research.

## Data Availability

Not applicable.

## Supplementary Materials

The following supporting information can be downloaded at: https://www.mdpi.com/article/10.3390/toxins14020154/s1, Table S1: List of fungal naphthalenones (Fungal source, host, and place), Table S2: Biological activities of fungal naphthalenones. References [118,119,120,121,122,123,124,125,126,127,128,129,130,131,132,133,134,135,136,137,138,139,140,141,142,143,144,145,146,147,148,149,150,151,152,153,154,155,156,157,158,159,160,161,162,163,164,165,166,167] are cited in the supplementary materials.

## Abbreviations

A549: Lung adenocarcinoma epithelial cell line; AChE: Acetylcholinesterase; B16 F-1: Mouse melanoma, producing melanin; BC: Human breast cancer; BCA: Human breast cancer; BEL-7402: Human hepatocellular carcinoma cell line; BHK 21: Fibroblast baby hamster kidney cell line; CC_50_: 50% cytotoxic concentration; CCK8: Cell Counting Kit-8; CDK: Cyclin-dependent kinase; DHN: 1,8-Dihydroxynaphthalene; DHN: 1,8-Dihydroxynaphthalene; DPPH: 1,1-Diphenyl-2-picrylhydrazyl**;** ECD: Electronic circular dichroism; ERK: Extracellular signal-regulated protein kinase; GFPMA: Green fluorescent protein microplate assay; GI_50_: Concentration for 50% of maximal inhibition of cell proliferation; GSHPx: Glutathione peroxidase; H460: Human lung carcinoma cell line; HeLa S_3_: Human cervix carcinoma cell line; HepG2: Human liver cancer cell line; HL-60: Human promyelocytic leukemia cell lines; HUVECs: Human umbilical vascular endothelial cells; IDO: Indoleamine 2,3-dioxygenase; IMPDH: Inosine monophosphate dehydrogenase; IZD: Inhibition zone diameter; JNK: c-Jun NH2-terminal kinase; K-562: Human leukemia cell lines; K-562: Human chronic myeloid cell lines; KB: Human epidermoid carcinoma, ATCC CCL-17; KB: Oral cavity cancer; L1210: Mouse lymphocytic leukemia cell line; L5178Y: Mouse lymphoma cell lines; LC_50_: Lethal concentration 50; MABA: Microplate Alamar blue assay; MAPK: Mitogen-activated protein kinases; MCF-7: Human breast cancer cell lines; MDA: Malondialdehyde; MDA-MB-231: Human breast cancer cell lines; MDA-MB-435: Human melanoma cancer cell lines; MMP: Mitochondrial membrane potential; mRNA: Messenger ribonucleic acid; MSH: Melanin stimulation hormone; MTT: 3-(4,5-Dimethylthiazol-2-yl)-2,5-diphenyltetrazolium bromide; NCI-H187: Human small cell lung cancer; NCI-H460: Human lung carcinoma cell line; NO: Nitric oxide; OGD: Oxygen and glucose deprivation; OVCAR3: Human ovarian cancer cell lines; P38: p38 mitogen-activated protein kinases; P53: Nuclear transcription factor with a pro-apoptotic function; PANC-1: Human Pancreatic cancer cell lines; PTPlB: Protein tyrosine phosphatase 1B; RBL-1:Rat basophilic leukemia cell line; SF-268: Human glioblastoma cell line; SGC-7901: Human gastric cancer cell line; SHP2: Src homology 2 domain-containing phosphotyrosine phosphatase; SOD: Superoxide dismutase; SRB: Sulforhodamine B; SRCD: Synchrotron radiation circular dichroism; T3HN: 1,3-8-trihydroxy naphthalene; T4HN: 1,3,6,8-Tetrahydroxynaphthalene; TDDFT: time-dependent density functional theory; U2OS: Human osteosarcoma cell line; VEGF: Vascular endothelial growth factor; Vero: African green monkey kidney fibroblasts; XTT: Cell proliferation kit II.

## References

[1] Hawksworth D.L., Lücking R. (2017). Fungal diversity revisited: 2.2 to 3.8 million species. Microbiol. Spectr..

[2] Ibrahim S.R.M., Altyar A.E., Mohamed S.G.A., Mohamed G.A. (2021). Genus Thielavia: Phytochemicals, industrial importance and biological relevance. Nat. Prod. Res..

[3] Ibrahim S.R.M., Mohamed S.G.A., Sindi I.A., Mohamed G.A. (2021). Biologically active secondary metabolites and biotechnological applications of species of the family Chaetomiaceae (Sordariales): An updated review from 2016 to 2021. Mycol. Prog..

[4] Ibrahim S.R.M., Mohamed S.G.A., Altyar A.E., Mohamed G.A. (2021). Natural Products of the Fungal Genus Humicola: Diversity, Biological Activity, and Industrial Importance. Curr. Microbiol..

[5] Ibrahim S.R.M., Sirwi A., Eid B.G., Mohamed S.G.A., Mohamed G.A. (2021). Bright Side of *Fusarium oxysporum*: Secondary Metabolites Bioactivities and Industrial Relevance in Biotechnology and Nanotechnology. J. Fungi.

[6] Ibrahim S.R., Mohamed G.A., Kamal H.M., Mohamed S.G., Khedr A.I. (2022). Terretonins from Aspergillus Genus: Structures, Biosynthesis, Bioactivities, and Structural Elucidation. Mini-Rev. Org. Chem..

[7] Mohamed G.A., Ibrahim S.R.M. (2021). Untapped potential of marine-associated Cladosporium species: An overview on secondary metabolites, biotechnological relevance, and biological activities. Mar. Drugs.

[8] Srivastava A.K. (2019). The role of fungus in bioactive compound production and nanotechnology. Role of Plant Growth Promoting Microorganisms in Sustainable Agriculture and Nanotechnology.

[9] Ibrahim S.R., Mohamed G.A. (2015). Naphthylisoquinoline alkaloids potential drug leads. Fitoterapia.

[10] Mohamed G.A., Ibrahim S.R.M., El Agamy D.S., Elsaed W.M., Sirwi A., Asfour H.Z., Koshak A.E., Elhady S.S. (2021). Terretonin As A new protective agent against sepsis-induced qcute lung injury: Impact on SIRT1/Nrf2/NF-κBp65/NLRP3 signaling. Biology.

[11] Ibrahim S.R.M., Elkhayat E., Mohamed G.A.A., Fat’Hi S.M., Ross S.A. (2016). Fusarithioamide A, a new antimicrobial and cytotoxic benzamide derivative from the endophytic fungus Fusarium chlamydosporium. Biochem. Biophys. Res. Commun..

[12] Ibrahim P.S.R., Mohamed G., Ahmed H. (2016). Aegyoxepane: A New Oxepane Derivative from the Fungus Aspergillus aegyptiacus. Lett. Org. Chem..

[13] Ibrahim S.R.M., Mohamed G.A. (2016). Naturally occurring naphthalenes: Chemistry, biosynthesis, structural elucidation, and biological activities. Phytochem. Rev..

[14] Ibrahim P.S.R., Mohamed G.A., Ross S. (2016). Integracides F and G: New tetracyclic triterpenoids from the endophytic fungus *Fusarium* sp.. Phytochem. Lett..

[15] Ibrahim S.R., Abdallah H.M., Mohamed G.A., Ross S.A. (2016). Integracides H-J: New tetracyclic triterpenoids from the endophytic fungus *Fusarium* sp.. Fitoterapia.

[16] Ibrahim S.R.M., Mohamed G.A., Khedr A.M.I. (2017). γ-Butyrolactones from Aspergillus species: Structures, biosynthesis, and biological activities. Nat. Prod. Commun..

[17] Ibrahim S.R., Mohamed G.A., Al Haidari R.A., El-Kholy A.A., Zayed M.F., Khayat M.T. (2018). Biologically active fungal depsidones: Chemistry, biosynthesis, structural characterization, and bioactivities. Fitoterapia.

[18] Ibrahim S.R., Mohamed G.A., Al Haidari R., Zayed M., El-Kholy A.A., Elkhayat E., Ross S.A. (2018). Fusarithioamide B, a new benzamide derivative from the endophytic fungus Fusarium chlamydosporium with potent cytotoxic and antimicrobial activities. Bioorg. Med. Chem..

[19] Ibrahim P.S.R., Abdallah H., Elkhayat E., Al Musayeib N.M., Asfour H.Z., Zayed M., Mohamed G.A. (2017). Fusaripeptide A: New antifungal and anti-malarial cyclodepsipeptide from the endophytic fungus *Fusarium* sp.. J. Asian Nat. Prod. Res..

[20] Ibrahim S.R.M., Sirwi A., Eid B.G., Mohamed S.G.A., Mohamed G.A. (2021). Fungal depsides naturally inspiring molecules: Biosynthesis, structural characterization, and biological activities. Metabolites.

[21] Barnes E.C., Jumpathong J., Lumyong S., Voigt P.-D.D.K., Hertweck C. (2016). Daldionin, an Unprecedented Binaphthyl Derivative, and Diverse Polyketide Congeners from a Fungal Orchid Endophyte. Chem. A Eur. J..

[22] Xu D., Xue M., Shen Z., Jia X., Hou X., Lai D., Zhou L. (2021). Phytotoxic Secondary Metabolites from Fungi. Toxins.

[23] Andolfi A., Mugnai L., Luque J., Surico G., Cimmino A., Evidente A. (2011). Phytotoxins Produced by Fungi Associated with Grapevine Trunk Diseases. Toxins.

[24] Langfelder K., Streibel M., Jahn B., Haase G., Brakhage A.A. (2003). Biosynthesis of fungal melanins and their importance for human pathogenic fungi. Fungal Genet. Biol..

[25] Watanabe A., Fujii I., Tsai H., Chang Y.C., Kwon-Chung K.J., Ebizuka Y. (2000). Aspergillus fumigatus alb1 encodes naphthopyrone synthase when expressed in Aspergillus oryzae. FEMS Microbiol. Lett..

[26] Wheeler M.H., Stipanovic R.D. (1985). Melanin biosynthesis and the metabolism of flaviolin and 2-hydroxyjuglone inWangiella dermatitidis. Arch. Microbiol..

[27] Zhang Y.L., Zhang J., Jiang N., Lu Y.H., Wang L., Xu S.H., Wang W., Zhang G.F., Xu Q., Ge H.M. (2011). Immunosuppressive Polyketides from Mantis-AssociatedDaldinia eschscholzii. J. Am. Chem. Soc..

[28] Luo J.G., Wang X.B., Xu Y.M., U’Ren J.M., Arnold A.E., Kong L.Y., Gunatilaka A.A. (2014). Delitschiapyrone A, a pyrone-naphthalenone adduct bearing a new pentacyclic ring system from the leaf-associated fungus Delitschia sp. FL1581. Org. Lett..

[29] Li J., Li L.-Q., Long H.-P., Liu J., Jiang Y.-P., Xue Y., Wang W.-X., Tan G.-S., Gong Z.-C., Liu J.-K. (2021). Xylarinaps A–E, five pairs of naphthalenone derivatives with neuroprotective activities from Xylaria nigripes. Phytochemistry.

[30] Fernández-Aparicio M., Delavault P., Timko M.P. (2020). Management of Infection by Parasitic Weeds: A Review. Plants.

[31] Macías-Rubalcava M.L., Garrido-Santos M.Y. (2022). Phytotoxic compounds from endophytic fungi. Appl. Microbiol. Biotechnol..

[32] Iwasaki S., Muro H., Nozoe S., Okuda S., Sato Z. (1972). Isolation of 3,4-dihydro-3,4,8-trihydroxy-1(2H)-naphthalenone and tenuazonic acid from Pyricularia oryzae cavara. Tetrahedron Lett..

[33] Masi M., Meyer S., Górecki M., Mandoli A., Di Bari L., Pescitelli G., Cimmino A., Cristofaro M., Clement S., Evidente A. (2017). Pyriclins A and B, two monosubstituted hex-4-ene-2,3-diols and other phytotoxic metabolites produced by Pyricularia grisea isolated from buffelgrass (Cenchrus ciliaris). Chirality.

[34] Semar M., Anke H., Arendholz W.-R., Veiten R., Steglich W. (1996). Lachnellins A, B, C, D, and Naphthalene-l,3,8-triol, Biologically Active Compounds from a Lachnellula Species (Ascomycetes). Z. Naturforsch. C J. Biosci..

[35] Ayer W.A., Trifonov L.S., Hutchison L.J., Chakravarty P. (2000). Metabolites from a Wood-Inhabiting Cup Fungus, Urnula craterium. Nat. Prod. Lett..

[36] Morita T., Aoki H. (1974). Isosclerone, a New Metabolite of Sclerotinia sclerotiorum (LIB.) DE BARY. Agric. Biol. Chem..

[37] Venkatasubbaiah P., Chilton W.S. (1992). Phytotoxins produced by Tubakia dryina. Mycopathologia.

[38] Bürki N., Michel A., Tabacchi R. (2003). Naphthalenones and isocoumarins of the fungus Ceratocystis fimbriata f. sp. platani. Mediterranna.

[39] Stierle A.A., Upadhyay R., Hershenhorn J., Strobel G.A., Molina G. (1991). The phytotoxins ofMycosphaerella fijiensis, the causative agent of Black Sigatoka disease of bananas and plantains. Experientia.

[40] Gremaud G., Tabacchi R. (1996). Relationship between the fungus Ceratocystis fimbriata coffea and the canker disease of the coffee tree. Phytochemistry.

[41] Evidente A., Sparapano L., Andolfi A., Bruno G. (2000). Two naphthalenone pentaketides from liquid cultures of Phaeoacremonium aleophilum, a fungus associated with esca of grapevine. Phytopathol. Mediterr..

[42] Nakamura T., Supratman U., Harneti D., Maharani R., Koseki T., Shiono Y. (2020). New compounds from Japanese oak wilt disease-associated fungus Raffaelea quercivora. Nat. Prod. Res..

[43] Shan R., Stadler M., Anke H., Sterner O. (1997). Naphthalenone and Phthalide Metabolites from Lachnum papyraceum. J. Nat. Prod..

[44] Cimmino A., Villegas-Fernández A.M., Andolfi A., Melck D., Rubiales D., Evidente A. (2011). Botrytone, a New Naphthalenone Pentaketide Produced by Botrytis fabae, the Causal Agent of Chocolate Spot Disease on Vicia faba. J. Agric. Food Chem..

[45] Burruano S., Giambra S., Mondello V., Dellagreca M., Basso S., Tuzi A., Andolfi A. (2016). Naphthalenone polyketides produced by Neofusicoccum parvum, a fungus associated with grapevine Botryosphaeria dieback. Phytopathol. Mediterr..

[46] Masi M., Nocera P., Zonno M.C., Tuzi A., Pescitelli G., Cimmino A., Boari A., Infantino A., Vurro M., Evidente A. (2018). Lentiquinones A, B, and C, Phytotoxic Anthraquinone Derivatives Isolated from Ascochyta lentis, a Pathogen of Lentil. J. Nat. Prod..

[47] Otomo N., Sato H., Sakamura S. (1983). Novel phytotoxins produced by the causal fungus of the shoot blight of larches. Agric. Biol. Chem..

[48] Abad P., Gouzy J., Aury J.M., Castagnone-Sereno P., Danchin E.G.J., Deleury E., Perfus-Barbeoch L., Anthouard V., Artiguenave F., Blok V.C. (2008). Genome sequence of the metazoan plant-parasitic nematode Meloidogyne incognita. Nat. Biotechnol..

[49] Chen J., Song B. (2021). Natural nematicidal active compounds: Recent research progress and outlook. J. Integrat. Agricul..

[50] Dong J.Y., Song H.C., Li J.H., Tang Y.S., Sun R., Wang L., Zhou Y.P., Wang L.M., Shen K.Z., Wang C.R. (2008). Ymf 1029A−E, Preussomerin Analogues from the Fresh-Water-Derived Fungus YMF 1.01029. J. Nat. Prod..

[51] Zhu Y., Dong J., Wang L., Zhou W., Li L., He H., Liu H., Zhang K. (2008). Screening and isolation of antinematodal metabolites againstBursaphelenchus xylophilus produced by fungi. Ann. Microbiol..

[52] Prestinaci F., Pezzotti P., Pantosti A. (2015). Antimicrobial resistance: A global multifaceted phenomenon. Pathog. Glob. Health.

[53] Fair R.J., Tor Y. (2014). Antibiotics and Bacterial Resistance in the 21st Century. Perspect. Med. Chem..

[54] Inácio M.L., Silva G.H., Teles H.L., Trevisan H.C., Cavalheiro A.J., Bolzani V.D.S., Young M.C., Pfenning L.H., Araújo R. (2006). Antifungal metabolites from Colletotrichum gloeosporioides, an endophytic fungus in Cryptocarya mandioccana Nees (Lauraceae). Biochem. Syst. Ecol..

[55] Suleiman M., McGaw L., Naidoo V., Eloff J. (2010). Detection of antimicrobial compounds by bioautography of different extracts of leaves of selected South African tree species. Afr. J. Tradit. Complement. Altern. Med..

[56] Findlay J.A., Kwan D. (1973). Metabolites from a Scytalidium Species. Can. J. Chem..

[57] Li Y.-X., Himaya S., Dewapriya P., Kim H.J., Kim S.-K. (2014). Anti-proliferative effects of isosclerone isolated from marine fungus Aspergillus fumigatus in MCF-7 human breast cancer cells. Process Biochem..

[58] El-Elimat T., Raja H.A., Figueroa M., Swanson S.M., Iii J.O.F., Lucas D.M., Grever M.R., Wani M.C., Pearce C.J., Oberlies N.H. (2014). Sorbicillinoid analogs with cytotoxic and selective anti-Aspergillus activities from Scytalidium album. J. Antibiot..

[59] Lu S., Draeger S., Schulz B., Krohn K., Ahmed I., Hussain H., Yi Y., Li L., Zhang W. (2011). Bioactive *Aromatic* Derivatives from Endophytic Fungus, *Cytospora* sp.. Nat. Prod. Commun..

[60] Pittayakhajonwut P., Sohsomboon P., Dramae A., Suvannakad R., Lapanun S., Tantichareon M. (2008). Antimycobacterial Substances from Phaeosphaeria sp BCC8292. Planta Med..

[61] Yuan C., Li G., Wu C.-S., Wang H.-Y., Zhao Z.-T., Lou H.-X. (2015). A New Fatty Acid from the Endolichenic Fungus *Massarina* sp.. Chem. Nat. Compd..

[62] Kim K.H., Beemelmanns C., Murillo C., Guillén A., Umaña L., Tamayo-Castillo G., Kim S.-N., Clardy J., Cao S. (2014). Naphthalenones and Isocoumarins from a Costa Rican Fungus Xylariaceae sp. CR1546C. J. Chem. Res..

[63] Wu J.-T., Zheng C.-J., Zhang B., Zhou X.-M., Zhou Q., Chen G.-Y., Zeng Z.-E., Xie J.-L., Han C.-R., Lyu J.-X. (2018). Two new secondary metabolites from a mangrove-derived fungus Cladosporium sp. JJM22. Nat. Prod. Res..

[64] Kongyen W., Rukachaisirikul V., Phongpaichit S., Sakayaroj J. (2015). A new hydronaphthalenone from the mangrove-derived *Daldinia eschscholtzii* PSU-STD57. Nat. Prod. Res..

[65] Prabpai S., Wiyakrutta S., Sriubolmas N., Kongsaeree P. (2015). Antimycobacterial dihydronaphthalenone from the endophytic fungus Nodulisporium sp. of Antidesma ghaesembilla. Phytochem. Lett..

[66] Sun R., Gao Y.-X., Shen K.-Z., Xu Y.-B., Wang C.-R., Liu H.-Y., Dong J.-Y. (2011). Antimicrobial metabolites from the aquatic fungus Delitschia corticola. Phytochem. Lett..

[67] Isaka M., Yangchum A., Rachtawee P., Khoyaiklang P., Boonyuen N., Lumyong S. (2009). Dihydronaphthalenones from the endophytic fungus Botryosphaeria sp. BCC 8200. Phytochem. Lett..

[68] Liu C.H., Meng J.C., Zou W.X., Huang L.L., Tang H.Q., Tan R.X. (2002). Antifungal carbon skeleton from Keissleriella sp. Y4108, a marine filamentous fungus. Planta Med..

[69] Shushni M.A.M., Mentel R., Lindequist U., Jansen R. (2009). Balticols A-F, New Naphthalenone Derivatives with Antiviral Activity, from an Ascomycetous Fungus. Chem. Biodivers..

[70] Sommart U., Rukachaisirikul V., Sukpondma Y., Phongpaichit S., Sakayaroj J., Kirtikara K. (2008). Hydronaphthalenones and a Dihydroramulosin from the Endophytic Fungus PSU-N24. Chem. Pharm. Bull..

[71] Trisuwan K., Khamthong N., Rukachaisirikul V., Phongpaichit S., Preedanon S., Sakayaroj J. (2010). Anthraquinone, Cyclopentanone, and Naphthoquinone Derivatives from the Sea Fan-Derived Fungi Fusarium spp. PSU-F14 and PSU-F135. J. Nat. Prod..

[72] Feng Y., Wang L., Niu S., Li L., Si Y., Liu X., Che Y. (2012). Naphthalenones from a Perenniporia sp. Inhabiting the Larva of a Phytophagous Weevil, Euops chinesis. J. Nat. Prod..

[73] Xu Y.-H., Lu C.-H., Zheng Z.-H., Shen Y.-M. (2011). New Polyketides Isolated from Botryosphaeria australis Strain ZJ12-1A. Helvetica Chim. Acta.

[74] Kornsakulkarn J., Dolsophon K., Boonyuen N., Boonruangprapa T., Rachtawee P., Prabpai S., Kongsaeree P., Thongpanchang C. (2011). Dihydronaphthalenones from endophytic fungus Fusarium sp. BCC14842. Tetrahedron.

[75] Amand S., Vallet M., Guedon L., Genta-Jouve G., Wien F., Mann S., Dupont J., Prado S., Nay B. (2017). A Reactive Eremophilane and Its Antibacterial 2(1H)-Naphthalenone Rearrangement Product, Witnesses of a Microbial Chemical Warfare. Org. Lett..

[76] Ai W., Lin X., Wang Z., Lü X., Mangaladoss F., Yang X., Zhou X., Tu Z., Liu Y. (2014). Cladosporone A, a new dimeric tetralone from fungus Cladosporium sp. KcFL6’ derived of mangrove plant Kandelia candel. J. Antibiot..

[77] Li H.-L., Li X.-M., Mándi A., Antus S., Li X., Zhang P., Liu Y., Kurtán T., Wang B.-G. (2017). Characterization of Cladosporols from the Marine Algal-Derived Endophytic Fungus Cladosporium cladosporioides EN-399 and Configurational Revision of the Previously Reported Cladosporol Derivatives. J. Org. Chem..

[78] Bai M., Zheng C.-J., Tang D.-Q., Zhang F., Wang H.-Y., Chen G.-Y. (2019). Two new secondary metabolites from a mangrove-derived fungus Cladosporium sp. JS1-2. J. Antibiot..

[79] Zhang F., Zhou L., Kong F., Ma Q., Xie Q., Li J., Dai H., Guo L., Zhao Y. (2020). Altertoxins with Quorum Sensing Inhibitory Activities from The Marine-Derived Fungus Cladosporium sp. KFD33. Mar. Drugs.

[80] Ferlay J., Ervik M., Lam F., Colombet M., Mery L., Piñeros M., Znaor A., Soerjomataram I., Bray F. (2020). Global Cancer Observatory: Cancer Today. Lyon: International Agency for Research on Cancer. https://gco.iarc.fr/today.

[81] Senapati S., Mahanta A.K., Kumar S., Maiti P. (2018). Controlled drug delivery vehicles for cancer treatment and their performance. Signal Transduct. Target. Ther..

[82] Wang Y., Liu H.-X., Chen Y.-C., Sun Z.-H., Li H.-H., Li S.-N., Yan M.-L., Zhang W.-M. (2017). Two New Metabolites from the Endophytic Fungus Alternaria sp. A744 Derived from Morinda officinalis. Molecules.

[83] Fujimoto Y., Yokoyama E., Takahashi T., Uzawa J., Morooka N., Tsunoda H., Tatsuno T. (1986). Studies on the metabolites of Penicillium diversum var. aureum. I. Chem. Pharm. Bull..

[84] El-Amrania M., Ebadab S.S., Gadb H.A., Proksch P. (2016). Pestalotiopamide E and pestalotiopin B from an endophytic fungus Aureobasidium pullulans isolated from Aloe vera leaves. Phytochem. Lett..

[85] Tang J.-W., Wang W.-G., Li A., Yan B.-C., Chen R., Li X.-N., Du X., Sun H.-D., Pu J.-X. (2017). Polyketides from the endophytic fungus *Phomopsis* sp. sh917 by using the one strain/many compounds strategy. Tetrahedron.

[86] Flores-Bocanegra L., Raja H.A., Bacon J.W., Maldonado A.C., Burdette J.E., Pearce C.J., Oberlies N.H. (2020). Cytotoxic Naphthoquinone Analogues, Including Heterodimers, and Their Structure Elucidation Using LR-HSQMBC NMR Experiments. J. Nat. Prod..

[87] Li L.Y., Sun B.D., Zhang G.S., Deng H., Wang M.H., Tan X.M., Zhang X.Y., Jia H.M., Zhang H.W., Zhang T. (2017). Polyketides with different post-modifications from desert endophytic fungus *Paraphoma* sp.. Nat. Prod. Res..

[88] Abdelwahab M.F., Kurtán T., Mándi A., Müller W.E.G., Fouad M.A., Kamel M.S., Liu Z., Ebrahim W., Daletos G., Proksch P. (2018). Induced secondary metabolites from the endophytic fungus Aspergillus versicolor through bacterial co-culture and OSMAC ap-proaches. Tetrahedron Lett..

[89] Yang Z., Ding J., Ding K., Chen D., Cen S., Ge M. (2013). Phomonaphthalenone A: A novel dihydronaphthalenone with anti-HIV activity from *Phomopsis* sp. HCCB04730. Phytochem. Lett..

[90] Zhang P., Jia C., Lang J., Li J., Luo G., Chen S., Yan S., Liu L. (2018). Mono- and Dimeric Naphthalenones from the Marine-Derived Fungus Leptosphaerulina chartarum 3608. Mar. Drugs.

[91] Zurlo D., Leone C., Assante G., Salzano S., Renzone G., Scaloni A., Foresta C., Colantuoni V., Lupo A. (2013). Cladosporol a stimulates G1-phase arrest of the cell cycle by up-regulation of p21waf1/cip1 expression in human colon carcinoma HT-29 cells. Mol. Carcinog..

[92] Zurlo D., Assante G., Moricca S., Colantuoni V., Lupo A. (2014). Cladosporol A, a new peroxisome proliferator-activated receptor γ (PPARγ) ligand, inhibits colorectal cancer cells proliferation through β-catenin/TCF pathway inactivation. Biochim. Biophys. Acta.

[93] Koul M., Kumar A., Deshidi R., Sharma V., Singh R.D., Singh J., Sharma P.R., Shah B.A., Jaglan S., Singh S. (2017). Cladosporol A triggers apoptosis sensitivity by ROS-mediated autophagic flux in human breast cancer cells. BMC Cell Biol..

[94] Zhang Z., He X., Liu C., Che Q., Zhu T., Gu Q., Li D. (2016). Clindanones A and B and cladosporols F and G, polyketides from the deep-sea derived fungus Cladosporium cladosporioides HDN14-342. RSC Adv..

[95] Fan Z., Sun Z.-H., Liu H., Chen Y.-C., Li H.-H., Zhang W.-M. (2016). Perangustols A and B, a pair of new azaphilone epimers from a marine sediment-derived fungus Cladosporium perangustm FS62. J. Asian Nat. Prod. Res..

[96] Rukachaisirikul V., Sommart U., Phongpaichit S., Hutadilok-Towatana N., Rungjindamai N., Sakayaroj J. (2007). Metabolites from the Xylariaceous Fungus PSU-A80. Chem. Pharm. Bull..

[97] Abdel-Lateff A., König G.M., Fisch K.M., Höller U., Jones P.G., Wright A.D. (2002). New Antioxidant Hydroquinone Derivatives from the Algicolous Marine Fungus Acremonium sp.. J. Nat. Prod..

[98] Ibrahim S.R.M., Mohamed G.A., El-Messery S.M. (2016). In silico Modeling Studies of 5-HT2B Antagonistic Activity of 2-(2- phenylethyl)chromone Derivatives from Cucumis melo Seeds. Lett. Drug Des. Discov..

[99] Costagliola C., Parmeggiani F., Semeraro F., Sebastiani A. (2008). Selective Serotonin Reuptake Inhibitors: A Review of its Effects on Intraocular Pressure. Curr. Neuropharmacol..

[100] Peng Y., Zhao S., Wu Y., Cao H., Xu Y., Liu X., Shui W., Cheng J., Zhao S., Shen L. (2018). Identification of natural products as novel ligands for the human 5-HT2C receptor. Biophys. Rep..

[101] Bös M., Canesso R., Inoue-Ohga N., Nakano A., Takehana Y., Sleight A.J. (1997). O-Methylasparvenone, a nitrogen-free serotonin antagonist. Bioorg. Med. Chem..

[102] Bell A.A., Wheeler M.H. (1986). Biosynthesis and functions of fungal melanins. Annu. Rev. Phytopathol..

[103] Lee J.-K., Jung H.-M., Kim S.-Y. (2003). 1,8-Dihydroxynaphthalene (DHN)-Melanin Biosynthesis Inhibitors Increase Erythritol Production in Torula corallina, and DHN-Melanin Inhibits Erythrose Reductase. Appl. Environ. Microbiol..

[104] Thines E., Anke H., Sterner O. (1998). Scytalols A, B, C, and D and other modulators of melanin biosynthesis from Scytalidium sp. 36-93. J. Antibiot..

[105] Nong X.-H., Zheng Z.-H., Zhang X.-Y., Lu X.-H., Qi S.-H. (2013). Polyketides from a Marine-Derived Fungus Xylariaceae sp.. Mar. Drugs.

[106] Cui H., Zhang H., Liu Y., Gu Q., Xu J., Huang X., She Z. (2018). Ethylnaphthoquinone derivatives as inhibitors of indoleamine-2, 3-dioxygenase from the mangrove endophytic fungus Neofusicoccum austral SYSU-SKS024. Fitoterapia.

[107] Xiao W.-J., Chen H.-Q., Wang H., Cai C.-H., Mei W.-L., Dai H.-F. (2018). New secondary metabolites from the endophytic fungus Fusarium sp. HP-2 isolated from “Qi-Nan” agarwood. Fitoterapia.

[108] Medzhitov R. (2010). Inflammation 2010: New Adventures of an Old Flame. Cell.

[109] Yatoo M.I., Gopalakrishnan A., Saxena A., Parray O.R., ALAM Tufani N., Chakraborty S., Tiwari R., Dhama K., Iqbal H. (2018). Anti-Inflammatory Drugs and Herbs with Special Emphasis on Herbal Medicines for Countering Inflammatory Diseases and Disorders—A Review. Recent Patents Inflamm. Allergy Drug Discov..

[110] Fizeșan I., Rusu M.E., Georgiu C., Pop A., Ștefan M.G., Muntean D.M., Mirel S., Vostinaru O., Kiss B., & Popa D.S. (2021). Antitussive, Antioxidant, and Anti-Inflammatory Effects of a Walnut (Juglans regia L.) Septum Extract Rich in Bioactive Compounds. Antioxidants.

[111] Liu W., Chen S., Li J., Yang X., Yan C., Liu H. (2018). A new β-tetralonyl glucoside from the Santalum album derived endophytic fungus Colletotrichum sp. GDMU-1. Nat. Prod. Res..

[112] Girich E.V., Yurchenko A.N., Smetanina O.F., Trinh P.T.H., Ngoc N.T.D., Pivkin M.V., Popov R.S., Pislyagin E.A., Menchinskaya E.S., Chingizova E.A. (2020). Neuroprotective Metabolites from Vietnamese Marine Derived Fungi of *Aspergillus* and *Penicillium* Genera. Mar. Drugs.

[113] Zhao D.-L., Shao C.-L., Wang C.-Y., Wang M., Yang L.-J., Wang C.-Y. (2016). Naphthalenones and Depsidones from a Sponge-Derived Strain of the Fungus Corynespora cassiicola. Molecules.

[114] He Z.H., Zhang G., Yan Q.X., Zou Z.B., Xiao H.X., Xie C.L., Tang X.X., Luo L.Z., Yang X.W. (2020). Cladosporactone A, a unique polyketide with 7-methylisochromen-3-one skeleton from the deep-sea-derived fungus Cladosporium cladosporioides. Chem. Biodivers..

[115] Zhong J.-J., Xiao J.-H. (2009). Secondary Metabolites from Higher Fungi: Discovery, Bioactivity, and Bioproduction. Adv. Biochem. Eng. Biotechnol. China I.

[116] Keller N.P. (2019). Fungal secondary metabolism: Regulation, function and drug discovery. Nat. Rev. Microbiol..

[117] Wu B., Hussain M., Zhang W., Stadler M., Liu X., Xiang M. (2019). Current insights into fungal species diversity and perspective on naming the environmental DNA sequences of fungi. Mycology.

[118] Hemingway R.W., McGraw G.W., Barras S.J. (1977). Polyphenols in *Ceratocystis minor* infected *Pinus* taeda: Fungal metabolites, phloem and xylem phenols. J. Agric. Food Chem..

[119] Stipanovic R.D., Bell A.A. (1977). Pentaketide metabolites of *Verticillium dahliae*. II. Accumulation of naphthol derivatives by the aberrant-melanin mutant BRM-2. Mycologia.

[120] Ayer W.A., Browne L.M., Lin G. (1989). Metabolites of Leptographium wageneri, the causative agent of black stain root disease of conifers. J. Nat. Prod..

[121] Borgschulte K., Rebuffat S., Trowitzsch-Kienast W., Schomburg D., Pinon J., Bodo B. (1991). Isolation and structure elucidation of hymatoxins B–E and other phytotoxins from *Hypoxylon mammatum* fungal pathogen of leuce poplars. Tetrahedron.

[122] Tabuchi H., Tajimi A., Ichihara A. (1994). Phytotoxic metabolites isolated from *Scolecotrichum graminis* Fuckel. Biosci. Biotech. Biochem..

[123] Trisuwan K., Rukachaisirikul V., Sukpondma Y., Preedanon S., Phongpaichit S., Rungjindamai N., Sakayaroj J. (2008). Epoxydons and a pyrone from the marine-derived fungus *Nigrospora* sp. PSU-F5. J. Nat. Prod..

[124] Arunpanichlert J., Rukachaisirikul V., Phongpaichit S., Supaphon O., Sakayaroj J. (2016). Xylariphilone: A new azaphilone derivative from the seagrass-derived fungus *Xylariales* sp. PSU-ES163. Nat. Prod. Res..

[125] Fan C., Zhou G., Wang W., Zhang G., Zhu T., Che Q., Li D. (2021). Tetralone derivatives from a deep-sea derived fungus *Cladosporium* sp. HDN17-58. Nat. Prod. Commun..

[126] Sviridov S.I. (1991). Secondary metabolites of *Pyricularia oryzae*. Chem. Nat. Compd..

[127] Zhang Y., Feng Y., Kramer M., Essmann F., Grond S. (2017). A New Acetylenic compound and other bioactive metabolites from a shark gill-derived *Penicillium* strain. Rec. Nat. Prod..

[128] Quang T.H., Huong P.T.M., Ngan N.T.T., Hanh T.T.H., Cuong N.X., Nam N.H., Minh C.V. (2020). Secondary metabolites from a marine sponge-associated fungus *Xenomyrothecium* sp. IMBC-FP2.11. Vietnam J. Chem..

[129] Iwasaki S., Muro H., Sasaki K., Nozoe S., Okuda S., Sato Z. (1973). Isolations of phytotoxic substances produced by pyricularia oryzae cavara. Tetrahedron Lett..

[130] Krohn K., Biele C., Drogies K.H., Steingrover K., Aust H.J., Draeger S., Schulz B. (2002). Fusidilactones, a new group of polycyclic lactones from an endophyte, *Fusidium* sp.. Eur. J. Org. Chem..

[131] Yamaguchi Y., Masuma R., Kim Y.-P., Uchida R., Tomoda H., Omura S. (2004). Taxonomy and secondary metabolites of *Pseudobotrytis* sp. FKA-25. Mycoscience.

[132] Mancilla G., Jiménez-Teja D., Femenía-Rios M., Macías-Sánchez A.J., Collado I.G., Hernández-Galán R. (2009). Novel macrolide from wild strains of the phytopathogen Fungus *Colletotrichum acutatum*. Nat. Prod. Commun..

[133] Ouchbani T., Zouihri H., Essassi E., Proksch P., Ng S.W. (2012). (3*R*,4*S*)-3,4,8-Trihydroxy-1,2,3,4-tetrahydronaphthalen-1-one monohydrate from Embellisia eureka. Acta Cryst..

[134] Venkatasubbaiah P., Chilton W.S.J. (1991). Toxins produced by the Dogwood Anthracnose fungus *Discula* sp.. J. Nat. Prod..

[135] Höller U., Konig G.M., Wright A.D. (1999). Three new metabolites from marine-derived fungi of the genera *coniothyrium* and *microsphaeropsis*. J. Nat. Prod..

[136] Xie F., Chang W., Zhang M., Li Y., Li W., Shi H., Zheng S., Lou H. (2016). Quinone derivatives isolated from the endolichenic fungus *Phialocephala fortinii* are Mdr1 modulators that combat azole resistance in *Candida albicans*. Sci. Rep..

[137] De Souza E.M.C., Da Silva E.L., Marinho A.M.R., Marinho P.S.B. (2016). (4*S*)-4,8-dihydroxy-1-tetralone and other chemical constituents from *Pestalotiopsis* sp. EJC07, endophytic from *Bauhinia guianensis*. An. Acad. Bras. Ciênc..

[138] Hsiao Y., Cheng M.J., Chang H.S., Wu M.D., Hsieh S.Y., Liu T.W., Lin C.H., Yuan G.F., Chen I.S. (2016). Six new metabolites produced by *Colletotrichum aotearoa* 09F0161, an endophytic fungus isolated from *Bredia oldhamii*. Nat. Prod. Res..

[139] Kokubun T., Veitch N.C., Bridge P.D., Simmonds M.S.J. (2003). Dihydroisocoumarins and a tetralone from *Cytospora eucalypticola*. Phytchemistry.

[140] Gallo M.B.C., Cavalcanti B.C., Barros F.W., de Moraes M.O., Costa-Lotufo L.V., Pessoa C., Bastos J.K., Pupo M.T. (2010). Chemical constituents of *Papulaspora immersa*, an endophyte from *Smallanthus sonchifolius* (Asteraceae), and their cytotoxic activity. Chem. Biodivers..

[141] Evidente A., Punzo B., Andolfi A., Cimmino A., Melck D., Luque J. (2010). Lipophilic phytotoxins produced by *Neofusicoccum parvum*, a grapevine canker agent. Phytopathol. Mediterr..

[142] El-Elimat T., Raja H.A., Figueroa M., Falkinham J.O., Oberlies N.H. (2014). Isochromenones, isobenzofuranone, and tetrahydronaphthalenes produced by *Paraphoma radicina*, a fungus isolated from a freshwater habitat. Phytochemistry.

[143] Sadorn K., Saepua S., Boonyuen N., Boonruangprapa T., Rachtawee P., Pittayakhajonwut P. (2018). Antimicrobial activity and cytotoxicity of xanthoquinodin analogs from the fungus *Cytospora eugeniae* BCC42696. Phytochemistry.

[144] Yang H.X., Peng X.P., Gao H., Zhang H.M., Wang Z.R., Li G., Lou H.X. (2021). Pleosporalins H and I, two new heptaketides from the endophytic fungus *Pleosporales* sp. F46 by using OSMAC strategy. Nat. Prod. Res..

[145] Yang X.-Y., Zhang J.-X., Ding Q.-Y., He Z.-C., Zhu C.-Y., Zhang K.-Q., Niu X.-M. (2020). Metabolites from two dominant thermophilic fungal species *Thermomyces lanuginosus* and *Scytalidium thermophilum*. Chem. Biodivers..

[146] Sato H., Takishima T., Otomo N., Sakamura S. (1982). Phytotoxins produced by the fungus of the larch shoot bligh. Nippon Nôgeikagaku Kaishi..

[147] Luo G., Chen S., Yu J., Yuan J., Zheng L., Liu L., Chen B., Li J. (2020). Naphthalenones and naphthols isolated from the *Saussurea laniceps* endophytic fungus *Didymella glomerata* X223. Chem. Biodivers..

[148] Bell A.A., Stipanovic R.D., Puhalla J.E. (1976). Pentaketide metabolites of *Verticillium dahliae*: Identification of (+)-scytalone as a natural precursor to melanin. Tetrahedron.

[149] Liu Y., Stuhldreier F., Kurtan T., Mandi A., Arumugam S., Lin W., Stork B., Wesselborg S., Weber H., Henrich B. (2017). Daldinone derivatives from the mangrove-derived endophytic fungus *Annulohypoxylon* sp.. RSC Adv..

[150] Zhang J., Liang J.H., Zhao J.C., Wang Y.L., Dong P.P., Liu X.G., Zhang T.Y., Wu Y.Y., Shang D.J., Zhang Y.X. (2018). Xylarianins A-D from the endophytic fungus *Xylaria* sp. SYPF 8246 as natural inhibitors of human carboxylesterase 2. Bioorg. Chem..

[151] Padumadasa C., Xu Y.M., Wijeratne E.M.K., Espinosa-Artiles P., U’Ren J.M., Arnold A.E., Gunatilaka A.A.L. (2018). Cytotoxic and noncytotoxic metabolites from *Teratosphaeria* sp. FL2137, a fungus associated with *Pinus clausa*. J. Nat. Prod..

[152] Bartman C.D., Campbell I.M. (1979). Naphthalenone production in *Aspergillus parvulus*. Can. J. Microbiol..

[153] Hao G., Qing-Hua Z., Miao-Miao J., Jin-Shan T., Cheng-Du M., Kui H., Michio N., Nai-Li W., Xin-Sheng Y. (2008). Polyketides from a marine sponge-derived fungus *Mycelia sterilia* and proton-proton long-range coupling. Magn. Reson. Chem..

[154] Rivera-Chávez J., El-Elimat T., Gallagher J.M., Graf T.N., Fournier J., Panigrahi G.K., Deep G., Bunch R.L., Raja H.A., Oberlies N.H. (2019). Delitpyrones: α-Pyrone derivatives from a freshwater *Delitschia* sp.. Planta Med..

[155] Wu Z.-C., Li D.-L., Chen Y.-C., Zhang W.-M. (2010). A new isofuranonaphthalenone and benzopyrans from the endophytic fungus *Nodulisporium* sp. A4 from *Aquilaria sinensis*. Helv. Chim. Acta.

[156] Zhou Y.H., Zhang M., Zhu R.X., Zhang J.Z., Xie F., Li X.B., Chang W.Q., Wang X.N., Zhao Z.T., Lou H.X. (2016). Heptaketides from an endolichenic fungus *Biatriospora* sp. and their antifungal activity. J. Nat. Prod..

[157] Ariefta N.R., Nikmawahda H.T., Koseki T., Shiono Y. (2019). Fusopoltides B–E, new polyketides isolated from Fusarium solani B-18. Tetrahedron Lett..

[158] Niu S., Tang X.X., Fan Z., Xia J.M., Xie C.L., Yang X.W. (2019). Fusarisolins A–E, polyketides from the marine-derived fungus *Fusarium solani* H918. Mar. Drugs.

[159] Itsuo K., Shunji S., Akihiko M., Akio F. (1987). 5-Hydroxydihydrofusarubin, a Process for Its Preparation and Its Use as a Medicament. European Patent.

[160] Wen Y., Lv Y., Hao J., Chen H., Huang Y., Liu C., Huang H., Ma Y., Yang X. (2020). Two new compounds of *Penicillium polonicum*, an endophytic fungus from *Camptotheca acuminata* Decne. Nat. Prod. Res..

[161] Stodůlková E., Man P., Kuzma M., Černý J., Císařová I., Kubátová A., Chudíčková M., Kolařík M., Flieger M. (2015). A highly diverse spectrum of naphthoquinone derivatives produced by the endophytic fungus *Biatriospora* sp. CCF 4378. Folia Microbiol. (Praha).

[162] Sakagami Y., Sano A., Marumo S., Yoshikawa N., Nakagawa J. (1992). Cladosporol, a plant growth regurator produced by fungus *Cladosporium cladosporioides*. Symp. Chem. Nat. Prod..

[163] Sakagami Y., Sano A., Hara O., Mikawa T., Marumo S. (1995). Cladosporol, β-l,3-glucan biosynthesis inhibitor, isolated from fungus, *Cladosporium cladosporioides*. Tetrahedron Lett..

[164] Zurlo D., Ziccardi P., Votino C., Colangelo T., Cerchia C., Dal Piaz F., Dallavalle S., Moricca S., Novellino E., Lavecchia A. (2016). The antiproliferative and proapoptotic effects of cladosporols A and B are related to their different binding mode as PPARγ ligands. Biochem. Pharmacol..

[165] Nasini G., Arnone A., Assante G., Bava A., Moricca S., Ragazzi A. (2004). Secondary mould metabolites of *Cladosporium tenuissimum*, a hyperparasite of rust fungi. Phytochemistry.

[166] Yamazaki H., Yagi A., Akaishi M., Kirikoshi R., Takahashi O., Abe T., Chiba S., Takahashi K., Iwakura N., Namikoshi M. (2018). Halogenated cladosporols produced by the sodium halide-supplemented fermentation of the plant-associated fungus *Cladosporium* sp. TMPU1621. Tetrahedron Lett..

[167] Fan Z., Sun Z., Chen Y., Li H., Zhang W. (2016). Cladosperanol A, a new dimeric tetralone from marine-derived fungus *cladosporium perangustum* FS62. Nat. Prod. Res. Dev..

