# Supplementary material for: Fungal Naphthalenones; Promising Metabolites for Drug Discovery: Structures, Biosynthesis, Sources, and Pharmacological Potential
Source: Toxins (Basel). 2022 Feb 19;14(2):154. doi: 10.3390/toxins14020154 (PMC8879409; doi:10.3390/toxins14020154)
Supplement: Supplementary file 1 [file toxins-14-00154-s001.zip › toxins-1580666-supplementary.pdf]

toxins

Review

Fungal Naphthalenones; Promising Metabolites for Drug Discovery: Structures, Biosynthesis, Sources, and Pharmacological Potential

Sabrin R. M. Ibrahim, Sana A. Fadil, Haifa A. Fadil, Bayan A. Eshmawi, Shaimaa G. A. Mohamed and Gamal A. Mohamed

Table S1. List of fungal naphthalenones (Fungal source, host, and place).

Compound name	Mol. Wt.	Mol. formula	Fungal source	Host (part, family)	Location	Ref.
3,4-Dihydro-3,4,8-trihydroxy-1(2H)-naphthalenone (= 3,4-Dihydro-3,4,8-trihydroxy-1(2*H*)-naphthalenone = (3S,4S)-3,4,8- Trihydroxy-3,4-dihydro -1(2H)-naphthalenone = 3-Hydroxyisosclerone) (**1**)	194	$C_{10}H_{10}O_4$	*Pyricularia oryzae* Cav. Ken 53-33	Rice blast	Japan	[33]
			Ceratocystis minor (Hedgc.) Hunt	*Dendroctonus frontalis* (Insect, Scolytidae)	Southern USA	[118]
			Verticillium dahlia brm-2 mutant	Cultured	USA	[119]
			Leptographium wageneri RH50 (Kendr) var. *pserrabtsugae* = *Verticicladzella wageneri* Kendrick	*Pseudotsuga menziesii* (Douglas fir, Pinaceae)	USA	[120]
			Hypoxylon mammarum	*Populus tremula* (Canker margin of infected quaking aspen, Salicaceae)	France	[121]
			Tubakia dryina (Sacc.) PV-6	-	East of Mississippi River, USA	[63]
			Scolecotrichum graminis Fuckel	Cultured	Japan	[122]
			Ceratocystis fimbriata coffea	*Coffea arabica* (Canker disease, Rubiaceae)	China	[41]
			Nigrospora sp. PSU-F5	*Annella* sp. (Gorgonian Sea fan, Gorgoniidae)	Near Similan Island, Thailand	[123]
			Daldinia eschscholzii	*Mantis religiosa* (Mantis, Mantidae)	China	[27]
			Xylariaceae sp. CR1546C	*Sticta fuliginosa* (Lichen, Lobariaceae)	Costa Rica	[63]
			Aureobasidium pullulans	*Aloe vera* (Leaves, Asphodelaceae)	Marrakesh, Morroco	[84]
			Xylariales sp. PSU-ES163	*Halophila ovalis* (Seagrass leaves, Hydrocharitaceae)	Trang, Thailand	[124]
			Alternaria sp. A744	*Morinda officinalis* (Twigs, Rubiaceae)	Gaoyao city, Guangdong, China	[82]

			Cladosporium sp. JJM22	*Ceriops tagal* (Mangrove plant, Rhizophoraceae)	Dongzhaigang, Hainan, China	[65]
			Cladosporium sp. JJM22	*Ceriops tagal* (Mangrove plant, Rhizophoraceae)	South China Sea, Dongzhaigang, Hainan, China	[65]
			Cladosporium sp. HDN17-58	Deep-sea sediment	Western Pacific Ocean, China	[125]
trans-3,4,8-Trihydroxy-1(2*H*)-naphthalenone (= (+)-*trans*-3,4-Dihydro-3,4,8-trihydroxy-1(2H)-naphthalenone = (3*S*,4*S*)-4-Hydroxy-6-dehydroxyscytalone) (**2**)	194	$C_{10}H_{10}O_4$	*Pyricularia oryzae* Cav. (Ken 53-33)	Rice blast	Japan	[126]
			Lachnellula sp. A 32-89	*Polylepis sericea* (Bark, Rosaceae)	Laguna Negra, Venezuela.	[35
			Massarina sp.	*Leptogium hildenbrandii* (Lichen, Collemataceae)	Lijiang City, Yunnan, China	[62]
			Daldinia eschscholtzii	*Paphiopedilum exul* (Orchid, roots, Orchidaceae)	Chiang Mai, Thailand	[21]
			Pyricularia grisea	*Cenchrus ciliaris* (Buffelgrass, Poaceae)	Saguaro National Monument, Arizona, USA	[34]
			Penicillium polonicum AP2T1	*Isurus oxyrinchus* (Gills of shark, Lamnidae)	China	[127]
			Xenomyrothecium sp. IMBCFP2.11	Unidentified sponge	Quang Nam sea, Vietnam	[128]
			Penicillium sp. KMM 4672	*Padina* sp. (Brown alga, Dictyotaceae)	Van Phong Bay, South China Sea, Vietnam	[113]
(3*R*,4*S*)-3,4,8-Trihydroxy-3,4-dihydronaphthalen-1(2*H*)-one (= (3*R*,4*S*)-(+)-4-hydroxy-6-deoxyscytalone) (**3**)	194	$C_{10}H_{10}O_4$	*Pyricularia oryzae* Cav. (Ken 53-33)	Rice blast	Japan	[129]
			Sclerotinia sclerotiorum (LIB.) DE BARY.	Cultured	Japan	[37]
			Fusidium sp.	*Mentha arvense* (Leaves, Lamiaceae)	Hahausen, Lower Saxony, Germany	[130]

			Pseudobotrytis sp. FKA-25	Forest soil	Yakushima, Kagoshima Prefecture, Japan,	[131]
			Colletotrichum gloeosporioides	*Cryptocarya mandioccana* (Leaves, Lauraceae)	Cerrado and Atlantic Forest, Brazil	[55]
			Colletotrichum acutatum IMI 348489	*Fragaria ananassa* (Strawberry plant, Rosaceae)	France	
			Colletotrichum acutatum IMI 364856	*Fragaria ananassa* (Strawberry plant, Rosaceae)	Spain	
			Colletotrichum. acutatum UCA 1028	*Fragaria ananassa* (Strawberry plant, Rosaceae)	Conil, Cádiz	[132]
			Botrytis fabae Bf-CO-05	*Vicia faba* (Bean, Fabaceae)	Cordoba field, Institute for Sustainable Agriculture, CSIC, Cordoba, Spain	[44]
			Embellisia eureka	Cultured	Germany	[133]
			Xylariaceae sp. SCSGAF0086	*Melitodes squamata* (Gorgonian coral, Melithaeinae)	South China Sea, China	[134]
			Xylariaceae sp. CR1546C	*Sticta fuliginosa* (Lichen, Lobariaceae)	Costa Rica	[63]
(2*S*,4*S*)-3,4-Dihydro-2,4,8-trihydroxy-l(2*H*)-naphthalenone (= (-)-*trans*-3,4-Dihydro-2,4,8-trihydroxynaphthalen-1(2*H*)-one) (**4**)	194	$C_{10}H_{10}O_4$	*Urnula craterium* (Schw.: Fr.)	Cultured	Canada	[36]
			Ceratocystis fimbriata f. sp. *platani*	Cultured	Madonna del Piano, Ticino, Switzerland	[39]
			Botrytis fabae Bf-CO-05	*Vicia faba* (Bean, Fabaceae)	Cordoba field, Institute for Sustainable Agriculture, CSIC, Cordoba, Spain	[44]

			Alternaria sp. A744	*Morinda officinalis* (Twigs, Rubiaceae)	Gaoyao city, Guangdong, China	[82]
			Leptosphaerulina chartarum 3608	Crinoid (Marine animal, Crinoidea)	Xuwen, Zhanjiang City, Guangdong, China	[91]
(-)-*trans*-3,4-Dihydro-2,4,8–trihydroxynaphthalen-1(2*H*)-one (**5**)	194	$C_{10}H_{10}O_4$	*Ceratocystis fimbriata* f. sp. *platani*	Cultured	Madonna del Piano, Ticino, Switzerland	[39]
(+)-*cis*-3,4-Dihydro-2,4,8-trihydroxynaphthalen-1(2*H*)-one (**6**)	194	$C_{10}H_{10}O_4$	*Leptosphaerulina chartarum* 3608	Crinoid (Marine animal, Crinoidea)	Xuwen, Zhanjiang City, Guangdong, China	[91]
(2*S*,4*R*)-2,4,8-Trihydroxy-3,4-dihydronaphthalen-1(2*H*)-one (**7**)	194	$C_{10}H_{10}O_4$	*Mycosphaerella fijiensis* IMI 105378	*Musa sapientum* (Musaceae)	San Pedro Sula, Honduras	[40]
Sclerone (= (4*R*)-(-)-Regiolone = (4*R*)-3,4-Dihydro-4,8-diihydroxy-1(2*H*)-naphthalenone = 4,8-Dihydroxytetralone) (**8**)	178	$C_{10}H_{10}O_3$	*Sclerotinia sclerotiorum* (LIB.) DE BARY	Cultured	Japan	[37]
			Scytalidium sp.	Cultured	Japan	[57]
			Verticillium dahlia brm-2 mutant	Cultured	USA	[119]
			Penicillium diversum var. *aureum*	Cultured	Japan	[83]
			Discula sp.	*Cornus florida* (Cornaceae)	North Carolina and Georgia, USA	[135]
			Cladosporium cladosporioides *Cladosporium sphaerospermum*	*Cryptocarya mandioccana* (Leaves, Lauraceae)	Cerrado and Atlantic Forest, Brazil	[55]
			Caryospora callicarpa YMF1.01026	Submerged woody substrate, freshwater	Yunnan Province, China	[52]
			Colletotrichum acutatum IMI 348489	*Fragaria ananassa* (Strawberry plant, Rosaceae)	France	

			Colletotrichum acutatum IMI 364856	*Fragaria ananassa* (Strawberry plant, Rosaceae)	Spain	[132]
			Colletotrichum acutatum UCA 1028	*Fragaria ananassa* (Strawberry plant, Rosaceae)	Conil, Cádiz	
			Coniothyrium sp. 193H77	*Ectyplasia perox* (Marine sponges)	Caribbean Sea, Dominica	[136]
			Daldinia eschscholzii	*Mantis religiosa* (Mantis, Mantidae)	China	[27]
			Xylariaceae sp. SCSGAF0086	*Melitodes squamata* (Gorgonian coral, Melithaeinae)	South China Sea, China	[134]
			Phialocephala fortinii	*Pamelia* sp. (Lichen, Parmeliaceae)	Mount Qingliang, Zhejiang, China	[137]
			Pestalotiopsis sp. EJC07	*Bauhinia guianensis* (Fabaceae)	Belém-PA, Brasil	[138]
			Colletotrichum aotearoa 09F0161	*Bredia oldhamii* (Leaves, Melastomataceae)	Taiwan	[139]
			Cladosporium cladosporioides HDN14-342	Sediment	Indian Ocean, Qingdao, China	[95]
			Colletotrichum sp. GDMU-1	*Santalum album* (Leaves, Santalaceae)	Dongguan,Guangdong, China	[112]
			Cladosporium sp. JJM22	*Ceriops tagal* (Mangarove plant, Rhizophoraceae)	South China Sea, Dongzhaigang, Hainan, China	[65]
4(*S*)-(+)-Isosclerone (= (4*S*)-3,4-Dihydro- 4,8-diihydroxy-1(2*H*)-naphthalenone = (-)-(4*S*)-4,8-Dihydroxy-α-tetralone) (**9**)	178	$C_{10}H_{10}O_3$	*Sclerotinia sclerotiorum* (LIB.) DE BARY	Cultured	Japan	[37]
			Penicillium diversum var. *aureum*	Cultured	Japan	[83]
			Pyricularia oryzae Cav. (Ken 53-33)	Rice blast	Japan	[126]
			Tubakia dryina (Sacc.) PV-6	East of the Mississippi River	USA	[63]
			Scolecotrichum graminis Fuckel	Cultured	Japan	[122]
			Phaeoacremonium aleophilum (Pal) PVFi69-257	*Vitis vinifera* (Grapevine, Vitaceae)	Italy	[42]

	Urnula craterium (Schw.: Fr.)	Cultured	Canada	[36]
	Cytospora eucalypticola	*Eucalyptus perriniana* (Spinning gum bark, Myrtaceae)	Royal Botanic Gardens, Kew, UK	[140]
	Xylariaceous sp. PSU-A80	*Garcinia atroviridis* (Leaves, Clusiaceae)	Songkhla Province, Thailand	[97]
	Caryospora callicarpa YMF1.01026	Submerged woody substrate, freshwater	Yunnan Province, China	[52]
	Phaeosphaeria sp.	*Bambusa vulgaris* (Decaying bamboo stem, Poaceae)	Queen Sirikit Botanic Garden, Chiang Mai, Thailand	[61]
	Papulaspora immersa	*Smallanthus sonchifolius* (Leaves and roots, Asteraceae)	Ribeirão Preto, São Paulo State, Brazil.	[141]
	Neofusicoccum parvum	*Vitis vinifera* cv. *Parellada* (Cankered branch, Vitaceae)	Catalonia, NE, Spain	[142]
	Botrytis fabae Bf-CO-05	*Vicia faba* (Bean, Fabaceae)	Cordoba field, Institute for Sustainable Agriculture, CSIC, Cordoba, Spain	[44]
	Paraphoma radicina G104	Submerged wood, freshwater lake	Greensboro, North Carolina, USA	[143]
	Aspergillus fumigatus	Marine green algae, surface	Seosaeng-myeon, Ulsan, Korea	[58]
	Scytalidium album MSX51631	Cultured	Cobb County, GA, USA	[59]
	Daldinia eschscholtzii PSUSTD57	*Bruguiera gymnorrhiza* (Mangrove plant leaves, Rhizophoraceae)	Suratthani, Thailand	[64]

			Neofusicoccum parvum B19	*Vitis vinifera* (Declining vines of grapevine, Vitaceae)	Inzolia, Sicily	[46]
			Xylariales sp. PSU-ES163	*Halophila ovalis* (Seagrass leaves, Hydrocharitaceae)	Trang, Thailand	[124]
			Cladosporium cladosporioides HDN14-342	Sediment	Indian Ocean, Qingdao, China	[95]
			Pyricularia grisea	*Cenchrus ciliaris* (Buffelgrass, Poaceae)	Saguaro National Monument, Arizona, USA	[47]
			Alternaria sp. A744	*Morinda officinalis* (Twigs, Rubiaceae)	Gaoyao city, Guangdong, China	[82]
			Cytospora eugeniae BCC42696	*Arenga pinnata* (Petiole, Arecaceae)	Oil palm garden, Trang, Thailand	[144]
			Pleosporales sp. F46	*Mahonia fortune* (Pedicel, Berberidaceae)	Qingdao, China	[145]
			Scytalidium thermophilum YMF 1.04054	Soil	Hotspring National Park, Yunnan, China	[146]
			Cladosporium sp. JJM22	*Ceriops tagal* (Mangrove plant, Rhizophoraceae)	Dongzhaigang, Hainan, China	[65]
4(*S*)-(+)-Isosclerone acetate (**10**)	220	$C_{12}H_{12}O_4$	*Cytospora* sp.	*Ilex canariensis* (Aquifoliaceae)	Gomera, Spain	[60]
Xylarenone (**11**)	192	$C_{11}H_{12}O_3$	*Xylariaceous* sp. PSU-A80	*Garcinia atroviridis* (Leaves, Clusiaceae)	Songkhla Province, Thailand	[97]
			Xylariales sp. PSU-ES163	*Halophila ovalis* (Seagrass leaves, Hydrocharitaceae)	Trang, Thailand	[124]
(±)-Isosclerone (**12**)	178	$C_{10}H_{10}O_3$	*Guignardia laricina*	*Larix leptolepis* (Pinaceae)	Japan	[147]
			Unidentified freshwater fungus YMF 1.01029	Split of decaying branches of an unidentified tree	Near Lake Fuxian in Yunnan Province, China	[51]

			Botryosphaeria australis ZJ12-1A	*Sonneratia apetala* (Mangrove plant, Lythraceae)	Xiamen Haicang mangrove Conservation Area, Fujian, China	[74]
(3*S*,4*R*)-4,8-Dihydroxy-3-methoxy-3,4-dihydro-1(2*H*)-naphthalenone (**13**)	208	$C_{11}H_{12}O_4$	*Phaeosphaeria* sp.	*Bambusa vulgaris* (Decaying bamboo stem, Poaceae)	Queen Sirikit Botanic Garden, Chiang Mai, Thailand	[61]
3,8-Dihydroxy-3,4-dihydronaphthalen-1(2*H*)-one (= Vermelone) (**14**)	178	$C_{10}H_{10}O_3$	*Ceratocystis minor* (Hedgc.) Hunt	*Dendroctonus frontalis* (Insect, Scolytidae)	Southern USA	[118]
			Ceratocystis fimbriata coffea	*Coffea arabica* (Canker disease, Rubiaceae)	China	[41]
			Phaeosphaeria sp.	*Bambusa vulgaris* (Decaying bamboo stem, Poaceae)	Queen Sirikit Botanic Garden, Chiang Mai, Thailand	[61]
(4*S*)-4-Hydroxy-3,4-dihydronaphthalen-1(2*H*)-one (**15**)	162	$C_{10}H_{10}O_2$	*Didymella glomerata* X223	*Saussurea laniceps* (Roots, Asteraceae)	Tibet Kangbu hot, Yadong, Shigatse, Tibet, China	[148]
(4*S*)-4,6-Dihydroxy-3,4-dihydronaphthalen-1(2*H*)-one (**16**)	178	$C_{10}H_{10}O_3$	*Didymella glomerata* X223	*Saussurea laniceps* (Roots, Asteraceae)	Tibet Kangbu hot, Yadong, Shigatse, Tibet, China	[148]
(+)-Scytalone (= 3,6,8-Trihydroxytetralone = rac-Scytalone) (**17**)	194	$C_{10}H_{10}O_4$	*Scytalidium* sp.	Cultured	Japan	[57]
			Verticillium dahlia T9	Cultured	USA	[149]
			Verticillium dahlia brm-2 mutant	Cultured	USA	[119]
			Ceratocystis fimbriata coffea	*Coffea arabica* (Canker disease, Rubiaceae)	China	[82]
			Phaeoacremonium aleophilum (Pal) PVFi69-257	*Vitis vinifera* (Grapevine, Vitaceae)	Italy	[42]

			Lachnellula sp. A 32-89	*Polylepis sericea* (Bark, Rosaceae)	Laguna Negra, Venezuela	[35]
			Ceratocystis fimbriata f. sp. *platani*	Cultured	Madonna del Piano, Ticino, Switzerland	[39]
			Botrytis fabae Bf-CO-05	*Vicia faba* (Bean, Fabaceae)	Cordoba field, Institute for Sustainable Agriculture, CSIC, Cordoba, Spain	[44]
			Annulohypoxylon sp.	*Rhizophora racemosa* (Mangrove plant fruits, Rhizophoraceae)	Cameroon	[150]
			Raffaelea quercivora JCM 11526	*Platypus quercivorus* (Ambrosia beetle, Curculionidae)	Japan	[43]
(3R)-3,8-Dihydroxy-6-methoxy-tetralone (**18**)	208	$C_{11}H_{12}O_4$	*Phomopsis* sp. sh917	*Isodon eriocalyx* var. *laxiflora* (Stem, Lamiaceae)	Kunming Botanical Garden, Kunming, China,	[85]
cis-4-Hydroxyscytalone (= 3*S*,4*R*-Hydoxy-scytalone) (**19**)	210	$C_{10}H_{10}O_5$	*Raffaelea quercivora* JCM 11526	*Platypus quercivorus* (Ambrosia beetle, Curculionidae)	Japan	[43]
	210	$C_{10}H_{10}O_5$	*Mycosphaerella fijiensis* IMI 105378	*Musa sapientum* (Musaceae)	San Pedro Sula, Honduras	[40]
			Ceratocystis fimbriata coffea	*Coffea arabica* (Canker disease, Rubiaceae)	China	[41]
			Ceratocystis fimbriata f. sp. *platani*	Cultured	Madonna del Piano, Ticino, Switzerland	[39]
			Caryospora callicarpa YMF1.01026	Submerged woody substrate, freshwater	Yunnan Province, China	[52]
			Daldinia eschscholzii	*Mantis religiosa* (Mantis, Mantidae)	China	[27]
			Alternaria sp. A744	*Morinda officinalis* (Twigs, Rubiaceae)	Gaoyao city, Guangdong, China	[82]

(±)-Scytalone (= 3,6,8-Trihydroxytetralone) (**20**)	194	$C_{10}H_{10}O_4$	*Xylariaceae* sp. SCSGAF0086	*Melitodes squamata* (Gorgonian coral, Melithaeinae)	South China Sea, China	[134]
6-Hydroxyisosclerone (**21**)	194	$C_{10}H_{10}O_4$	*Tubakia dryina* (Sacc.) PV-6	East of the Mississippi River	USA	[63]
			Aureobasidium pullulans	*Aloe vera* (Leaves, Asphodelaceae)	Marrakesh, Morroco	[84]
			Alternaria sp. A744	*Morinda officinalis* (Twigs, Rubiaceae)	Gaoyao city, Guangdong, China	[82]
			Paraphoma sp. HR-12-1	Desert	Beijing, china	[87]
4,6,8-Trihydroxy-3,4-dihydronaphthalen-1(2*H*)-one (**22**)	194	$C_{10}H_{10}O_4$	*Pyricularia oryzae* Cav. (Ken 53-33)	Rice blast	Japan	[129]
			Sclerotinia sclerotiorum (LIB.) DE BARY.	Cultured	Japan	[37]
			Daldinia eschscholzii	*Mantis religiosa* (Mantis, Mantidae)	China	[27]
			Ceratocystis minor (Hedgc.) Hunt	*Dendroctonus frontalis* (Insect, Scolytidae)	Southern USA	[118]
			Caryospora callicarpa YMF1.01026	Submerged woody substrate, freshwater	Yunnan Province, China	[52]
			Phaeosphaeria sp.	*Bambusa vulgaris* (Decaying bamboo stem, Poaceae)	Queen Sirikit Botanic Garden, Chiang Mai, Thailand	[61]
			Unidentified freshwater fungus YMF 1.01029	Split of decaying branches of an unidentified tree	Near Lake Fuxian in Yunnan Province, China	[51]
			Thermomyces lanuginosus YMF 1.04048	Soil	Hotspring National Park, Yunnan, China	[146]
Didymelol A (**23**)	178	$C_{10}H_{10}O_3$	*Didymella glomerata* X223	*Saussurea laniceps* (Roots, Asteraceae)	Tibet Kangbu hot, Yadong, Shigatse, Tibet, China	[148]

(3*S*,4*R*)-3,4,6-Trihydroxy-3,4 dihydronaphthalen-1(2*H*)-one (**24**)	194	$C_{10}H_{10}O_4$	*Didymella glomerata* X223	*Saussurea laniceps* (Roots, Asteraceae)	Tibet Kangbu hot, Yadong, Shigatse, Tibet, China	[148]
(*R*)-4,6,8-trihydroxy-3,4-dihydro-1(2*H*)-naphthalenone (**25**)	194	$C_{10}H_{10}O_4$	*Xylariaceae* sp. CR1546C	*Sticta fuliginosa* (Lichen, Lobariaceae)	Costa Rica	[63]
			Xylaria sp. SYPF 8246	*Panax notoginseng*, roots (Araliaceae)	Wenshan, Yunnan, China	[151]
(3*R*,4*R*)-3,4-Dihydro-3,4,6,8-tetrahydroxy-1(2*H*)-naphthalenone (**26**)	210	$C_{10}H_{10}O_5$	*Xylariales* sp. PSU-ES163	*Halophila ovalis* (Seagrass leaves, Hydrocharitaceae)	Trang, Thailand	[124]
			Thermomyces lanuginosus YMF 1.04048	Soil	Hotspring National Park, Yunnan, China	[146]
3,4,8-Trihydroxy-6-(hydroxymethyl)-3,4-dihydronaphthalen-1(2*H*)-one (**27**)	224	$C_{11}H_{12}O_5$	*Daldinia eschscholzii*	*Mantis religiosa* (Mantis, Mantidae)	China	[27]
3,4,6,8-Tetrahydroxy-3,4-dihydronaphthalen-1(2*H*)-one (**28**)	210	$C_{10}H_{10}O_5$	*Sclerotinia sclerotiorum* (LIB.) DE BARY.	Cultured	Japan	[37]
			Ceratocystis minor (Hedgc.) Hunt	*Dendroctonus frontalis* (Insect, Scolytidae)	Southern USA	[118]
			Ceratocystis fimbriata f. sp. *platani*	Cultured	Madonna del Piano, Ticino, Switzerland	[39]
			Phaeosphaeria sp.	*Bambusa vulgaris* (Decaying bamboo stem, Poaceae)	Queen Sirikit Botanic Garden, Chiang Mai, Thailand	[61]
			Annulohypoxylon sp.	*Rhizophora racemosa* (Mangrove plant fruits, Rhizophoraceae)	Cameroon	[150]
Phomopsiketone C (**29**)	224	$C_{11}H_{12}O_5$	*Phomopsis* sp. sh917	*Isodon eriocalyx* var. *laxiflora* (Stem, Lamiaceae)	Kunming Botanical Garden, Kunming, China,	[85]
(3*R*,4*R*)-3,4,8-trihydroxy-6-methoxy-3,4-dihydro-1(2*H*)-naphthalenone (**30**)	224	$C_{11}H_{12}O_5$	*Pleosporales* sp. F46	*Mahonia fortune* (Pedicel, Berberidaceae)	Qingdao, China.	[145]

(3*S*,4*S*)-3,4,8-Trihydroxy-6-methoxy-3,4-dihydro-1(2*H*)-naphthalenone (**31**)	224	$C_{11}H_{12}O_5$	*Phaeosphaeria* sp.	*Bambusa vulgaris* (Decaying bamboo stem, Poaceae)	Queen Sirikit Botanic Garden, Chiang Mai, Thailand	[61]
			Neofusicoccum austral BL24 (haplotype H1)	*Phoenicean juniper* (Symptomatic branch, Cupressaceae)	Vatural area on Caprera Island, Italy	[45]
(3*S*,4*S*)-4-Hydroxyscytalone (**32**)	210	$C_{10}H_{10}O_5$	*Penicillium* sp. KMM 4672	*Padina* sp. (Brown alga, Dictyotaceae)	Van Phong Bay, South China Sea, Vietnam	[113]
2,4,6,8-Tetrahydroxy-3,4-dihydronaphthalen-1(2*H*)-one (**33**)	210	$C_{10}H_{10}O_5$	*Sclerotinia sclerotiorum* (LIB.) DE BARY	Cultured	Japan	[37]
Teratosphaerone B (**34**)	222	$C_{12}H_{14}O_4$	*Teratosphaeria* sp. FL2137	*Pinus clausa* (Leaves, Pinaceae)	The ground of a pine dominated scrub forest, Archbold Biological Station in Florida, USA	[152]
(3*S*,4*R*)-(+)-3,4,5-Trihydroxy-1-tetralone (= 4-Hydroxy-6-deoxyscytalone) (**35**)	194	$C_{10}H_{10}O_4$	*Xylariaceae* sp. SCSGAF0086	*Melitodes squamata* (Gorgonian coral, Melithaeinae)	South China Sea, China	[134]
(4*S*)-5-Hydroxy-4-methoxy-α-tetralone (**36**)	192	$C_{11}H_{12}O_3$	*Cytospora eugeniae* BCC42696	*Arenga pinnata* (Petiole, Arecaceae)	Oil palm garden, Trang, Thailand	[144]
(4*R*)-(-)-5-*O*-Methylsclerone (**37**)	192	$C_{11}H_{12}O_3$	*Xylariaceae* sp. SCSGAF0086	*Melitodes squamata* (Gorgonian coral, Melithaeinae)	South China Sea, China	[134]
(*R*)-4,5-Dihydroxy-3,4-dihydronaphthalen-1(2H)-one (**38**)	178	$C_{10}H_{10}O_3$	*Phialocephala fortinii*	*Pamelia* sp. (Lichen, Parmeliaceae)	Mount Qingliang, Zhejiang, China	[137]
(4*R*)-(-)-5-Hydroxy-4-methoxy-1-tetralone (**39**)	192	$C_{11}H_{12}O_3$	*Xylariaceae* sp. SCSGAF0086	*Melitodes squamata* (Gorgonian coral, Melithaeinae)	South China Sea, China	[134]
(3*S**,4*S**)-3,4,5-trihydroxy-1-tetralone (**40**)	194	$C_{10}H_{10}O_4$	*Neofusicoccum parvum* B19	*Vitis vinifera* (Declining vines of grapevine, Vitaceae)	Inzolia, Sicily	[46]
			Daldinia eschscholtzii	*Paphiopedilum exul* (Orchid roots, Orchidaceae)	Chiang Mai, Thailand	[21]

3,4-Dihydro-3,4,5-trihydroxy-1(2*H*)-naphthalenone (**41**)	194	$C_{10}H_{10}O_4$	*Hypoxylon mammarum*	*Populus tremula* (Canker margin of an infected quaking aspen, Salicaceae)	France	[121]
Botrytone (**42**)	194	$C_{10}H_{10}O_4$	*Botrytis fabae* Bf-CO-05	*Vicia faba* (Bean, Fabaceae)	Cordoba field, Institute for Sustainable Agriculture, CSIC, Cordoba, Spain	[44]
(3S)-3,8-Dihydroxy-6,7-dimethyl-α-tetralone (**43**)	206	$C_{12}H_{14}O_3$	*Daldinia eschscholtzii* PSUSTD57	*Bruguiera gymnorrhiza* (Mangrove plant leaves, Rhizophoraceae)	Suratthani, Thailand	[64]
			Cladosporium sp. JJM22	*Ceriops tagal* (Rhizophoraceae)	South China Sea, Dongzhaigang, Hainan, China	[65]
			Cladosporium sp. JJM22	*Ceriops tagal* (Mangrove plant, Rhizophoraceae)	Dongzhaigang, Hainan, China	[65]
(-)-cis-(3R*,4S*)-3,4,8-Trihydroxy-6,7-dimethyl-3,4-dihydro-1(2H)-naphthalenone (**44**)	222	$C_{12}H_{14}O_4$	*Nodulisporium* sp.	*Antidesma ghaesembilla* (Fresh twigs, Phyllanthaceae)	Chiang Mai, Thailand	[66]
Nodulone C (**45**)	222	$C_{12}H_{14}O_4$	*Daldinia eschscholtzii*	*Paphiopedilum exul* (Orchid roots, Orchidaceae)	Chiang Mai, Thailand	[21]
Asparvenone (**46**)	224	$C_{11}H_{12}O_5$	*Aspergillus parvulus*	Cultured	USA	[153]
			Aspergillus sparvulus FB6036	Cultured	Japan	[102]
6-*O*-Methylasparvenone (**47**)	238	$C_{12}H_{14}O_5$	*Aspergillus parvulus*	Cultured	USA	[153]
			Lachnum papyraceum A48-88 (Karst.) Karst	Cultured	Germany	[44]
			Aspergillus sparvulus FB6036	Cultured	Japan	[102]
			Neofusicoccum austral SYSU-SKS024	*Kandelia candel* (Mangrove plant branches, Rhizophoraceae)	Shankou, Guangxi, China	[107]
3,4-Dihydro-4,8-dihydroxy-6-methoxy-7-methyl-1(2*H*)-naphthalenone (**48**)	222	$C_{12}H_{14}O_4$	*Lachnum papyraceum* A48-88 (Karst.) Karst (Hyaloscyphaceae)	Cultured	Germany	[44]

(3*R**,4*S**)-3,4-Dihydroxy-7-methyl-3,4-dihydro-1(2*H*)-naphthalenone (**49**)	192	$C_{11}H_{12}O_3$	*Acremonium roseogriseum* (S. B. Saksena) W. Gams	*Cladostephus spongius* (Brown alga)	Mediterranean Sea, Spanish coast, Moraira	[98]
(3*S**,4*S**)-3,4-Dihydroxy-7-methyl-3,4-dihydro-1(2*H*)-naphthalenone (**50**)	192	$C_{11}H_{12}O_3$	*Acremonium roseogriseum* (S. B. Saksena) W. Gams	*Cladostephus spongius* (Brown alga)	Mediterranean Sea, Spanish coast, Moraira	[98]
Corynenone A (**51**)	240	$C_{11}H_{12}O_6$	*Corynespora cassiicola* XS-20090I7	Unidentified sponge (XS-2009001)	Xisha Islands coral reef, South China Sea, China	[114]
Corynenone B (**52**)	240	$C_{11}H_{12}O_6$	*Corynespora cassiicola* XS-20090I7	Unidentified sponge (XS-2009001)	Xisha Islands coral reef, South China Sea, China	[114]
Botryosphaerone D (**53**)	252	$C_{13}H_{16}O_5$	*Botryosphaeria australis* ZJ12-1A	*Sonneratia apetala* (Mangrove plant, Lythraceae)	Xiamen Haicang mangrove Conservation Area, Fujian, China	[74]
			Neofusicoccum austral BL24 (haplotype H1)	*Phoenicean juniper* (Symptomatic branch, Cupressaceae)	Vatural area on Caprera Island, Italy	[45]
			Neofusicoccum austral SYSU-SKS024	*Kandelia candel* (Mangrove plant branches, Rhizophoraceae)	Shankou, Guangxi, China	[107]
(3*R*,4*R*)-3-Methoxyl-botryosphaerone D (**54**)	266	$C_{14}H_{18}O_5$	*Neofusicoccum austral* SYSU-SKS024	*Kandelia candel* (Mangrove plant branches, Rhizophoraceae)	Shankou, Guangxi, China	[107]
Botryosphaerone E (**55**)	266	$C_{14}H_{18}O_5$	*Pyrenochaetopsis* sp. MSX63693	Cultured	USA	[86]
4,8-Dihydroxy-7-(2-hydroxy-ethyl)-6-methoxy-3,4-dihydro-2*H*-naphthalen-1-one (**56**)	252	$C_{13}H_{16}O_5$	Mycelia sterilia	Sponge	China	[154]
(-)-4,8-Dihydroxy-7-(2-hydroxy-ethyl)-6-methoxy-3,4-dihydro-2*H*-naphthalen-1-one (**57**)	252	$C_{13}H_{16}O_5$	*Leptosphaerulina chartarum* 3608	Crinoid (Marine animal, Crinoidea)	Xuwen, Zhanjiang City, Guangdong, China	[91]
(+)-10-Norparvulenone (**58**)	236	$C_{13}H_{16}O_4$	*Leptosphaerulina chartarum* 3608	Crinoid (Marine animal, Crinoidea)	Xuwen, Zhanjiang City,	[91]

					Guangdong, China	
4,6,8-Trihydroxy-5-methyl-3,4-dihydronaphthalen-1(2H)-one (**59**)	208	$C_{11}H_{12}O_4$	*Paraphoma* sp. HR-12-1	Desert	Beijing, china	[87]
3*S**,4*S**-7-Ethyl-4,8-dihydroxy-3,6-dimethoxy-3,4-dihydronaphthalen-1(2*H*)-one (**60**)	266	$C_{14}H_{18}O_5$	*Delitschia* sp. JF16003	Submerged wood	Ariége, Rimont, France	[155]
(3*R**,4*S**)-7-Ethyl-3,4,6,8-tetrahydroxy-3,4-dihydronaphthalen-1(2*H*)-one (**61**)	238	$C_{12}H_{14}O_5$	*Delitschia corticola*	Submerged wood	Freshwater habitat, Yunnan, China	[67]
(-)-*trans*-(3*R*,4*R*)-3,4,8-Trihydroxy-6,7-dimethyl-3,4- dihydronaphthalen-1(2*H*)-one (**62**)	222	$C_{12}H_{14}O_4$	*Cladosporium sp.* JJM22	*Ceriops tagal* (Mangrove plant, Rhizophoraceae)	Dongzhaigang, Hainan, China	[65]
			Cladosporium sp. JJM22	*Ceriops tagal* (Rhizophoraceae)	South China Sea, Dongzhaigang, Hainan, China	[65]
Nodulone (**63**)	236	$C_{12}H_{12}O_5$	*Nodulisporium* sp. A4	*Aquilaria sinensis* (Stems, Thymelaeaceae)	Xinyi, Guangdong Province, China	[156]
			Daldinia eschscholzii	*Mantis religiosa* (Mantis, Mantidae)	China	[27]
			Daldinia eschscholtzii	*Paphiopedilum exul* (Orchid roots, Orchidaceae)	Chiang Mai, Thailand	[21]
(-)-*cis*-(7*R**,8*S**)-4,7,8-Trihydroxy-3,6,7,8-tetrahydronaphtho[2,3-*c*]furan-5(1*H*)-one (**64**)	236	$C_{12}H_{12}O_5$	*Nodulisporium* sp.	*Antidesma ghaesembilla*, (Fresh twigs, Phyllanthaceae)	Chiang Mai, Thailand	[66]
Nodulone B (**65**)	220	$C_{12}H_{12}O_4$	*Daldinia eschscholtzii*	*Paphiopedilum exul* (Orchid roots, Orchidaceae)	Chiang Mai, Thailand	[21]
3,4-Dihydro-3-(2(ξ)-hydroxypropyl)-3(ξ),6,8-trihydroxy-1(2*H*)-naphthalenone (**66**)	252	$C_{13}H_{16}O_5$	*Botryosphaeria* sp. BCC 8200	*Musa* sp. (Musaceae)	Doi Suthep-Pui National Park, Chiang Mai Province, Thailand	[68]
3,4-Dihydro-3-(2-oxo-propyl)-3(ξ),6,8-trihydroxy-1(2*H*)-naphthalenone (**67**)	250	$C_{13}H_{14}O_5$	*Botryosphaeria* sp. BCC 8200	*Musa* sp. (Musaceae)	Doi Suthep-Pui National Park, Chiang Mai	[68]

					Province, Thailand	
3,6,8-Trihydroxy-3-[3,5-dimethyl-2-oxo-3(*E*)-heptenyl]-2,3-dihydronaphthalen-1(4*H*)-one (**68**)	332	$C_{19}H_{24}O_5$	*Keissleriella* sp. Y4108	Marine sediment	Yellow Sea, Sheyang port, China	[69]
3,4-Dihydro-3-(4(ξ)-hydroxy-2-oxo-pentyl)-3(ξ),6,8-trihydroxy-1(2*H*)-naphthalenone (**69**)	294	$C_{15}H_{18}O_6$	*Botryosphaeria* sp. BCC 8200	*Musa* sp. (Musaceae)	Doi Suthep-Pui National Park, Chiang Mai Province, Thailand	[68]
Scytalol A (**70**)	294	$C_{15}H_{18}O_6$	*Scytalidium* sp. 36-93	Infected body of *Basidiomycete* growing on wood	Germany	[105]
Scytalol B (**71**)	308	$C_{16}H_{20}O_6$	*Scytalidium* sp. 36-93	Infected body of *Basidiomycete* growing on wood	Germany	[105]
Scytalol C (**72**)	320	$C_{17}H_{20}O_6$	*Scytalidium* sp. 36-93	Infected body of *Basidiomycete* growing on wood	Germany	[105]
			Pleosporales strain 222	Driftwood	Coast of the Greifswalder Bodden, Baltic Sea, Germany	[70]
			Biatriospora sp. 8331C	*Pseudosyphellaria* sp. (Lichen, Lobariaceae)	Changbai Mountain, Jilin, China	[157]
Scytalol D (**73**)	264	$C_{14}H_{16}O_5$	*Scytalidium* sp. 36-93	Infected body of a *Basidiomycete* growing on wood	Germany	[105]
			Biatriospora sp. 8331C	*Pseudosyphellaria* sp. (Lichen, Lobariaceae)	Changbai Mountain, Jilin, China	[157]
Balticol A (**74**)	264	$C_{14}H_{16}O_5$	*Pleosporales* strain 222,	Driftwood	Coast of the Greifswalder Bodden, Baltic Sea, Germany	[70]

			Biatriospora sp. 8331C	*Pseudosyphellaria* sp. (Lichen, Lobariaceae)	Changbai Mountain, Jilin, China	[157]
			Pleosporales sp. F46	*Mahonia fortune* (Pedicel, Berberidaceae)	Qingdao, China	[145]
Balticol B (**75**)	238	$C_{12}H_{14}O_5$	*Pleosporales* strain 222	Driftwood	Coast of the Greifswalder Bodden, Baltic Sea, Germany	[70]
			Pleosporales sp. F46	*Mahonia fortune* (Pedicel, Berberidaceae)	Qingdao, China	[145]
Iso-Balticol B-4,9-acetonide (**76**)	278	$C_{15}H_{18}O_5$	*Teratosphaeria* sp. FL2137	*Pinus clausa* (Leaves, Pinaceae)	The ground of a pine dominated scrub forest, Archbold Biological Station in Florida, USA	[152]
Iso-Balticol B (**77**)	238	$C_{12}H_{14}O_5$	*Teratosphaeria* sp. FL2137	*Pinus clausa* (Leaves, Pinaceae)	The ground of a pine-dominated scrub forest, Archbold Biological Station in Florida, USA	[152]
Balticol C (**78**)	254	$C_{12}H_{14}O_6$	*Pleosporales* strain 222	Driftwood	Coast of the Greifswalder Bodden, Baltic Sea, Germany	[70]
(+)-Balticol C (**79**)	254	$C_{12}H_{14}O_6$	*Teratosphaeria* sp. FL2137	*Pinus clausa* (Leaves, Pinaceae)	The ground of a pine-dominated scrub forest, Archbold Biological	[152]

					Station in Florida, USA	
Balticol D (**80**)	280	$C_{14}H_{16}O_6$	*Pleosporales* strain 222	Driftwood	Coast of the Greifswalder Bodden, Baltic Sea, Germany	[70]
Balticol E (**81**)	282	$C_{14}H_{18}O_6$	*Pleosporales* strain 222	Driftwood	Coast of the Greifswalder Bodden, Baltic Sea, Germany	[70]
			Biatriospora sp. 8331C	*Pseudosyphellaria* sp. (Lichen, Lobariaceae)	Changbai Mountain, Jilin, China	[157]
Balticol F (**82**)	294	$C_{15}H_{18}O_6$	*Pleosporales* strain 222	Driftwood	Coast of the Greifswalder Bodden, Baltic Sea, Germany	[70]
			Biatriospora sp. 8331C	*Pseudosyphellaria* sp. (Lichen, Lobariaceae)	Changbai Mountain, Jilin, China	[157]
Fusaranthraquinone (**83**)	324	$C_{16}H_{20}O_7$	*Fusarium* spp. PSU-F14 and PSU-F135	*Annella* sp. (Gorgonian Sea fan, Gorgoniidae)	Koh Hin Ran Pet, Suratthani, Thailand	[72]
9α-Hydroxydihydrodesoxybostrycin (**84**)	324	$C_{16}H_{20}O_7$	PSU-N24	*Garcinia nigrolineata* (Branch, Clusiaceae)	Ton Nga Chang wildlife sanctuary, Songkhla, southern Thailand	[71]
9α-Hydroxyhalorosellinia A (**85**)	340	$C_{16}H_{20}O_8$	PSU-N24	*Garcinia nigrolineata* (Branch, Clusiaceae)	Ton Nga Chang wildlife sanctuary, Songkhla,	[71]

					southern Thailand	
			Fusarium spp. PSU-F14 and PSU-F135	*Annella* sp. (Gorgonian Sea fan, Gorgoniidae)	Koh Hin Ran Pet, Suratthani, Thailand	[72]
Fusarnaphthoquinone A (**86**)	310	$C_{15}H_{18}O_7$	*Fusarium* spp. PSU-F14 and PSU-F135	*Annella* sp. (Gorgonian Sea fan, Gorgoniidae)	Koh Hin Ran Pet, Suratthani, Thailand	[72]
Fusarnaphthoquinone B (**87**)	276	$C_{15}H_{16}O_5$	*Fusarium* spp. PSU-F14 and PSU-F135	*Annella* sp. (Gorgonian Sea fan, Gorgoniidae)	Koh Hin Ran Pet, Suratthani, Thailand	[72]
			Fusarium spp. PSU-F14 and PSU-F135	*Annella* sp. (Gorgonian Sea fan, Gorgoniidae)	Koh Hin Ran Pet, Suratthani, Thailand	[72]
3-Demethoxyl-fusarnaphthoquinone B (**88**)	246	$C_{14}H_{14}O_4$	*Fusarium* sp. HP-2	*Aquilaria malaccensis* (Agarwood, Thymelaeaceae)	Ledong County, Hainan, China	[108]
9*S*-Hydroxydihydrodesoxybostrycin (**89**)	324	$C_{16}H_{20}O_7$	PSU-N24	*Garcinia nigrolineata* (Branch, Clusiaceae)	Ton Nga Chang wildlife sanctuary, Songkhla, southern Thailand	[71]
Perenniporide A (**90**)	334	$C_{17}H_{18}O_7$	*Perenniporia* sp.	*Euops chinesis* (Leaf-rolling weevil larva, Attelabidae)	Mingyue Mountain, Jiangxi, China	[73]
			Neofusicoccum parvum B19	*Vitis vinifera* (Declining vines of grapevine, Vitaceae)	Inzolia, Sicily	[46]
Perenniporide B (**91**)	366	$C_{18}H_{22}O_8$	*Perenniporia* sp.	*Euops chinesis* (Leaf-rolling weevil larva, Attelabidae)	Mingyue Mountain, Jiangxi, China	[73]
Perenniporide C (**92**)	366	$C_{18}H_{22}O_8$	*Perenniporia* sp.	*Euops chinesis* (Leaf-rolling weevil larva, Attelabidae)	Mingyue Mountain, Jiangxi, China	[73]

Perenniporide D (**93**)	352	$C_{17}H_{20}O_8$	*Perenniporia* sp.	*Euops chinesis* (Leaf-rolling weevil larva, Attelabidae)	Mingyue Mountain, Jiangxi, China	[73]
			Neofusicoccum parvum B19	*Vitis vinifera* (Declining vines of grapevine, Vitaceae)	Inzolia, Sicily	[46]
Botryosphaerone A (**94**)	268	$C_{13}H_{16}O_6$	*Botryosphaeria australis* ZJ12-1A	*Sonneratia apetala* (Mangrove plant, Lythraceae)	Xiamen Haicang mangrove Conservation Area, Fujian, China	[74]
Botryosphaerone B (**95**)	268	$C_{13}H_{16}O_6$	*Botryosphaeria australis* ZJ12-1A	*Sonneratia apetala* (Mangrove plant, Lythraceae)	Xiamen Haicang mangrove Conservation Area, Fujian, China	[74]
			Neofusicoccum austral SYSU-SKS024	*Kandelia candel* (Mangrove plant branches, Rhizophoraceae)	Shankou, Guangxi, China	[107]
Botryosphaerone C (**96**)	282	$C_{14}H_{18}O_6$	*Botryosphaeria australis* ZJ12-1A	*Sonneratia apetala* (Mangrove plant, Lythraceae)	Xiamen Haicang mangrove Conservation Area, Fujian, China	[74]
(2*S*,3*S*,4*S*)-8-Dehydroxy-8-methoxyl-dihydronaphthalenone (**97**)	306	$C_{16}H_{18}O_6$	*Fusarium* sp. HP-2	*Aquilaria malaccensis* (Agarwood, Thymelaeaceae)	Ledong County, Hainan, China	[108]
Dihydronaphthalenone (2*S*,3*S*,4*S*) (**98**)	292	$C_{15}H_{16}O_6$	*Fusarium* sp. BCC14842	*Bambusa vulgaris* (Bamboo leaf, Poaceae)	Bamboo forest at Nam Nao National Park, Phetchabun Province, Thailand.	[75]
			Fusarium sp. HP-2	*Aquilaria malaccensis* (Agarwood, Thymelaeaceae)	Ledong County, Hainan, China	[108]
			Fusarium solani B-18	Inner tissue of the unidentified forest litters	Merapi Mt. area, Sleman,	[158]

					Yogyakarta, Indonesia	
Diastereomer-Dihydronaphthalenone B (2*R*,3*S*,4*R*) (**99**)	292	$C_{15}H_{16}O_6$	*Fusarium* sp. BCC14842	*Bambusa vulgaris* (Bamboo leaf, Poaceae)	Bamboo forest at Nam Nao National Park, Phetchabun Province, Thailand.	[75]
			Fusarium solani B-18	Inner tissue of the unidentified forest litters	Merapi Mt. area, Sleman, Yogyakarta, Indonesia	[158]
			Fusarium solani H918	Mangrove sediments	Zhangjiangkou Mangrove National Nature Reserve, Fujian, China	[159]
(3a*S*,9*R*,9a*S*)-1(9a),3(3a),9-Hexahydromonosporascone (**100**)	250	$C_{13}H_{14}O_5$	*Teratosphaeria* sp. FL2137	*Pinus clausa* (Leaves, Pinaceae)	The ground of a pine-dominated scrub forest, Archbold Biological Station in Florida, USA	[152]
(+)-4,8-Dihydroxy-7-(2-hydroxy-ethyl)-6-methoxy-3,4-dihydro-2*H*-naphthalen-1-one (**101**)	252	$C_{13}H_{16}O_5$	*Leptosphaerulina chartarum* 3608	Crinoid (Marine animal, Crinoidea)	Xuwen, Zhanjiang City, Guangdong, China	[91]
(-)-Leptothalenone A (**102**)	252	$C_{13}H_{16}O_5$	*Leptosphaerulina chartarum* 3608	Crinoid (Marine animal, Crinoidea)	Xuwen, Zhanjiang City, Guangdong, China	[91]
(+)-Leptothalenone A (**103**)	252	$C_{13}H_{16}O_5$	*Leptosphaerulina chartarum* 3608	Crinoid (Marine animal, Crinoidea)	Xuwen, Zhanjiang City,	[91]

					Guangdong, China	
4-Hydroxydihydronorjavanicin (**104**)	280	$C_{14}H_{16}O_6$	*Fusarium* sp. BCC14842	*Bambusa vulgaris* (Bamboo leaf, Poaceae)	Bamboo forest at Nam Nao National Park, Phetchabun Province, Thailand.	[75]
5-Hydroxydihydrofusarubin (**105**)	310	$C_{15}H_{18}O_7$	*Fusarium solani* T-127	Cultured	Japan	[160]
5-Hydroxydihydrofusarubin A (**106**)	310	$C_{15}H_{18}O_7$	*Fusarium* sp. BCC14842	*Bambusa vulgaris* (Bamboo leaf, Poaceae)	Bamboo forest at Nam Nao National Park, Phetchabun Province, Thailand.	[75]
5-Hydroxy-3-Methoxydihydrofusarubin A (**107**)	324	$C_{16}H_{20}O_7$	*Fusarium* sp. BCC14842	*Bambusa vulgaris* (Bamboo leaf, Poaceae)	Bamboo forest at Nam Nao National Park, Phetchabun Province, Thailand	[75]
5-Hydroxydihydrofusarubin D (**108**)	310	$C_{15}H_{18}O_7$	*Fusarium* sp. BCC14842	*Bambusa vulgaris* (Bamboo leaf, Poaceae)	Bamboo forest at Nam Nao National Park, Phetchabun Province, Thailand.	[75]
			Penicillium polonicum	*Camptotheca acuminate*, roots (Nyssaceae)	South Central University for Nationalities, Wuhan, Hubei, China	[161]
5-Hydroxy-3-methoxydihydrofusarubin D (**109**)	324	$C_{16}H_{20}O_7$	*Fusarium* sp. BCC14842	*Bambusa vulgaris* (Bamboo leaf, Poaceae)	Bamboo forest at Nam Nao	[75]

					National Park, Phetchabun Province, Thailand.	
3,5-Dimethoxydihydrofusarubin D (**110**)	338	$C_{17}H_{22}O_7$	*Fusarium* sp. BCC14842	*Bambusa vulgaris* (Bamboo leaf, Poaceae)	Bamboo forest at Nam Nao National Park, Phetchabun Province, Thailand.	[75]
5-Hydroxydihydrofusarubin B (**111**)	310	$C_{15}H_{18}O_7$	*Fusarium* sp. BCC14842	*Bambusa vulgaris* (Bamboo leaf, Poaceae)	Bamboo forest at Nam Nao National Park, Phetchabun Province, Thailand.	[75]
5-Methoxydihydrofusarubin B (**112**)	324	$C_{16}H_{20}O_7$	*Fusarium* sp. BCC14842	*Bambusa vulgaris* (Bamboo leaf, Poaceae)	Bamboo forest at Nam Nao National Park, Phetchabun Province, Thailand.	[75]
3,5-Dimethoxydihydrofusarubin B (**113**)	338	$C_{17}H_{22}O_7$	*Fusarium* sp. BCC14842	*Bambusa vulgaris* (Bamboo leaf, Poaceae)	Bamboo forest at Nam Nao National Park, Phetchabun Province, Thailand.	[75]
5-Hydroxydihydrofusarubin C (**114**)	310	$C_{15}H_{18}O_7$	*Fusarium* sp. BCC14842	*Bambusa vulgaris* (Bamboo leaf, Poaceae)	Bamboo forest at Nam Nao National Park, Phetchabun Province, Thailand.	[75]

Lentiquinone B (**115**)	292	$C_{15}H_{16}O_6$	*Ascochyta lentis*	*Lens culinaris* (Lentil, Fabaceae)	USA	[47]
Lentiquinone C (**116**)	292	$C_{15}H_{16}O_6$	*Ascochyta lentis*	*Lens culinaris* (Lentil, Fabaceae)	USA	[47]
Pleosporalin H (**117**)	282	$C_{14}H_{18}O_6$	*Pleosporales* sp. F46	*Mahonia fortune* (Pedicel, Berberidaceae)	Qingdao, China	[145]
Pleosporalin I (**118**)	250	$C_{13}H_{14}O_5$	*Pleosporales* sp. F46	*Mahonia fortune* (Pedicel, Berberidaceae)	Qingdao, China	[145]
Biatriosporin A (**119**)	280	$C_{15}H_{20}O_5$	*Biatriospora* sp. 8331C	*Pseudosyphellaria* sp. (Lichen, Lobariaceae)	Changbai Mountain, Jilin, China	[157]
Biatriosporin B (**120**)	266	$C_{14}H_{18}O_5$	*Biatriospora* sp. 8331C	*Pseudosyphellaria* sp. (Lichen, Lobariaceae)	Changbai Mountain, Jilin, China	[157]
Biatriosporin C (**121**)	266	$C_{14}H_{18}O_5$	*Biatriospora* sp. 8331C	*Pseudosyphellaria* sp. (Lichen, Lobariaceae)	Changbai Mountain, Jilin, China	[157]
Biatriosporin G (**122**)	308	$C_{16}H_{20}O_6$	*Biatriospora* sp. 8331C	*Pseudosyphellaria* sp. (Lichen, Lobariaceae)	Changbai Mountain, Jilin, China	[157]
Biatriosporin H (**123**)	308	$C_{16}H_{20}O_6$	*Biatriospora* sp. 8331C	*Pseudosyphellaria* sp. (Lichen, Lobariaceae)	Changbai Mountain, Jilin, China	[157]
Biatriosporin I (**124**)	278	$C_{15}H_{18}O_5$	*Biatriospora* sp. 8331C	*Pseudosyphellaria* sp. (Lichen, Lobariaceae)	Changbai Mountain, Jilin, China	[157]
Biatriosporin J (**125**)	276	$C_{15}H_{16}O_5$	*Biatriospora* sp. 8331C	*Pseudosyphellaria* sp. (Lichen, Lobariaceae)	Changbai Mountain, Jilin, China	[157]
Pleorubrin B (**126**)	278	$C_{15}H_{18}O_5$	*Biatriospora* sp. CCF 4378	*Ulmus laevis* (Twigs, Ulmaceae)	Libicky Luh Forest near Velky Osek, Czech	[162]
Aspvanicin A (**127**)	294	$C_{15}H_{18}O_6$	*Aspergillus versicolor* KU258497	*Eichhornia crassipes* (Leaves, Pontederiaceae)	Shores of the River Nile in Mansoura, Egypt	[89]

Aspvanicin B (**128**)	294	$C_{15}H_{18}O_6$	*Aspergillus versicolor* KU258497	*Eichhornia crassipes* (Leaves, Pontederiaceae)	Shores of the River Nile in Mansoura, Egypt	[89]
Methylberchemiaside (**129**)	354	$C_{17}H_{22}O_8$	*Colletotrichum* sp. GDMU-1	*Santalum album* (Leaves, Santalaceae)	Dongguan, Guangdong, China	[112]
Phomonaphthalenone A (**130**)	292	$C_{15}H_{16}O_6$	*Phomopsis sp.* HCCB04730	*Stephaniae Japonicae* (Stems, Menispermaceae)	Jin Hua, Zhejiang, China	[90]
Daldionin (**131**)	318	$C_{20}H_{14}O_4$	*Daldinia eschscholtzii*	*Paphiopedilum exul* (Orchid roots, Orchidaceae)	Chiang Mai, Thailand	[21]
6-Ethyl-l-acetonyl-l,5-dihydroxy-2,7-dimethoxy-4-naphthalenone (**132**)	320	$C_{17}H_{20}O_6$	*Guignardia laricina*	*Larix leptolepis* (Pinaceae)	Japan	[48]
			Perenniporia sp.	*Euops chinesis* (Leaf-rolling weevil larva, Attelabidae)	Mingyue Mountain, Jiangxi, China	[73]
Delitschiapyrone A (**133**)	458	$C_{24}H_{26}O_9$	*Delitschia* sp. FL1581	*Serenoa repens* (Fallen leaf of saw palmetto, Arecaceae)	pine-dominated forest, central Florida, USA	[28]
Variabilone (**134**)	228	$C_{15}H_{16}O_2$	*Paraconiothyrium variabile*	*Cephalotaxus harringtonia* (Taxaceae)	France	[76]
Juglanone C (**135**)	338	$C_{20}H_{18}O_5$	*Phialocephala fortinii*	*Pamelia* sp. (Lichen, Parmeliaceae)	Mount Qingliang, Zhejiang, China	[137]
Juglanone D (**136**)	340	$C_{20}H_{20}O_5$	*Phialocephala fortinii*	*Pamelia* sp. (Lichen, Parmeliaceae)	Mount Qingliang, Zhejiang, China	[137]
Juglanone E (**137**)	340	$C_{20}H_{20}O_5$	*Phialocephala fortinii*	*Pamelia* sp. (Lichen, Parmeliaceae)	Mount Qingliang, Zhejiang, China	[137]
(4*S*,10*R*,4`*S*)-Leptothalenone B (**138**)	472	$C_{25}H_{28}O_9$	*Leptosphaerulina chartarum* 3608	(Marine animal, Crinoidea)	Xuwen, Zhanjiang City, Guangdong, China	[91]
(4*R*,10*S*,4`*S*)-Leptothalenone B (**139**)	472	$C_{25}H_{28}O_9$	*Leptosphaerulina chartarum* 3608	Crinoid (Marine animal, Crinoidea)	Xuwen, Zhanjiang City,	[91]

					Guangdong, China	
Xylarinap A (**140**)	365	$C_{21}H_{19}NO_5$	*Xylaria nigripes*	Fresh fruiting bodies	Ailao Moutain, Yunnan, China	[29]
Xylarinap B (**141**)	336	$C_{20}H_{16}O_5$	*Xylaria nigripes*	Fresh fruiting bodies	Ailao Moutain, Yunnan, China	[29]
Xylarinap C (**142**)	336	$C_{20}H_{16}O_5$	*Xylaria nigripes*	Fresh fruiting bodies	Ailao Moutain, Yunnan, China	[29]
Xylarinap D (**143**)	350	$C_{21}H_{18}O_5$	*Xylaria nigripes*	Fresh fruiting bodies	Ailao Moutain, Yunnan, China	[29]
Xylarinap E (**144**)	350	$C_{21}H_{18}O_5$	*Xylaria nigripes*	Fresh fruiting bodies	Ailao Moutain, Yunnan, China	[29]
Cladosporol (= Cladosporol A) (**145**)	352	$C_{20}H_{16}O_6$	*Cladosporium cladosporioides*	*Lactuca sativa* L. (Seedlings, Asteraceae)	Japan	[163]
			Cladosporium cladosporioides	*Phytophthora capsici* (Peronosporaceae)		[164]
			Cladosporium. tenuissimum Cooke	-	-	[165]
			Cladosporium tenuissimum Cooke	*Pinus pinaster* (Pinaceae)	Tuscany, Italy	[166]
			Cladosporium sp. KcFL6'	*Kandelia candel* (Rhizophoraceae)	Daya Bay, Shenzhen city, Guangdong, China	[77]
			Cladosporium sp. TMPU1621	*Achyranthes aspera* var. *rubrofusca* (Leaves, Amaranthaceae)	Okinawan, Japan	[167]
			C. cladosporioides MRCJ-314	*Datura innoxia* (Roots, Solanaceae)	Jammu, India	[94]
Cladosporol B (**146**)	350	$C_{20}H_{14}O_6$	*C. tenuissimum* Cooke	*Pinus pinaster* (Pinaceae)	Tuscany, Italy	[166]
			Cladosporium sp. TMPU1621	*Achyranthes aspera* var. *rubrofusca* (Leaves, Amaranthaceae)	Okinawan, Japan	[167]
Cladosporol C (**147**)	338	$C_{20}H_{18}O_5$	*C. tenuissimum* Cooke	*Pinus pinaster* (Pinaceae)	Tuscany, Italy	[166]
			Cladosporium sp. KcFL6'	*Kandelia candel* (Rhizophoraceae)	Daya Bay, Shenzhen city,	[77]

					Guangdong, China	
			C. cladosporioides HDN14-342	Sediment	Indian Ocean, Qingdao, China	[95]
			C. cladosporioides EN-399	*Laurencia okamurai* (Rhodomelaceae)	Qingdao, China	[78]
			Cladosporium sp. TMPU1621	*Achyranthes aspera* var. *rubrofusca* (Leaves, Amaranthaceae)	Okinawan, Japan	[167]
			Cladosporium sp. JS1-2	*Ceriops tagal* (Rhizophoraceae)	Dongzhaigang, Hainan, China	[79]
			Cladosporium cladosporioides MCCC 3A00182	Sediment	Southwest Pacific Ocean	[115]
Cladosporol D (**148**)	354	$C_{20}H_{18}O_6$	*Cladosporium tenuissimum* Cooke	*Pinus pinaster* (Pinaceae)	Tuscany, Italy	[166]
			Cladosporium sp. KcFL6'	*Kandelia candel* (Rhizophoraceae)	Daya Bay, Shenzhen city, Guangdong, China	[77]
			Cladosporium sp. TMPU1621	*Achyranthes aspera* var. *rubrofusca* (Leaves, Amaranthaceae)	Okinawan, Japan	[167]
Cladosporol E (**149**)	370	$C_{20}H_{18}O_7$	*Cladosporium tenuissimum* Cooke	*Pinus pinaster* (Pinaceae)	Tuscany, Italy	[166]
			Cladosporium cladosporioides HDN14-342	Sediment	Indian Ocean, Qingdao, China	[95]
			Cladosporium sp. JS1-2	*Ceriops tagal* (Rhizophoraceae)	Dongzhaigang, Hainan, China	[79]
Cladosporol F (**150**)	352	$C_{21}H_{20}O_5$	*Cladosporium cladosporioides* HDN14-342	Sediment	Indian Ocean, Qingdao, China	[95]
			C. cladosporioides EN-399	*Laurencia okamurai* (Rhodomelaceae)	Qingdao, China	[78]
			Cladosporium sp. TMPU1621	*Achyranthes aspera* var. *rubrofusca* (Leaves, Amaranthaceae)	Okinawan, Japan	[167]
Cladosporol G (= 2-Chloro-cladosporol D) (**151**)	388	$C_{20}H_{17}ClO_6$	*Cladosporium* sp. TMPU1621	*Achyranthes aspera* var. *rubrofusca* (Leaves, Amaranthaceae)	Okinawan, Japan	[167]

	388	$C_{20}H_{17}ClO_6$	*Cladosporium cladosporioides* HDN14-342	Sediment	Indian Ocean, Qingdao, China	[95]
Cladosporol G (**152**)	352	$C_{21}H_{20}O_5$	*Cladosporium cladosporioides* EN-399	*Laurencia okamurai* (Rhodomelaceae)	Qingdao, China	[78]
Cladosporol H (**153**)	336	$C_{20}H_{16}O_5$	*Cladosporium cladosporioides* EN-399	*Laurencia okamurai* (Rhodomelaceae)	Qingdao, China	[78]
Cladosporol I (= Cladosperanol A) (**154**)	338	$C_{20}H_{18}O_5$	*Cladosporium cladosporioides* EN-399	*Laurencia okamurai* (Rhodomelaceae)	Qingdao, China	[78]
			Cladosporium sp. KFD33	Blood cockle (Cardiidae)	Haikou Bay, China	[80]
			Cladosporium perangustum FS62	Marine-derived fungus	China	[168]
Cladosporol J (**155**)	338	$C_{20}H_{18}O_5$	*Cladosporium cladosporioides* EN-399	*Laurencia okamurai* (Rhodomelaceae)	Qingdao, China	[78]
Altertoxin XII (**156**)	322	$C_{20}H_{18}O_4$	*Cladosporium* sp. KFD33	*Tegillarca granosa* (Blood cockle, Cardiidae)	Haikou Bay, China	[80]
Cladosporone A (**157**)	352	$C_{20}H_{16}O_6$	*Cladosporium* sp. KcFL6'	*Kandelia candel* (Rhizophoraceae)	Daya Bay, Shenzhen city, Guangdong, China	[77]
Clindanone A (**158**)	394	$C_{22}H_{18}O_7$	*Cladosporium cladosporioides* HDN14-342	Sediment	Indian Ocean, Qingdao, China	[95]
Clindanone B (**159**)	394	$C_{22}H_{18}O_7$	*Cladosporium cladosporioides* HDN14-342	Sediment	Indian Ocean, Qingdao, China	[95]

Table S2. Biological activities of fungal naphthalenones.

Compound name	Biological activity	Assay, organism, or cell line	Inhibitory concentration	Positive control	Ref.
3,4-Dihydro-3,4,8-trihydroxy-1(2*H*)-naphthalenone (**1**)	α-Glucosidase inhibitory	Spectrophotometer/α-Glucosidase enzyme	34.88 μM (IC_{50})	Acarbose 427.34 μM (IC_{50})	[82]
(3*R*,4*S*)-3,4,8-Trihydroxy-3,4-dihydronaphthalen-1(2*H*)-one (**3**)	Immunosuppressive	Immunoassay/IMPDH	4.1 μM (IC_{50})	Mycophenolic acid 0.4 μM (IC_{50})	[134]
	Immunosuppressive	Radioactively labeled phosphoprotein/PTPlB	13.9 μM (IC_{50})	Ursolic acid 2.8 μM (IC_{50})	[134]
	Antifungal	Broth dilution/*C. albicans*	63.2 μg/mL (IC_{50})	Amphotericin B 1.3 μg/mL (IC_{50})	[63]
	Antitumor	Immune complex phosphatase/SHP2	41.2 μM (IC_{50})	Ursolic acid 2.8 μM (IC_{50})	[134]
(2*S*,4*S*)-3,4-Dihydro-2,4,8-trihydroxy-l(2*H*)-naphthalenone (**4**)	α-Glucosidase inhibitory	Colorimetric/α-Glucosidase enzyme	102.34 μM (IC_{50})	Acarbose 427.34 μM (IC_{50})	[82]
4,6-Dihydroxy - 3,4-dihydronaphthalen-1(2*H*)-one (**8**)	Nematocidal	Nematotoxin bioassay/ *Bursaphelenchus xylophilus*	1169.8 mg/L (LC_{50}, 12 h)		[52]
			461.3 mg/L (LC_{50}, 24 h)		[52]
			229.6 mg/L (LC_{50}, 36 h)		[52]
4,8-dihydroxy-3,4-dihydronaphthalen-1(2*H*)-one (**9**)	Nematocidal	Nematotoxin bioassay/ *Bursaphelenchus xylophilus*	540.2 mg/L (LC_{50}, 12 h)		[52]
			436.6 mg/L (LC_{50}, 24 h)		[52]
			209.7 mg/L (LC_{50}, 36 h)		[52]
4(*S*)-(+)-Isosclerone acetate (**10**)	Antibacterial	Agar diffusion assay/*B. megaterium*	15.0 mm (IZD)	Penicillin 28.0 mm (IZD)	[60]
	Antimycobacterial	MABA/*Mycobacterium tuberculosis* H37 Ra	50 μg/mL (MIC)	Isoniazid 0.05 μg/mL (MIC)<br>Kanamycin 2.5 μg/mL (MIC)	[61]
	Cytotoxicity	SRB/BCA	11.27 μg/mL (IC_{50})	Ellipticine 0.11 μg/mL (IC_{50})<br>Doxorubicin 0.21 μg/mL (IC_{50})	[61]
		MTT/NCI-H187	7.29 μg/mL (IC_{50})	Ellipticine 0.32 μg/mL (IC_{50})<br>Doxorubicin 0.02 μg/mL (IC_{50})	[61]
		SRB/Vero	41.84 μg/mL (IC_{50})	Ellipticine 0.7 μg/mL (IC_{50})	[61]

(3*S*,4*R*)-4,8-dihydroxy-3-methoxy-3,4-dihydro-1(2*H*)-naphthalenone (**13**)	Antimycobacterial	MABA/*Mycobacterium tuberculosis* H37 Ra	12.5 μg/mL (MIC)	Isoniazid 0.05 μg/mL (MIC)<br>Kanamycin 2.5 μg/mL (MIC)	[61]
	Cytotoxicity	SRB/BCA	19.16 μg/mL (IC_{50})	Ellipticine 0.11 μg/mL (IC_{50})<br>Doxorubicin 0.21 μg/mL (IC_{50})	[61]
		MTT/NCI-H187	2.86 μg/mL (IC_{50})	Ellipticine 0.32 μg/mL (IC_{50})<br>Doxorubicin 0.02 μg/mL (IC_{50})	[61]
		SRB/Vero	26.59 μg/mL (IC_{50})	Ellipticine 0.7 μg/mL (IC_{50})	[61]
3,8-dihydroxy-3,4-dihydronaphthalen-1(2*H*)-one (**14**)	Antimycobacterial	MABA/*Mycobacterium tuberculosis* H37 Ra	50 μg/mL (MIC)	Isoniazid 0.05 μg/mL (MIC)<br>Kanamycin 2.5 μg/mL (MIC)	[61]
		MTT/NCI-H187	15.33 μg/mL (IC_{50})	Ellipticine 0.32 μg/mL (IC_{50})<br>Doxorubicin 0.02 μg/mL (IC_{50})	[61]
3,4,6,8-Tetrahydroxy-3,4-dihydronaphthalen-1(2*H*)-one (*cis*-4-hydroxyscytalone) (**19**)	Nematocidal	Nematotoxin bioassay/ *Bursaphelenchus xylophilus*	844.9 mg/L (LC_{50}, 12 h)		[52]
			468.4 mg/L (LC_{50}, 24 h)		[52]
			206.1 mg/L (LC_{50}, 36 h)		[52]
4,6,8-Trihydroxy-3,4-dihydronaphthalen-1(2*H*)-one) (**22**)	Nematocidal	Nematotoxin bioassay *Bursaphelenchus xylophilus*	1011.6 mg/L (LC_{50}, 12 h)		[52]
			522.5 mg/L (LC_{50}, 24 h)		[52]
			220.3 mg/L (LC_{50}, 36 h)		[52]
	Antimycobacterial	MABA/*Mycobacterium tuberculosis* H37 Ra	12.5 μg/mL (MIC)	Isoniazid 0.05 μg/mL (MIC)<br>Kanamycin 2.5 μg/mL (MIC)	[61]
	Cytotoxicity	SRB/BCA	2.96 μg/mL (IC_{50})	Ellipticine 0.11 μg/mL (IC_{50})<br>Doxorubicin 0.21 μg/mL (IC_{50})	[61]
		MTT/NCI-H187	5.55 μg/mL (IC_{50})	Ellipticine 0.32 μg/mL (IC_{50})<br>Doxorubicin 0.02 μg/mL (IC_{50})	[61]
		SRB/Vero	24.35 μg/mL (IC_{50})	Ellipticine 0.7 μg/mL (IC_{50})	[61]
(*R*)-4,6,8-Trihydroxy-3,4-dihydro-1(2*H*)-naphthalenone (**25**)	Antibacterial	Broth dilution/*B. subtilis*	104.2 μg/mL (IC_{50})	Streptomycin sulfate 5.2 μg/mL (IC_{50})	[63]
	Antifungal	Broth dilution/*C. albicans*	78.2 μg/mL (IC_{50})	Amphotericin B 1.3 μg/mL (IC_{50})	[63]
(3*S*,4*S*)-3,4,8-trihydroxy-6-methoxy-3,4-dihydro-1(2*H*)-naphthalenone (**31**)	Antimycobacterial	MABA/*Mycobacterium tuberculosis* H37 Ra	25.0 μg/mL (MIC)	Isoniazid 0.05 μg/mL (MIC)<br>Kanamycin 2.5 μg/mL (MIC)	[61]
	Cytotoxicity	SRB/BCA	7.24 μg/mL (IC_{50})	Ellipticine 0.11 μg/mL (IC_{50})<br>Doxorubicin 0.21 μg/mL (IC_{50})	[61]

		MTT/NCI-H187	12.08 μg/mL (IC_{50})	Ellipticine 0.32 μg/mL (IC_{50})<br>Doxorubicin 0.02 μg/mL (IC_{50})	[61]
		SRB/Vero	26.35 μg/mL (IC_{50})	Ellipticine 0.7 μg/mL (IC_{50})	[61]
(3*R*,4*R*)-3-methoxyl-botryosphaerone D (**54**)	Indoleamine 2, 3-dioxygenase (IDO) inhibitory	High-Throughput Fluorescence assay/L-tryptophan	6.36 μM (IC_{50})	Epacadostat 0.5 μM (IC_{50})	[107]
(3*R**,4*S**)-7-Ethyl-3,4,6,8-tetrahydroxy-3,4-dihydronaphthalen-1(2*H*)-one (**61**)	Antifungal	Agar diffusion/*Alternaria* sp. YMF 1.01991	7.0 mm (IZD)	Ciclopirox 26.0 mm (IZD)	[67]
	Antifungal	Agar diffusion/*Sclerotium* sp. YMF 1.01993	8.0 mm (IZD)	Ciclopirox 19.0 mm (IZD)	[67]
	Antifungal	Agar diffusion/*Fusarium* sp. YMF 1.01996	7.0 mm (IZD)	Ciclopirox 20.0 mm (IZD)	[67]
	Antibacterial	Agar diffusion/*B. cereus* YMF 3.19	12.0 mm (IZD)	Ampicillin sodium 35.0 mm (IZD)	[67]
	Antibacterial	Agar diffusion/*B. laterosporus* YMF 3.08	10.0 mm (IZD)	Ampicillin sodium 30.0 mm (IZD)	[67]
	Antibacterial	Agar diffusion/*S. aureus* YMF 3.17	20.0 mm (IZD)	Ampicillin sodium 19.0 mm (IZD)	[67]
Nodulone (**63**)	Cytotoxicity	MTT/Mouse splenic lymphocytes	11.2 μg/mL (IC_{50})	Cyclosporine A 0.06 μg/mL (IC_{50})	[27]
(-)-*cis*-(7*R**,8*S**)-4,7,8-Trihydroxy-3,6,7,8-tetrahydronaphtho[2,3-*c*]furan-5(1*H*)-one (**64**)	Antimalarial	Microculture radioisotope-[^{3}H]-hypoxanthine/*P. falciparum* (K1, multidrug-resistant strain)	3.125 μg/mL (IC_{50})	Dihydroartemisinin 1.91 nM (IC_{50})<br>Mefloquine 24.7 nM (IC_{50})	[66]
	Antimycobacterial	GFPMA/*Mycobacterium tuberculosis* H37 Ra	11.3 μg/mL (IC_{50})	Rifampicin, ofloxacin, streptomycin, isoniazid, and ethambutol	[66]
3,6,8-Trihydroxy-3-[3,5-dimethyl-2-oxo-3(*E*)-heptenyl]-2,3-dihydronaphthalen-1(4*H*)-one (**68**)	Antifungal	Broth microdilution/*C. albicans*	40 μg/mL (MIC)	Ketonazole 1.0 μg/mL (MIC)	[69]
	Antifungal	Broth microdilution/ *Tricophyton rubrum*	20 μg/mL (MIC)	Ketonazole 10.0 μg/mL (MIC)	[69]
	Antifungal	Broth microdilution/*A. niger*	80 μg/mL (MIC)	Ketonazole 30.0 μg/mL (MIC)	[69]
Balticol A (**74**)	Antiviral	Dye-uptake assay/*Herpes simplex* virus type 1 (HSV-1, strain KOS)	1.0 μg/mL (IC_{50})	Aciclovir 0.1 μg/mL (IC_{50})	[70]

Balticol B (**75**)	Antiviral	Dye-uptake assay/Human influenza virus A/WSN/33 (H1N1)	10.0 μg/mL (IC_{50})	Amantadine SO_4 15.0 μg/mL (IC_{50})	[70]
		Dye-uptake assay/*Herpes simplex* virus type 1 (HSV-1, strain KOS)	1.0 μg/mL (IC_{50})	Aciclovir 0.1 μg/mL (IC_{50})	[70]
Balticol C (**78**)	Antiviral	Dye-uptake assay/Human influenza virus A/WSN/33 (H1N1)	1.0 μg/mL (IC_{50})	Amantadine SO_4 15.0 μg/mL (IC_{50})	[70]
		Dye-uptake assay/*Herpes simplex* virus type 1 (HSV-1, strain KOS)	1.0 μg/mL (IC_{50})	Aciclovir 0.1 μg/mL (IC_{50})	[70]
Balticol D (**80**)	Antiviral	Dye-uptake assay/Human influenza virus A/WSN/33 (H1N1)	0.1 μg/mL (IC_{50})	Amantadine SO_4 15.0 μg/mL (IC_{50})	[70]
		Dye-uptake assay/*Herpes simplex* virus type 1 (HSV-1, strain KOS)	0.1 μg/mL (IC_{50})	Aciclovir 0.1 μg/mL (IC_{50})	[70]
Balticol E (**81**)	Antiviral	Dye-uptake assay/*Herpes simplex* virus type 1 (HSV-1, strain KOS)	0.01 μg/mL (IC_{50})	Aciclovir 0.1 μg/mL (IC_{50})	[70]
Balticol F (**82**)	Antiviral	Dye-uptake assay/Human influenza virus A/WSN/33 (H1N1)	1.0 μg/mL (IC_{50})	Amantadine SO_4 15.0 μg/mL (IC_{50})	[70]
		Dye-uptake assay/*Herpes simplex* virus type 1 (HSV-1, strain KOS)	0.1 μg/mL (IC_{50})	Aciclovir 0.1 μg/mL (IC_{50})	[70]
9α-Hydroxyhalorosellinia A (**85**)	Antimycobacterial	MABA/*M. tuberculosis* H37 Ra	12.5 μg/mL (MIC)	Rifampicin 0.047 μg/mL (MIC)<br>Kanamycin sulfate 1.25 μg/mL (MIC)<br>Isoniazid 0.25 μg/mL (MIC)	[71]
	Antimalarial	Microculture radioisotope-[^{3}H]-hypoxanthine/*P. falciparum* (K1, multidrug-resistant strain)	7.94 μg/mL (IC_{50})	Dihydroartimisinin 0.0012 mg/mL (IC_{50})	[71]
9*S*-hydroxydihydrodesoxybostrycin (**89**)	Antimycobacterial	MABA/*Mycobacterium tuberculosis* H37 Ra	25.0 μg/mL (MIC)	Rifampicin 0.047 μg/mL (MIC)<br>Kanamycin sulfate 1.25 μg/mL (MIC)	[71]

				Isoniazid 0.25 μg/mL (MIC)	
Perenniporide A (**90**)	Antifungal	Microplate assay/*F. moniliforme*	20 μg/mL (MIC)	2-Benzimidazolecarbamate 0.63 μg/mL (MIC)	[73]
	Antifungal	Microplate assay/ *V. alboatrum*	20 μg/mL (MIC)	2-Benzimidazolecarbamate 0.63 μg/mL (MIC)	[73]
	Antifungal	Microplate assay/*Gibberella zeae*	20 μg/mL (MIC)	2-Benzimidazolecarbamate 2.5 μg/mL (MIC)	[73]
	Antifungal	Microplate assay/*F. oxysporum*	10 μg/mL (MIC)	2-Benzimidazolecarbamate 0.63 μg/mL (MIC)	[73]
	Antifungal	Microplate assay/*A. longipes*	10 μg/mL (MIC)	-	[73]
Dihydronaphthalenone (**98**)	Cytotoxicity	Resazurin microplate/KB	31.69 μg/mL (IC_{50})	Doxorubicin 0.28 μg/mL (IC_{50}) Ellipticine 0.32 μg/mL (IC_{50})	[75]
	Cytotoxicity	Resazurin microplate/NCI-H187	23.10 μg/mL (IC_{50})	Doxorubicin 0.07 μg/mL (IC_{50}) Ellipticine 1.21 μg/mL (IC_{50})	[75]
Diastereomer-dihydronaphthalenone (**99**)	Antimycobacterial	GFPMA/*M. tuberculosis* H37 Ra	25.0 μg/mL (MIC)	Isoniazid 0.03 μg/mL (MIC)	[75]
5-Hydroxydihydrofusarubin (**105**)	Cytotoxicity	Resazurin microplate/L1210	9.4 μg/mL (IC_{50})	-	[160]
	Antibacterial	Agar plate dilution/*S. aureus*	100 μg/mL (MIC)	-	
	Antibacterial	Agar plate dilution/*B. subtitis*	100 μg/mL (MIC)	-	[160]
	Antibacterial	Agar plate dilution/*S. pneumoniae*	50 μg/mL (MIC)	-	[160]
	Antifungal	Agar plate dilution/*C. ablicans*	100 μg/mL (MIC)	-	[160]
	Antifungal	Agar platedilution/ *Saccharomyces cereviciae*	50 μg/mL (MIC)	-	[160]
5-Hydroxydihydrofusarubin A (**106**)	Cytotoxicity	Resazurin microplate/KB	21.25 μg/mL (IC_{50})	Doxorubicin 0.28 μg/mL (IC_{50}) Ellipticine 0.32 μg/mL (IC_{50})	[75]
		Resazurin microplate/MCF-7	10.99 μg/mL (IC_{50})	Doxorubicin 0.90 μg/mL (IC_{50})	[75]
		Resazurin microplate/NCI-H187	12.14 μg/mL (IC_{50})	Doxorubicin 0.07 μg/mL (IC_{50}) Ellipticine 1.21 μg/mL (IC_{50})	[75]
5-Hydroxy-3-methoxydihydrofusarubin A (**109**)	Antimycobacterial	GFPMA/*M. tuberculosis* H37 Ra	50.0 μg/mL (MIC)	Isoniazid 0.03 μg/mL (MIC)	[75]
	Cytotoxicity	Resazurin microplate/KB	25.48 μg/mL (IC_{50})	Doxorubicin 0.28 μg/mL (IC_{50}) Ellipticine 0.32 μg/mL (IC_{50})	[75]
	Cytotoxicity	Resazurin microplate/MCF-7	14.50 μg/mL (IC_{50})	Doxorubicin 0.90 μg/mL (IC_{50})	[75]

	Cytotoxicity	Resazurin microplate/NCI-H187	26.03 μg/mL (IC_{50})	Doxorubicin 0.07 μg/mL (IC_{50}) Ellipticine 1.21 μg/mL (IC_{50})	[75]
5-Hydroxydihydrofusarubin B (**111**)	Cytotoxicity	Resazurin microplate/KB	31.50 μg/mL (IC_{50})	Doxorubicin 0.28 μg/mL (IC_{50}) Ellipticine 0.32 μg/mL (IC_{50})	[75]
	Cytotoxicity	Resazurin microplate/NCI-H187	11.89 μg/mL (IC_{50})	Doxorubicin 0.07 μg/mL (IC_{50}) Ellipticine 1.21 μg/mL (IC_{50})	[75]
5-Methoxydihydrofusarubin B (**112**)	Cytotoxicity	Resazurin microplate/KB	23.59 μg/mL (IC_{50})	Doxorubicin 0.28 μg/mL (IC_{50}) Ellipticine 0.32 μg/mL (IC_{50})	[75]
	Cytotoxicity	Resazurin microplate/MCF-7	23.43 μg/mL (IC_{50})	Doxorubicin 0.90 μg/mL (IC_{50})	[75]
	Cytotoxicity	Resazurin microplate/NCI-H187	13.47 μg/mL (IC_{50})	Doxorubicin 0.07 μg/mL (IC_{50}) Ellipticine 1.21 μg/mL (IC_{50})	[75]
	Cytotoxicity	Resazurin microplate/Vero	28.26 μg/mL (IC_{50})	Ellipticine 1.72 μg/mL (IC_{50})	[75]
5-Hydroxydihydrofusarubin C (**114**)	Cytotoxicity	Resazurin microplate/KB	9.34 μg/mL (IC_{50})	Doxorubicin 0.28 μg/mL (IC_{50}) Ellipticine 0.32 μg/mL (IC_{50})	[75]
	Cytotoxicity	Resazurin microplate/MCF-7	16.58 μg/mL (IC_{50})	Doxorubicin 0.90 μg/mL (IC_{50})	[75]
	Cytotoxicity	Resazurin microplate/NCI-H187	9.99 μg/mL (IC_{50})	Doxorubicin 0.07 μg/mL (IC_{50}) Ellipticine 1.21 μg/mL (IC_{50})	[75]
	Cytotoxicity	Resazurin microplate/Vero	26.76 μg/mL (IC_{50})	Ellipticine 1.72 μg/mL (IC_{50})	[75]
Aspvanicin B (**128**)	Cytotoxicity	MTT/L5178Y	22.8 μM (IC_{50})	Kahalalide F 4.3 μM (IC_{50})	[89]
Phomonaphthalenone A (**130**)	Cytotoxicity	MTT/A549	92.5 μg/mL (IC_{50})	5-Fluorouracil 75.0 μg/mL (IC_{50})	[90]
	Cytotoxicity	MTT/MDA-MB-231	64.2 μg/mL (IC_{50})	5-Fluorouracil 47.0 μg/mL (IC_{50})	[90]
	Cytotoxicity	MTT/PANC-1	52.7 μg/mL (IC_{50})	5-Fluorouracil 65.0 μg/mL (IC_{50})	[90]
	Anti-HIV	Luciferase assay/293T cells	11.6 μg/mL (IC_{50})	Efavirenz 4.7×10^{-4} μg/mL (IC_{50})	[90]
Variabilone (**134**)	Antibacterial	Microdilution resazurin/*B. subtilis*	2.13 μg/mL (IC_{50})	Kanamycin 0.36 μg/mL (IC_{50})	[76]
(4R,10S,4`S)-Leptothalenone B (**139**)	Anti-inflammatory	Griess assay/LPS-activated NO production/ RAW264.7 cells	44.5 μM (IC_{50})	Indomethacin 37.5 μM (IC_{50})	[91]
Cladosporol C (**147**)	Cytotoxicity	Trypan blue-cell viability assay/K562	>30.0 μM (IC_{50})	Trichostatin A 0.24 μM (IC_{50})	[77]
		Trypan blue-cell viability assay/A549	33.9 μM (IC_{50})	Trichostatin A 0.05 μM (IC_{50})	[77]
		Trypan blue-cell viability assay/Huh-7	>30.0 μM (IC_{50})	Trichostatin A 0.08 μM (IC_{50})	[77]
		Trypan blue-cell viability assay/H1975	45.6 μM (IC_{50})	Trichostatin A 0.09 μM (IC_{50})	[77]

		Trypan blue-cell viability assay/MCF-7	>30.0 μM (IC_{50})	Trichostatin A 0.78 μM (IC_{50})	[77]
		Trypan blue-cell viability assay/U937	>30.0 μM (IC_{50})	Trichostatin A 0.06 μM (IC_{50})	[77]
		Trypan blue cell viability assay/BGC823	>30.0 μM (IC_{50})	Trichostatin A 0.09 μM (IC_{50})	[77]
		Trypan blue cell viability assay/HL-60	72.5 μM (IC_{50})	Trichostatin A 0.09 μM (IC_{50})	[77]
		Trypan blue cell viability assay/A549	>30.0 μM (IC_{50})	Trichostatin A 0.11 μM (IC_{50})	[77]
		Trypan blue cell viability assay/MOLT-4	14.4 μM (IC_{50})	Trichostatin A 0.03 μM (IC_{50})	[77]
		MTT/A549	14.0 μM (IC_{50})	Cisplatin 1.3 μM (IC_{50})	[78]
		MTT/HeLa	4.0 μM (IC_{50})	Paclitaxel 4.9 μM (IC_{50})	[78]
	Antimicrobial	Microplate assay/*E. coli*	8.0 μg/mL (MIC)	Chloramphenicol 0.025 μg/mL (MIC)	[78]
		Microplate assay/*M. luteus*	32.0 μg/mL (MIC)	Chloramphenicol 0.5 μg/mL (MIC)	[78]
		Microplate assay/*V. harveyi*	16.0 μg/mL (MIC)	Chloramphenicol 2.0 μg/mL (MIC)	[78]
		Microplate assay/*S. aureus*	6.25 μg/mL (MIC)	Ciprofloxacin 0.39 μg/mL (MIC)	[79]
		Microplate assay/*M. luteus*	12.5 μg/mL (MIC)	Ciprofloxacin 0.39 μg/mL (MIC)	[79]
Cladosporol D (**148**)	Anti-inflammatory	Spectrophotometry/Anti-COX-2/$PGF_{2\alpha}$ inhibitor	60.2 μM (IC_{50})	Indomethacin 18.3 μM (IC_{50})<br>NS-398 1.0 μM (IC_{50})	[77]
Cladosporol E (**149**)	Insecticidal activity	Measuring the corrected mortality (CM)	150.0 μg/mL (IC_{50})	Azadirachtin 25.0 μg/mL (IC_{50})	[79]
	Antimicrobial	Microplate assay/*S. aureus*	1.56 μg/mL (MIC)	Ciprofloxacin 0.39 μg/mL (MIC)	[79]
		Microplate assay/*M. luteus*	12.5 μg/mL (MIC)	Ciprofloxacin 0.39 μg/mL (MIC)	[79]
Cladosporol F (**150**)	Cytotoxicity	MTT/K562	23.0 μM (IC_{50})	Doxorubicin 0.6 μM (IC_{50})	[95]
		SRB/HeLa	13.8 μM (IC_{50})	Doxorubicin 0.5 μM (IC_{50})	[95]
		SRB/HCT-116	23.0 μM (IC_{50})	Doxorubicin 0.2 μM (IC_{50})	[95]
		MTT/A549	15.0 μM (IC_{50})	Cisplatin 1.3 μM (IC_{50})	[78]
		MTT/HeLa	10.0 μM (IC_{50})	Paclitaxel 4.9 μM (IC_{50})	[78]
	Antimicrobial	Microplate assay/*E. coli*	32.0 μg/mL (MIC)	Chloramphenicol 0.025 μg/mL (MIC)	[78]
		Microplate assay/*M. luteus*	64.0 μg/mL (MIC)	Chloramphenicol 0.5 μg/mL (MIC)	[78]

		Microplate assay/*V. harveyi*	32.0 µg/mL (MIC)	Chloramphenicol 2.0 µg/mL (MIC)	[78]
Cladosporol G (**151**)	Cytotoxicity	MTT/K562	8.8 µM (IC_{50})	Doxorubicin 0.6 µM (IC_{50})	[95]
		SRB/HeLa	3.9 µM (IC_{50})	Doxorubicin 0.5 µM (IC_{50})	[95]
		SRB/HCT-116	19.4 µM (IC_{50})	Doxorubicin 0.2 µM (IC_{50})	[95]
Cladosporol G (**152**)	Cytotoxicity	MTT/A549	13.0 µM (IC_{50})	Cisplatin 1.3 µM (IC_{50})	[78]
		MTT/H446	11.0 µM (IC_{50})	Adriamycin 4.0 µM (IC_{50})	[78]
		MTT/Huh7	10.0 µM (IC_{50})	Fluorouracil 6.2 µM (IC_{50})	[78]
		MTT/L02	11.0 µM (IC50)	Cisplatin 13.0 µM (IC_{50})	[78]
		MTT/LM3	14.0 µM (IC50)	Cisplatin 9.1 µM (IC_{50})	[78]
		MTT/SW1990	15.0 µM (IC_{50})	Gemcitabine 2.2 µM (IC_{50})	[78]
	Antimicrobial	Microplate assay/*E. coli*	64.0 µg/mL (MIC)	Chloramphenicol 0.025 µg/mL (MIC)	[78]
		Microplate assay/*M. luteus*	128.0 µg/mL (MIC)	Chloramphenicol 0.5 µg/mL (MIC)	[78]
		Microplate assay/*V. harveyi*	64.0 µg/mL (MIC)	Chloramphenicol 2.0 µg/mL (MIC)	[78]
Cladosporol H (**153**)	Cytotoxicity	MTT/A549	5.0 µM (IC_{50})	Cisplatin 1.3 µM (IC_{50})	[78]
		MTT/H446	10.0 µM (IC_{50})	Adriamycin 4.0 µM (IC_{50})	[78]
		MTT/Huh7	1.0 µM (IC_{50})	Fluorouracil 6.2 µM (IC_{50})	[78]
		MTT/LM3	4.1 µM (IC_{50})	Cisplatin 9.1 µM (IC_{50})	[78]
		MTT/MCF-7	10.0 µM (IC_{50})	Paclitaxel 1.8 µM (IC_{50})	[78]
		MTT/SW1990	15.0 µM (IC_{50})	Gemcitabine 2.2 µM (IC_{50})	[78]
	Antimicrobial	Microplate assay/*E. coli*	32.0 µg/mL (MIC)	Chloramphenicol 0.025 µg/mL (MIC)	[78]
		Microplate assay/*M. luteus*	64.0 µg/mL (MIC)	Chloramphenicol 0.5 µg/mL (MIC)	[78]
		Microplate assay/*V. harveyi*	4.0 µg/mL (MIC)	Chloramphenicol 2.0 µg/mL (MIC)	[78]
Cladosporol I (**154**)	Cytotoxicity	MTT/HeLa	10.8 µM (IC_{50})	Paclitaxel 4.9 µM (IC_{50})	[78]
	Antimicrobial	Microplate assay/*E. coli*	64.0 µg/mL (MIC)	Chloramphenicol 0.025 µg/mL (MIC)	[78]
		Microplate assay/*M. luteus*	64.0 µg/mL (MIC)	Chloramphenicol 0.5 µg/mL (MIC)	[78]
		Microplate assay/*V. harveyi*	16.0 µg/mL (MIC)	Chloramphenicol 2.0 µg/mL (MIC)	[78]

Cladosporol J (**155**)	Cytotoxicity	MTT/A549	15.0 μM (IC_{50})	Cisplatin 1.3 μM (IC_{50})	[78]
		MTT/H446	11.0 μM (IC_{50})	Adriamycin 4.0 μM (IC_{50})	[78]
		MTT/HeLa	15.0 μM (IC_{50})	Paclitaxel 4.9 μM (IC_{50})	[78]
		MTT/Huh7	20.0 μM (IC_{50})	Fluorouracil 6.2 μM (IC_{50})	[78]
		MTT/MCF-7	12.0 μM (IC_{50})	Paclitaxel 1.8 μM (IC_{50})	[78]
	Antimicrobial	Microplate assay/*E. coli*	16.0 μg/mL (MIC)	Chloramphenicol 0.025 μg/mL (MIC)	[78]
		Microplate assay/*M. luteus*	64.0 μg/mL (MIC)	Chloramphenicol 0.5 μg/mL (MIC)	[78]
		Microplate assay/*V. harveyi*	32.0 μg/mL (MIC)	Chloramphenicol 2.0 μg/mL (MIC)	[78]
Cladosporone A (**157**)	Cytotoxicity	Trypan blue cell viability assay/K562	14.3 μM (IC_{50})	Trichostatin A 0.24 μM (IC_{50})	[77]
		Trypan blue cell viability assay/A549	15.7 μM (IC_{50})	Trichostatin A 0.05 μM (IC_{50})	[77]
		Trypan blue cell viability assay/Huh-7	29.9 μM (IC_{50})	Trichostatin A 0.08 μM (IC_{50})	[77]
		Trypan blue cell viability assay/H1975	40.6 μM (IC_{50})	Trichostatin A 0.09 μM (IC_{50})	[77]
		Trypan blue cell viability assay/MCF-7	21.3 μM (IC_{50})	Trichostatin A 0.78 μM (IC_{50})	[77]
		Trypan blue cell viability assay/U937	10.5 μM (IC_{50})	Trichostatin A 0.06 μM (IC_{50})	[77]
		Trypan blue cell viability assay/BGC823	17.0 μM (IC_{50})	Trichostatin A 0.09 μM (IC_{50})	[77]
		Trypan blue cell viability assay/HL-60	10.1 μM (IC_{50})	Trichostatin A 0.09 μM (IC_{50})	[77]
		Trypan blue cell viability assay/A549	53.7 μM (IC_{50})	Trichostatin A 0.11 μM (IC_{50})	[77]
		Trypan blue cell viability assay/MOLT-4	14.6 μM (IC_{50})	Trichostatin A 0.03 μM (IC_{50})	[77]
	Anti-inflammatory	Spectrophotometry/Anti-COX-2/$PGF_{2\alpha}$ inhibitor	49.1 μM (IC_{50})	Indomethacin 18.3 μM (IC_{50}) NS-398 1.0 μM (IC_{50})	[77]

118. ReferencesHemingway, R.W.; McGraw, G.W.; Barras, S.J. Polyphenols in *Ceratocystis minor* infected *Pinus* taeda: Fungal metabolites, phloem and xylem phenols. *J. Agric. Food Chem*, 1977, *25*, 717–723.
119. - Stipanovic, R.D.; Bell, A.A. Pentaketide metabolites of *Verticillium dahliae*. II. Accumulation of naphthol derivatives by the aberrant-melanin mutant BRM-2. *Mycologia* 1977, *69*, 164–172.

120. -Ayer, W.A.; Browne, L.M.; Lin G. Metabolites of Leptographium wageneri, the causative agent of black stain root disease of conifers. *J. Nat. Prod.* 1989, 52, 1, 119–129.
121. Borgschulte, K.; Rebuffat, S.; Trowitzsch-Kienast, W.; Schomburg, D.; Pinon, J.; Bodo, B. Isolation and structure elucidation of hymatoxins B - E and other phytotoxins from *Hypoxylon mammatum* fungal pathogen of leuce poplars. *Tetrahedron* **1991**, *47*, 8351–8360.
122. Tabuchi, H.; Tajimi, A.; Ichihara, A. Phytotoxic metabolites isolated from *Scolecotrichum graminis* Fuckel. *Biosci. Biotech. Biochem*. 1994, *58*, 1956–1959.
123. Trisuwan, K.; Rukachaisirikul, V.; Sukpondma, Y.; Preedanon, S.; Phongpaichit, S.; Rungjindamai, N.; Sakayaroj. J. Epoxydons and a pyrone from the marine-derived fungus *Nigrospora* sp. PSU-F5. *J. Nat. Prod.* 2008, *71*, 1323–1326.
124. Arunpanichlert, J.; Rukachaisirikul, V.; Phongpaichit, S.; Supaphon, O.; Sakayaroj, J. Xylariphilone: a new azaphilone derivative from the seagrass-derived fungus *Xylariales* sp. PSU-ES163. *Nat. Prod. Res*. 2016, 30, 46-51.
125. Fan, C.; Zhou, G.; Wang, W.; Zhang, G.; Zhu, T.; Che, Q.; Li, D. Tetralone derivatives from a deep-sea derived fungus *Cladosporium* sp. HDN17-58. *Nat. Prod. Commun*. 2021, *16*, 1–4.128.
126. Sviridov, S.I. Secondary metabolites of Pyricularia oryzae. Chem. Nat. Compd. 1991, 27, 410–413.
127. Zhang, Y.; Feng, Y.; Kramer, M.; Essmann, F.; Grond, S. A New Acetylenic compound and other bioactive metabolites from a shark gill-derived *Penicillium* strain. *Rec. Nat. Prod.* 2017, *11*, 31–36.
128. Quang, T.H.; Huong, P.T.M.; Ngan, N.T.T.; Hanh, T.T.H.; Cuong, N.X.; Nam, N.H.; Minh, C.V. Secondary metabolites from a marine sponge-associated fungus *Xenomyrothecium* sp. IMBC-FP2.11. *Vietnam J. Chem.* 2020, *58*, 752–758.
129. Iwasaki, S.; Muro, H.; Sasaki, K.; Nozoe, S.; Okuda, S.; Sato, Z. Isolations of phytotoxic substances produced by *pyricularia oryzae cavara*, *Tetrahedron Lett.* **1973**, *14*, 3537–3542.
130 Krohn, K.; Biele, C.; Drogies, K.H.; Steingrover, K.; Aust, H.J.; Draeger, S.; Schulz, B. Fusidilactones, a new group of polycyclic lactones from an endophyte, *Fusidium* sp. *Eur. J. Org. Chem.* 2002, *14*, 2331–2336.
131. Yamaguchi, Y.; Masuma, R.; Kim, Y-P.; Uchida, R.; Tomoda, H.; Omura, S. Taxonomy and secondary metabolites of *Pseudobotrytis* sp. FKA-25. *Mycoscience* 2004, *45*, 9–16.
132. Mancilla, G.; Jiménez-Teja, D.; Femenía-Rios, M.; Macías-Sánchez, A.J.; Collado, I.G.; Hernández-Galán, R. Novel macrolide from wild strains of the phytopathogen Fungus *Colletotrichum acutatum*. *Nat. Prod. Commun*. 2009, *4*, 395–398.
133. Ouchbani, T.; Zouihri, H.; Essassi, E.; Proksch, P.; Ng, S.W. (3*R*,4*S*)-3,4,8-Trihydroxy-1,2,3,4-tetrahydronaphthalen-1-one monohydrate from Embellisia eureka. *Acta Cryst*. 2012, *E68*, o1874.
134. Nong, X.H.; Zheng, Z.H.; Zhang, X.Y.; Lu, X.H.; Qi, S.H. Polyketides from a marine-derived fungus *Xylariaceae* sp. *Mar***. Drugs** 2013**, 11, 1718–1727.**137.
135. Venkatasubbaiah, P.; Chilton, W.S.J. Toxins produced by the Dogwood Anthracnose fungus *Discula* sp. *J. Nat. Prod.* 1991, *54*, 1293–1297.
136. Höller, U.; Konig, G.M.; Wright, A.D. Three new metabolites from marine-derived fungi of the genera *coniothyrium* and *microsphaeropsis*. *J. Nat. Prod*. 1999, *62*, 114–118.
137. Xie, F.; Chang, W.; Zhang, M.; Li, Y.; Li, W.; Shi, H.; Zheng, S.; Lou, H. Quinone derivatives isolated from the endolichenic fungus *Phialocephala fortinii* are Mdr1 modulators that combat azole resistance in *Candida albicans*. *Sci. Rep.* 2016, *6*, 33687.
138. De Souza, E.M.C.; Da Silva, E.L.; Marinho, A.M.R.; Marinho, P.S.B. (4*S*)-4,8-dihydroxy-1-tetralone and other chemical constituents from *Pestalotiopsis* sp. EJC07, endophytic from *Bauhinia guianensis*. *An. Acad. Bras. Ciênc.* 2016, *88*, 29–33
139. Hsiao, Y.; Cheng, M.J.; Chang, H.S.; Wu, M.D.; Hsieh, S.Y.; Liu, T.W.; Lin, C.H. Yuan, G.F.; Chen, I.S. Six new metabolites produced by *Colletotrichum aotearoa* 09F0161, an endophytic fungus isolated from *Bredia oldhamii*. *Nat. Prod. Res.* 2016, *30*, 251–258.
140. Kokubun, T.; Veitch, N.C.; Bridge, P.D.; Simmonds, M.S.J. Dihydroisocoumarins and a tetralone from *Cytospora eucalypticola*. *Phytchemistry* 2003, *62*, 779–782.
141. Gallo, M.B.C.; Cavalcanti, B.C.; Barros, F.W.; de Moraes, M.O.; Costa-Lotufo, L.V.; Pessoa, C.; Bastos, J.K.; Pupo, M.T. Chemical constituents of *Papulaspora immersa*, an endophyte from *Smallanthus sonchifolius* (Asteraceae), and their cytotoxic activity. *Chem. Biodivers*. 2010, *7*, 2941–2950.
142. Evidente, A.; Punzo, B.; Andolfi, A.; Cimmino. A.; Melck, D.; Luque, J. Lipophilic phytotoxins produced by *Neofusicoccum parvum*, a grapevine canker agent. *Phytopathol. Mediterr*. 2010, *49*, 74–79.
143. El-Elimat, T.; Raja, H.A.; Figueroa, M.; Falkinham, J.O.; Oberlies, N.H. Isochromenones, isobenzofuranone, and tetrahydronaphthalenes produced by *Paraphoma radicina*, a fungus isolated from a freshwater habitat. *Phytochemistry* 2014, *104*, 114–120.

144. Sadorn, K.; Saepua, S.; Boonyuen, N.; Boonruangprapa, T.; Rachtawee, P.; Pittayakhajonwut, P. Antimicrobial activity and cytotoxicity of xanthoquinodin analogs from the fungus *Cytospora eugeniae* BCC42696. *Phytochemistry* 2018, *151*, 99–109.
145. Yang, H.X.; Peng, X.P.; Gao, H.; Zhang, H.M.; Wang, Z.R.; Li, G.; Lou, H.X. Pleosporalins H and I, two new heptaketides from the endophytic fungus *Pleosporales* sp. F46 by using OSMAC strategy. *Nat. Prod. Res.* **2021**, *35*, 13307–3313.
146. Yang, X-Y.; Zhang, J-X.; Ding, Q-Y.; He, Z-C.; Zhu, C-Y.; Zhang, K-Q.; Niu, X-M. Metabolites from two dominant thermophilic fungal species *Thermomyces lanuginosus* and *Scytalidium thermophilum*. *Chem. Biodivers.* 2020. *17*, e2000137.
147. Sato, H.; Takishima, T.; Otomo, N.; Sakamura, S. Phytotoxins produced by the fungus of the larch shoot bligh. *Nippon Nôgeikagaku Kaishi*. **1982**, *56*, 649–653.151.
148. Luo, G.; Chen, S.; Yu, J.; Yuan, J.; Zheng, L.; Liu, L.; Chen, B.; Li, J. Naphthalenones and naphthols isolated from the *Saussurea laniceps* endophytic fungus *Didymella glomerata* X223. *Chem. Biodivers*. 2020, *17*, e2000315.
149. Bell, A.A.; Stipanovic, R.D.; Puhalla, J.E. Pentaketide metabolites of *Verticillium dahliae*: Identification of (+)-scytalone as a natural precursor to melanin. *Tetrahedron* **1976**, *32*, 1353–1356.
150. Liu, Y.; Stuhldreier, F.; Kurtan, T.; Mandi, A.; Arumugam, S.; Lin, W.; Stork, B.; Wesselborg, S.; Weber, H.; Henrich, B.; Daletos, G.; Proksch, P. Daldinone derivatives from the mangrove-derived endophytic fungus *Annulohypoxylon* sp. *RSC Adv.* 2017, *7*, 5381–5393.
151. Zhang, J.; Liang, J.H.; Zhao, J.C.; Wang, Y.L.; Dong, P.P.; Liu, X.G.; Zhang, T.Y.; Wu, Y.Y.; Shang, D.J.; Zhang, Y.X.; Sun, C.P. Xylarianins A-D from the endophytic fungus *Xylaria* sp. SYPF 8246 as natural inhibitors of human carboxylesterase 2. *Bioorg. Chem*. 2018, *81*, 350–355.
152. Padumadasa, C.; Xu, Y.M.; Wijeratne, E.M.K.; Espinosa-Artiles, P.; U'Ren, J.M.; Arnold, A.E.; Gunatilaka, A.A.L. Cytotoxic and noncytotoxic metabolites from *Teratosphaeria* sp. FL2137, a fungus associated with *Pinus clausa*. *J. Nat. Prod.* 2018, *81*, 616–624.
153. Bartman, C.D.; Campbell, I.M. Naphthalenone production in *Aspergillus parvulus*. *Can. J. Microbiol*. 1979, *25*, 130–137.
154. Hao, G.; Qing-Hua, Z.; Miao-Miao, J.; Jin-Shan, T.; Cheng-Du, M.; Kui, H.; Michio, N.; Nai-Li, W.; Xin-Sheng, Y. Polyketides from a marine sponge-derived fungus *Mycelia sterilia* and proton-proton long-range coupling. *Magn. Reson. Chem*. 2008, *46*, 1148–1152.
155. Rivera-Chávez, J.; El-Elimat, T.; Gallagher, J.M.; Graf, T.N.; Fournier, J.; Panigrahi, G.K.; Deep, G.; Bunch, R.L.; Raja, H.A.; Oberlies, N.H. Delitpyrones: α-Pyrone derivatives from a freshwater *Delitschia* sp. *Planta Med.* 2019, *85*, 62–71.
156. Wu, Z-C.; Li, D-L.; Chen, Y-C.; Zhang, W-M. A new isofuranonaphthalenone and benzopyrans from the endophytic fungus *Nodulisporium* sp. A4 from *Aquilaria sinensis*. *Helv. Chim. Acta*. 2010, *93*, 920–924.
157. Zhou, Y.H.; Zhang, M.; Zhu, R.X.; Zhang, J.Z.; Xie, F.; Li, X.B.; Chang, W.Q.; Wang, X.N.; Zhao, Z.T.; Lou, H.X. Heptaketides from an endolichenic fungus *Biatriospora* sp. and their antifungal activity. *J. Nat. Prod*. 2016, *79*, 2149–2157.
158. Ariefta, N.R.; Nikmawahda, H.T.; Koseki, T.; Shiono, Y. Fusopoltides B–E, new polyketides isolated from Fusarium solani B-18 *Tetrahedron Lett*. 2019, *60*, 151361.
159. Niu, S.; Tang, X.X.; Fan, Z.; Xia, J.M.; Xie, C.L.; Yang, X.W. Fusarisolins A–E, polyketides from the marine-derived fungus *Fusarium solani* H918. *Mar. Drugs* 2019, *17*, 125.
160. Itsuo, K.; Shunji, S.; Akihiko, M.; Akio, F. 5-Hydroxydihydrofusarubin, a process for its preparation and its use as a medicament. *European Patent Application* 0234431 A2, **1987**.
161. Wen, Y.; Lv, Y.; Hao, J.; Chen, H.; Huang, Y.; Liu, C.; Huang, H.; Ma, Y.; Yang, X. Two new compounds of *Penicillium polonicum*, an endophytic fungus from *Camptotheca acuminata* Decne. *Nat. Prod. Res*. 2020, *34*, 1879–1883.
162. Stodůlková, E.; Man, P.; Kuzma, M.; Černý, J.; Císařová, I.; Kubátová, A.; Chudíčková, M.; Kolařík, M.; Flieger, M. A highly diverse spectrum of naphthoquinone derivatives produced by the endophytic fungus *Biatriospora* sp. CCF 4378. *Folia Microbiol.* (Praha). **2015**, *60*, 259–267.
163. Sakagami, Y.; Sano, A.; Marumo, S.; Yoshikawa, N.; Nakagawa, J. Cladosporol, a plant growth regurator produced by fungus *Cladosporium cladosporioide*s. *Symposium on the chemistry of natural products* **1992**, 134–141. https://doi.org/10.24496/tennenyuki.34.0_134.
164. Sakagami, Y.; Sano, A.; Hara, O.; Mikawa, T.; Marumo, S. Cladosporol, β-l,3-glucan biosynthesis inhibitor, isolated from fungus, *Cladosporium cladosporioides*. *Tetrahedron Lett*. **1995**, *36*, 1469–1472.
165. Zurlo, D.; Ziccardi, P.; Votino, C.; Colangelo, T.; Cerchia, C.; Dal Piaz, F.; Dallavalle, S.; Moricca, S.; Novellino, E.; Lavecchia, A.; Colantuoni, V.; Lupo, A. The antiproliferative and proapoptotic effects of cladosporols A and B are related to their different binding mode as PPARγ ligands. *Biochem. Pharmacol.* **2016**, *108*, 22–35.

166. Nasini, G.; Arnone, A.; Assante, G.; Bava, A.; Moricca, S.; Ragazzi, A. Secondary mould metabolites of *Cladosporium tenuissimum*, a hyperparasite of rust fungi. *Phytochemistry* **2004**, *65*, 2107–2111.
167. Yamazaki, H.; Yagi, A.; Akaishi, M.; Kirikoshi, R.; Takahashi, O.; Abe, T.; Chiba, S.; Takahashi, K.; Iwakura, N.; Namikoshi, M.; Uchida, R. Halogenated cladosporols produced by the sodium halide-supplemented fermentation of the plant-associated fungus *Cladosporium* sp. TMPU1621. *Tetrahedron Lett.* **2018**, *59*, 1913–1915.
168. Zhen, F.; Zhang-hua, S.; Yu-chan, C, Hao-hua L, Wei-min Z. Cladosperanol A, a new dimeric tetralone from marine-derived fungus *cladosporium perangustum*. Nat. Prod. Res. Dev. 2016, 28, 486–489.
